# Population-Based Temporal Trends and Ethnic Disparity in Cervical Cancer Mortality in South Africa (1999–2018): A Join Point and Age–Period–Cohort Regression Analyses

**DOI:** 10.3390/cancers14246256

**Published:** 2022-12-19

**Authors:** Gbenga Olorunfemi, Elena Libhaber, Oliver Chukwujekwu Ezechi, Eustasius Musenge

**Affiliations:** 1 Division of Epidemiology and Biostatistics, School of Public Health, University of Witwatersrand, Johannesburg 2000, South Africa; 2 Faculty of Health Sciences, University of Witwatersrand, Johannesburg 2000, South Africa; 3 Division of Clinical Sciences, Nigerian Institute for Medical Research, Lagos 12003, Nigeria

**Keywords:** APC analysis, age–period–cohort analysis, cervical cancer mortality rate, ethnic disparity of cervical cancer, joinpoint regression, South Africa, Sub-Saharan Africa, gynaecological cancer, cancer trends analysis

## Abstract

**Simple Summary:**

Cervical cancer is a major cause of cancer deaths among women, especially in South Africa. The government has introduced some interventions to reduce the burden of cervical cancer in the country. Our group conducted trend analyses of cervical cancer mortality from 1999–2018 to investigate if the interventions have reduced the burden. Our study showed that cervical cancer mortality increased from 1999 to 2018 and is more common among Black South Africans as compared to non-Blacks. More public health interventions are necessary to reduce the trends in South Africa.

**Abstract:**

Cervical cancer is one of the leading causes of cancer deaths among women in low- and middle-income countries such as South Africa. The current impact of national cervical cancer control and sexual and reproductive health interventions in South Africa reduce its burden. The aim of this study was to assess the trends in cervical cancer mortality and its relation to breast and gynaecological cancers in South Africa from 1999 to 2018. We conducted joinpoint regression analyses of the trends in crude and age-standardised mortality rates (ASMR) for cervical cancer mortality in South Africa from 1999 to 2018. An age–period–cohort regression analysis was also conducted to determine the impact of age, period, and cohort on cervical cancer mortality trends. Analyses were stratified by ethnicity. Cervical cancer (*n* = 59,190, 43.92%, 95% CI: 43.65–44.18%) was responsible for about 43.9% of breast and gynecological cancer deaths. The mortality rate of cervical cancer (from 11.7 to 14.08 per 100,000) increased at about 0.9% per annum (Average Annual Percent Change (AAPC): 0.9% (AAPC: 0.9%, *p*-value < 0.001)), and young women aged 25 to 49 years (AAPC: 1.2–3.5%, *p*-value < 0.001) had increased rates. The risk of cervical cancer mortality increased among successive birth cohorts. In 2018, cervical cancer mortality rate among Blacks (16.74 per 100,000 women) was about twice the rates among Coloureds (8.53 deaths per 100,000 women) and approximately four-fold among Indians/Asians (4.16 deaths per 100,000 women), and Whites (3.06 deaths per 100,000 women). Cervical cancer control efforts should be enhanced in South Africa and targeted at ethnic difference, age, period, and cohort effects.

## 1. Introduction

Cervical cancer is the fourth leading cause of cancer deaths and was responsible for about 342,000 global cancer deaths in 2020 [1]. More than 80% of cervical cancer deaths occurred in low- and middle-income countries (LMIC) [1]. Indeed, the age-standardised incidence (ASIR) and mortality rate (ASMR) of cervical cancer are high in LMICs when compared to the rates in high-income countries (HICs) [1]. The ASMR of cervical cancer in LMICs is generally between 8.5 and 30 deaths per 100,000 women, while the ASMR was between 1.7 and 7 deaths per 100,000 women in the HICs [2]. Cervical cancer had the highest ASMR of 19.6 per 100,000 women among South African women [1]. Both cervical and breast cancers constitute about 85% of breast and gynaecological cancers in South Africa [1].

Persistent, high-risk human papilloma virus (HPV) infection is the necessary cause of cervical cancer [1,3,4]. Therefore, risky sexual and reproductive behaviour such as an early age at coitarche, multiple sexual partnerships, unprotected sexual intercourse, early age at first pregnancy, and high parity are implicated in the evolution of cervical cancer [1,3,4,5]. Smoking and prolonged use of oral contraceptive pills are co-factors for the evolution of cervical cancer [1,3,6]. Availability of cervical cancer-screening facilities and prompt treatment of cervical cancer cases will increase the survival rates, thereby reducing the mortality rates. In contrast to HICs, most LMICs have poor cervical cancer-screening facilities [1,3,4,5].

South Africa is a middle-income country with four major ethnic groups (Black African, White, Coloured, and Indian/Asian [7]. There has been a rapid expansion in access to sexual and reproductive health services in the public sector of South Africa following the commencement of a democracy in 1994 [8,9]. Thus, an evaluation of the impact of previous public health interventions on the trends in cervical cancer mortality in South Africa is necessary to aid in further planning [7,10]. South Africa has one of the highest prevalence of human immunodeficiency virus (HIV) globally [11]. Thus, the burden of cervical cancer is considerable in South Africa because cervical cancer is an AIDS-defining illness [12,13]. Although the advent of anti-retroviral therapy (ART) is expected to reduce the prevalence of and death from cervical cancer, evidence still suggests an increased risk of cervical cancer in older HIV-positive women [14].

Cervical cancer prevention can be primary, secondary, or tertiary. Primary prevention includes the prevention of HPV infection through the population-based vaccination of both boys and girls and the entrenchment of safe sexual practices such as consistent use of condoms [15]. Secondary prevention entails PV infection screening, Papanicolaou screening, and visual inspection with acetic acid or Lugol’s iodine to detect pre-invasive cervical cancer lesions. Pre-invasive cancer lesions can be easily treated with cryotherapy, large loop electrosurgical excision biopsy, cone biopsy, or hysterectomy to prevent cervical cancer. Countries that have promoted the preventative interventions have had a dramatic reduction in cervical cancer incidence and death [15]. Tertiary prevention of cervical cancer entails the various treatment modalities of early- and late-stage cervical cancer. Various surgical interventions (on a spectrum of less radical to radical types), chemoradiation, and palliative care are the mainstays of treatment [15]. The burden of cervical cancer mortality is closely related to access to prevention programs and optimum oncological services.

Some public health initiatives in South Africa–such as the free, population-based routine Papanicolou smear test that commenced in 2002, the free HPV vaccination of schoolgirls beginning in 2014, promotion of condom use, the expansion of and easy access to oncological services, the national roll-out of free anti-retroviral treatment (ART) in 2004 and subsequent national and global HIV-control policies, tobacco-control programmes following the establishment of a multi-racial democracy in 1994, and multiple cervical cancer control policies–can lead to a “period effect” on the trends in cervical mortality in South Africa [7,10]. The various changes in the political, socio-economic and reproductive behaviours of South African women during the apartheid and post-apartheid eras can engender cohort-specific risks of cervical cancer mortality among women born around the same time [16]. Joinpoint regression and age–period–cohort (A–P–C) modelling are important statistical tools that aid in the understanding of cancer trends. Although joinpoint regression modelling was previously utilised for cervical cancer trends in South Africa, no previous study in South Africa and Sub-Saharan Africa has utilised both joinpoint and A–P–C regression modelling [7]. The national mortality data collected by Statistics South Africa is a very rich source of data for A–P–C modelling [17]. The aim of this study was to assess the national and ethnic trends in cervical cancer mortality in South Africa from 1999 to 2018 by utilising both joinpoint and A–P–C regression modelling to inform policy. Trends in the proportion of cervical cancer to breast and gynaecological cancers were also assessed.

## 2. Materials and Methods

Our group conducted a trend analysis of cervical cancer mortality in South Africa from 1999 to 2018. In 2018, about 80.4% of South Africans were Blacks, while 8.9%, 8.3%, and 2.4% were Coloureds (mixed ancestry), Whites (European descent) and Indians/Asians, respectively [18].

### 2.1. Data Source

Statistics South Africa (Stats SA) usually collates and publishes annual national and sub-national mortality data [17,18]. Information on cervical cancer mortality was retrieved from the Stats SA website [18]. Cervical cancer mortality was coded as C53 according to the International Classification of Diseases, Tenth Revision (ICD10) [1,7]. The annual mid-year, 5-year (15–19, 20–24, 25–29, etc.) population estimates of females older than 15 years were obtained from the Stats SA website. The intercensal rate between the census of 1996 and 2001 was used to calculate the annual mid-year population estimates from 1999 to 2001. The quality of the mortality data as collected by Stats SA is comprehensive, complete, and timely [17,19]. The vital registrations of South Africa and two other countries were found to be of high quality in Africa [17,19].

### 2.2. Ethical Approval

Prior to the commencement of the study, ethical approval was obtained from the Human Research Ethics Committee (Medical) of the University of the Witwatersrand (Clearance certificate number: M190544). The mortality data was publicly available. This study was conducted according to the guidelines of the Declaration of Helsinki.

### 2.3. Statistical Analysis

Data validation and data cleaning were conducted. Categorical variables were described as frequency and percentages while continuous variables were described as mean (± standard deviation). The annual prevalence of cervical cancer mortality among all breast and gynaecological cancer deaths was calculated.

### 2.4. Annual Crude and Age-Standardised Rates

The overall and ethnic annual crude mortality rates (CMR) were calculated by dividing the annual cervical cancer mortality rate by the mid-year female population (≥15 years). The direct method of standardisation was conducted based on the Segi’s world standard population.

(1) $$\mathrm{Age}-\mathrm{standardised}\;\mathrm{rates}\;\mathrm{were}\;\mathrm{given}\;\mathrm{by}\;\frac{\sum_{i=1}^{A}a_{i}w_{i}}{\sum_{i=1}^{A}w_{i}}\times 100,000,$$

where $a_{i}$ is the age-specific rate of the *i*th 5-year age group and $w_{i}$ is the corresponding number of persons (or the weight) in the 5-year age group i of the Segi’s world standard population.

### 2.5. Join Point Regression Modelling

The trend analysis of the annual overall and ethnic cervical cancer mortality was conducted using the Joinpoint regression software, version 4.8.0.1 (Statistical Methodology and Applications Branch, Surveillance Research Program, National Cancer Institute, Bethesda, MD, USA). Poisson regression was modelled with ln (cervical cancer mortality rate) as the outcome while the year of death was the explanatory variable. Four maximum Join points and 4499 Monte Carlo permutation tests were conducted for the significance of the mortality trends.

The segmental annual percent change (APC) and the overall average annual percent change (AAPC) were calculated. The interpretation of the Joinpoint model was as follows: positive, or negative AAPC (or APC) with *p*-value < 0.05 showed a statistically significant increased or decreased trend. If the *p*-value of the AAPC was >0.05, the trend was interpreted as a non-significant increased or decreased trend. If the AAPC was between −0.5 and +0.5 with *p*-value > 0.05, the trend was reported as stable.

### 2.6. Age–Period–Cohort Modelling of Cervical Cancer Mortality

The A–P–C modelling involves disentangling the individual effects of age, period, and cohort.

The relationship between age, period, and birth cohort is

Birth cohort = period − age(2)

and the general multiplicative equation of the A-P-C model is expressed as

ln (MR) = A + P + C + ε (3)

where MR is the mortality rate, A is the 5-year age group (15–19, 20–24, 25–29…75+ years), P is the period (1999–2003, 2004–2008, 2009–2013, and 2014–2018), C is the cohort effect of the population, and ε is the intercept.

### 2.7. Identifiability Problem

There is a perfect linear relationship between age, period, and cohort, leading to the identifiability problem due to perfect collinearity during regression modelling [16]. To address the identification problems, our group produced estimable parameters based on the Age Period Cohort Web Tool (Biostatistics Branch, National Cancer Institute, Bethesda, MD, USA) (Age Period Cohort Analysis Tool (cancer.gov (accessed on 24 April 2022)), https://github.com/CBIIT/nci-webtools-dceg-age-period-cohort (accessed on 24 April 2022) [20].

A lexis matrix was built by categorizing the age of the cervical cancer mortality data into 5-year age groups from 15 years to 75 years (15–19 years, 20–24 years, 25–29 years, 30–34 years, 35–39 years, 35–39 years, 40–44 years, 45–49 years, 50–54 years, 55–59 years, 60–64 years, 65–69 years, 70–74 years, and 75 years and above) and year of death (calendar period) into the 5-year category from 1999 to 2018 (1999–2003, 2004–2008, 2009–2013, and 2014–2018). The diagonal of the matrix was the corresponding birth cohort. The data was then imported into the A–P–C webtool to obtain the estimable parameters [20].

The net drift (equivalent to the AAPC), local drift (equivalent to the APC per age group), longitudinal age-specific rates, cohort rate ratio (RR), and period rate ratio (RR) were estimated. The middle categories of the period (2004–2008) and cohort estimates (1959–1963) were taken as references. Statistical significance was assessed with Wald’s test. A two-tailed test of hypothesis was assumed, and a 95% confidence interval was taken as the statistically significant level. Analysis was conducted for overall cervical cancer mortality and then stratified by ethnicity. Stata (Statcorp, TE, USA) version 16 and R-version 3.6.3 (R-foundation, Vienna, Austria) statistical software were utilised.

## 3. Results

Cervical cancer (*n* = 59,190, 43.9%, 95% CI: 43.7–44.2%) was responsible for about 43.9% of breast and gynecological cancer deaths and the annual prevalence of cervical cancer mortality among breast and gynecological cancer mortality was about 46.2% in 1999 and 42.7% in 2018 (Table 1).

### 3.1. Trends in Cervical Cancer Mortality

**Figure 1:**
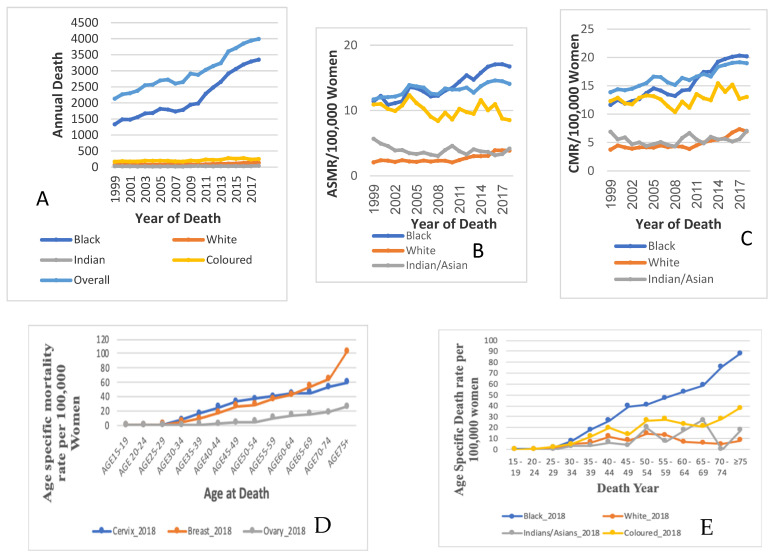
A. Overall age-specific death rates in 2018 in South Africa from (**A**) breast and cervical cancer. National and ethnic annual deaths: (**A**), age-standardised mortality rates; (**B**) crude mortality rates; (**C**) age-specific mortality rates; (**D**) Comparison of age-specific death rates for cervical cancer, breast cancer, and ovarian cancer in South Africa, 2018; and (**E**) rates of cervical cancer in South Africa (1999–2018).

**Figure 2:**
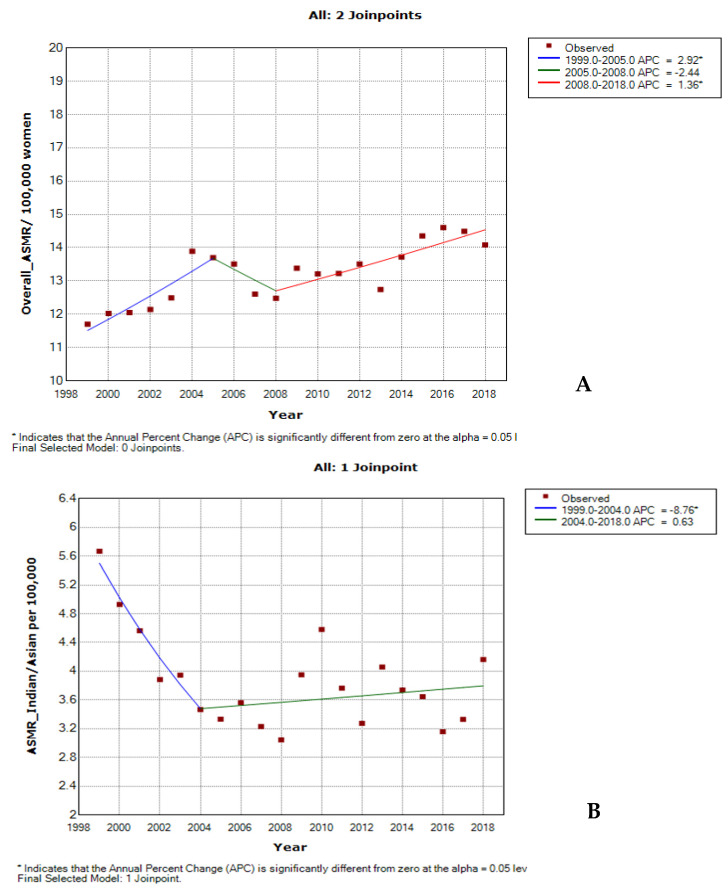

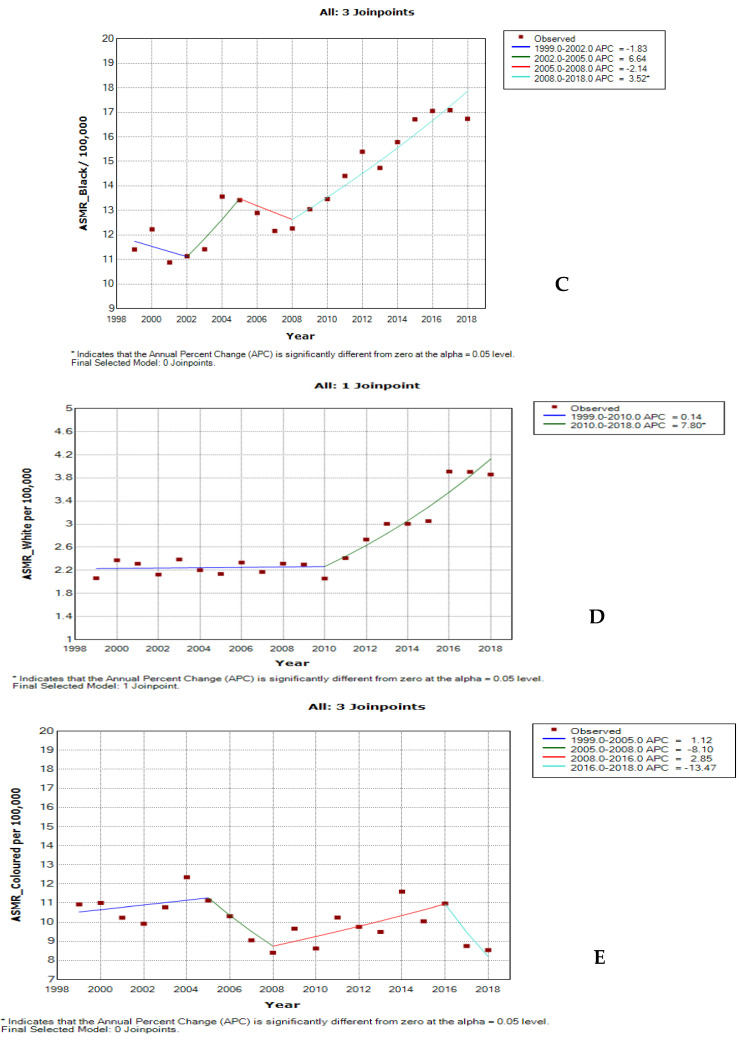
Joinpoint regression trends of the annual age-standardised mortality rate for cervical cancer in South Africa (1999–2018) for: (**A**) overall; (**B**) Indians/Asians; (**C**) Blacks; (**D**) Whites; and (**E**) Coloured ethnic groups.

The annual deaths and ASMR of cervical cancer increased from 2134 deaths in 1999 to 3993 deaths in 2018 (Figure 1, Table 1). The ASMR increased by 0.9% (AAPC: 0.9%, 95%; CI: 0.6–1.2; *p*-value < 0.001) per annum from 11.7 deaths per 100,000 women in 1999 to 14.1 deaths per 100,000 women in 2018 (Figure 1B). A Joinpoint regression analysis of cervical cancer ASMR showed three trends. The first was an increased trend of about 2.9% per annum from 1999 to 2005 (APC: 2.9%, *p*-value < 0.001). Subsequently, there was a non-statistically significant decline at an annual rate of 2.4% from 2005 to 2008 (APC: −2.4%, *p*-value = 0.6) and then a rise in rates of 1.4% per annum (APC: 1.4%, *p*-value < 0.001) from 2008 to 2018 (Figure 2A, Table 2).

### 3.2. Ethnic Trends of Cervical Cancer Mortality

The annual deaths from cervical cancer among Black women were the highest throughout the study period, ranging between 1336 and 3347 deaths. However, cervical cancer deaths among each of the other ethnic groups were relatively very low (Coloureds ~170–250 deaths; Whites ~70–140 deaths; Indians/Asians ~30–40 deaths) (Figure 1A, Supplementary Table S1).

In 2018, the Black ethnic group had the highest ASMR of cervical cancer (16.7 per 100,000 women), with a rate of about twice that among the Coloured group (8.5 deaths per 100,000 women) and about four-fold the rates among the Indian/Asian group (4.2 deaths per 100,000 women) and White group (3.1 deaths per 100,000 women) (Figure 1B, Supplementary Table S1). Though Whites had the lowest ASMR from cervical cancer, they had the most rapid rise in mortality rate (AAPC: 3.3%, *p*-value < 0.001), followed by Blacks (AAPC: 2.4%, *p*-value < 0.001). In contrast, the Indian/Asian group had declining rates of about 1.4% per annum (AAPC: −1.4%, *p*-value < 0.001), while the Coloured population group had a non-statistically significant slight decline in rates of about 0.7% per annum (AAPC: −0.7, *p*-value = 0.1) (Figure 2B–E, Table 2).

### 3.3. Joinpoint Trends in the Overall Age-Specific Mortality Rates for Cervical Cancer, 1999–2018

**Figure 3:**
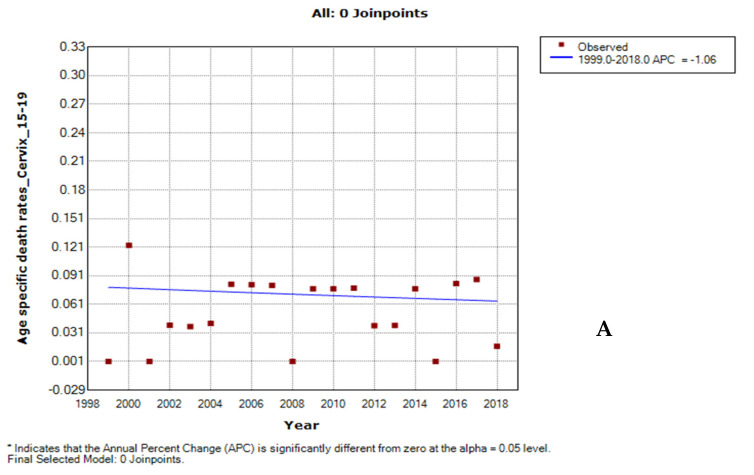

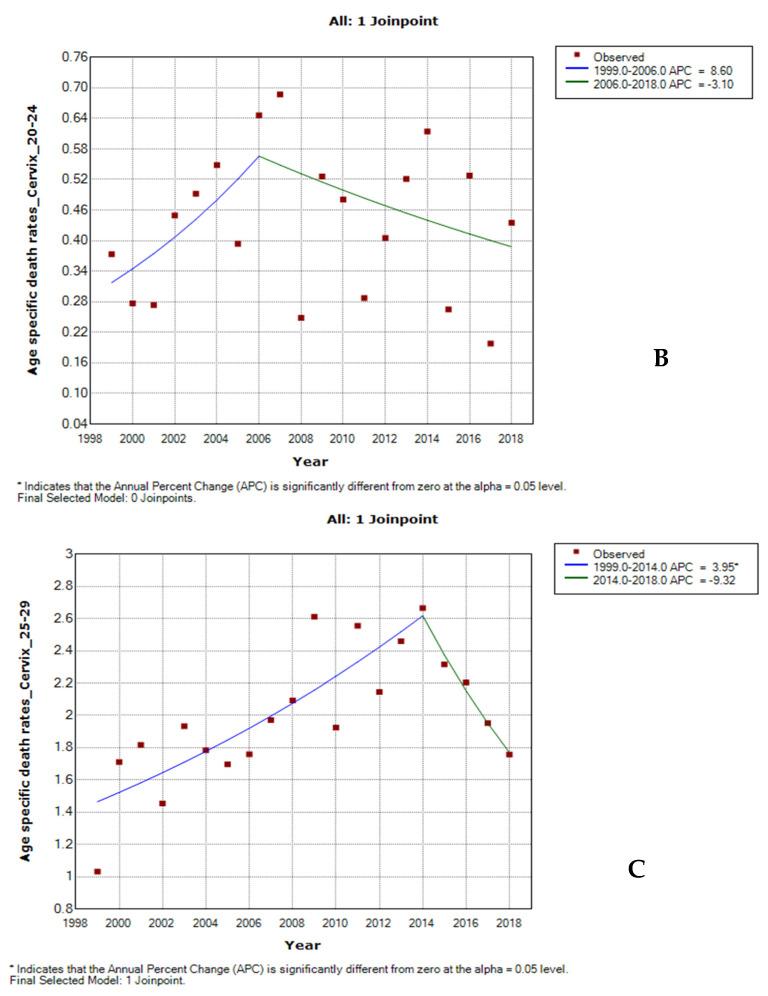

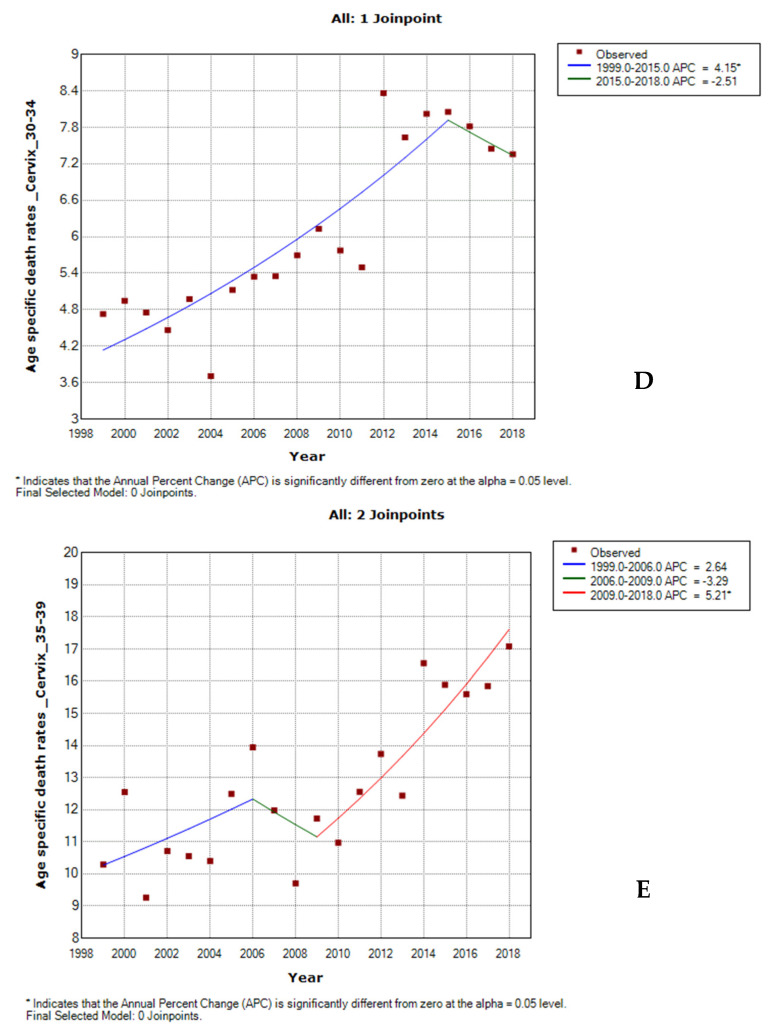

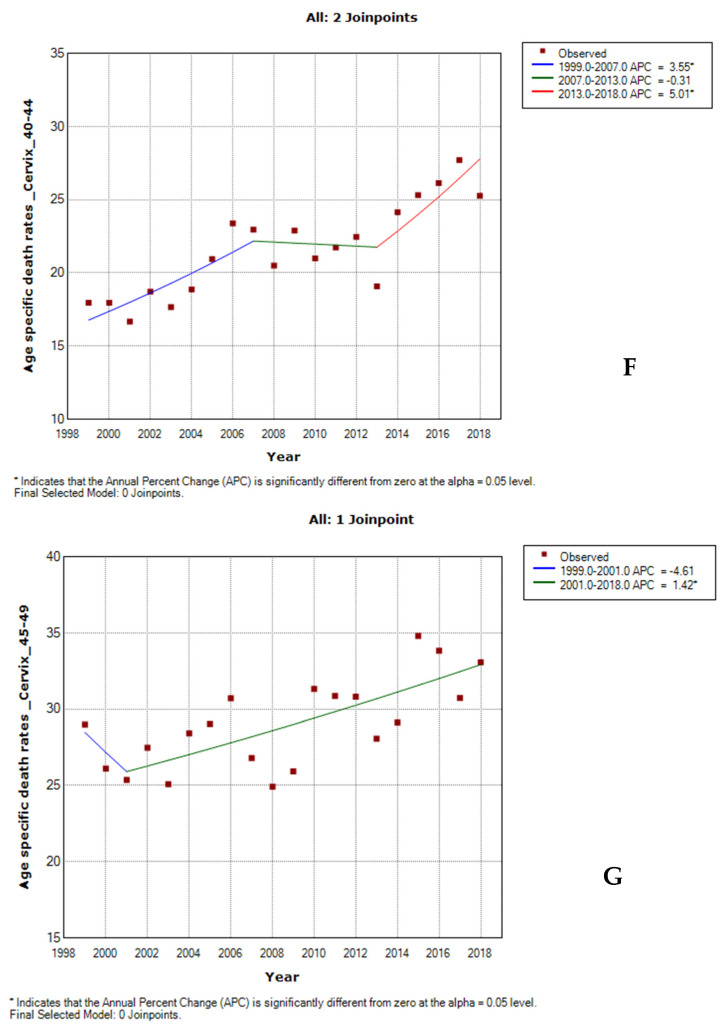

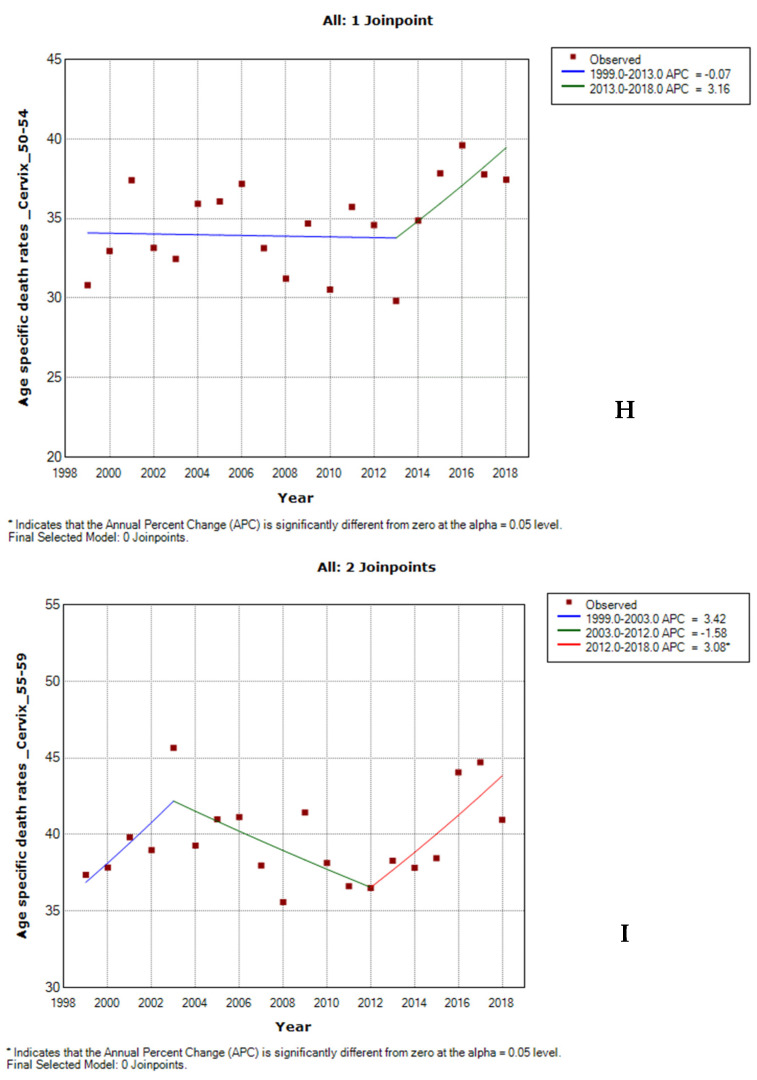

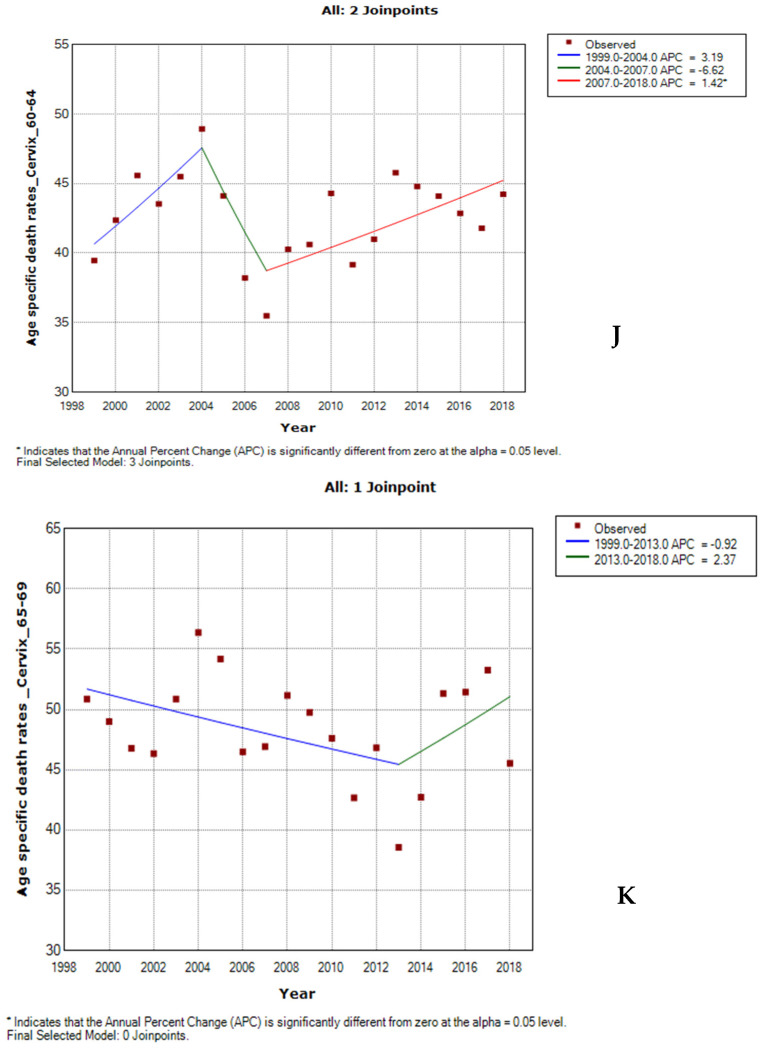

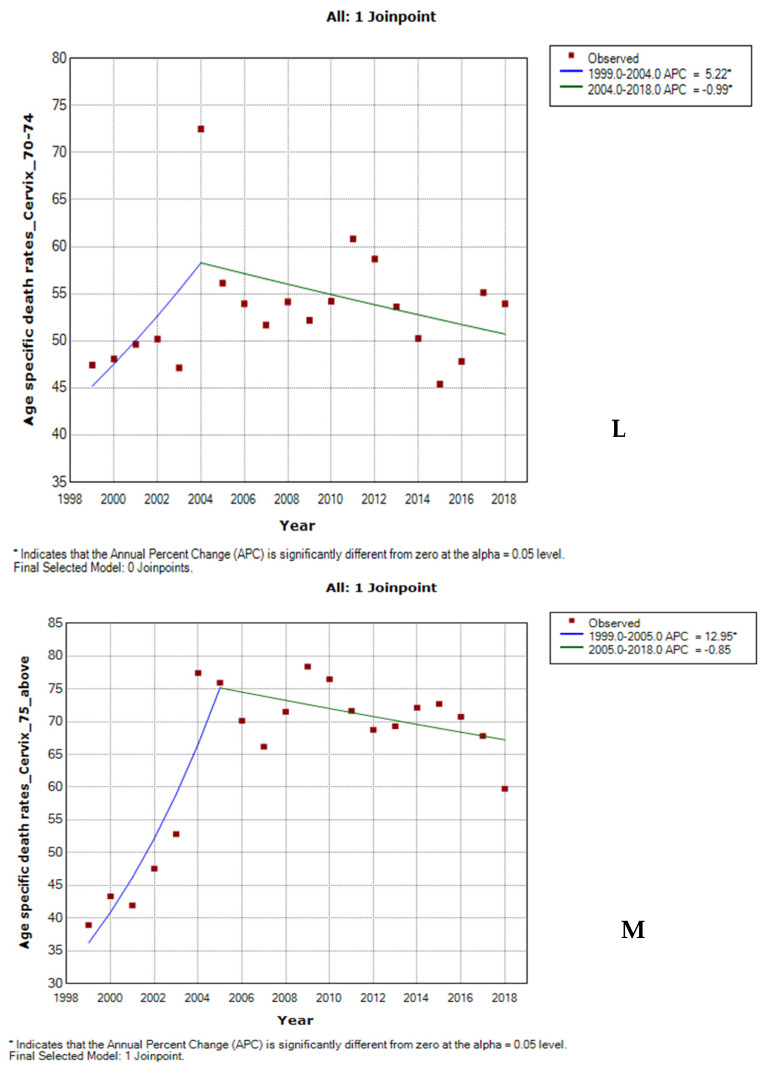
Joinpoint trends of age-specific death rates of cervical cancer in South Africa, 1999–2018. (**A**) 15–19 years (**B**) 20–24 years (**C**) 25–29 years (**D**) 30–34 years (**E**) 35–39 years (**F**) 40–44 years (**G**) 45–49 years (**H**) 50–54 years (**I**) 55–59 years (**J**) 60–64 years (**K**) 65–69 years (**L**)70–74 years (**M**) 75 years and above.

Between 1999 and 2018, teenagers (APC: −1.1%, *p*-value = 0.6) had non-significant declining cervical cancer rates while women in the reproductive age group of 25 to 49 years and women aged 75 years and older had increased rates (AAPC range: 1.2–3.5%, *p*-value < 0.001), with women aged 30–34 years having the highest rise. Although young women aged 20–24 years and those aged 50–74 years had overall stable rates (AAPC range: −0.3 to 0.5, *p*-value > 0.05), women aged 50– 69 years had increased rates from 2013 to 2018 and women aged 70 years and older had declining rates from 2005 to 2018 (Figure 3, Supplementary Table S2).

### 3.4. Trends in Mean Age and Age-Specific Rates of Cervical Cancer Mortality by Ethnicity

In 2018, the mean age at death from cervical cancer in South Africa was 55.2 ± 14.8 years. The mean age at death from cervical cancer was between 55 and 56 years during the study period of 1999–2018 (Table 1). In 2018, the mean age at death from cervical cancer was the lowest among Coloureds (54.8 ± 14.5 years), followed by Whites (55.0 ± 14.4 years) and Blacks (55.2 ± 14.4 years), while Indians/Asians (56.9 ± 13.2 years) had the highest mean age at death. The mean age at death from cervical cancer did not change dramatically across the ethnic groups (Supplementary Table S1).

### 3.5. Age-Specific Death Rate from Cervical Cancer by Ethnicity

In 2018, the Black ethnic group had the highest mortality rate amongst all the age groups and, beginning in the teenage age group, their mortality rates were observed to increase with age. The Coloured population group had the second-highest rates and their mortality was reported from 24 to 29 years to 75 years as two peaks which occurred at 40–44 years and 55–59 years. The White and Indian/Asian groups had relatively low age-specific mortality rates for cervical cancer, with two peaks having occurred at 40–44 years and 50–54 years (Figure 1, Supplementary Table S3).

### 3.6. Joinpoint Trends of Age-Specific Death Rates for Cervical Cancer by Ethnicity

White, Coloured, and Indian/Asian ethnic groups had few data points for women aged 15–19 years and <30 years, respectively. From 1999 to 2018, White women aged 30–64 years had increased age-specific mortality rates for cervical cancer with the youngest age group having the highest rise (AAPC range: 5.2% to 7.2%, *p*-value < 0.001), while those aged 55–64 years had a non-significant rise. Whites aged 20–24 years and 75 years and above had reduced rates (AAPC: −2.1 to −1.4, *p*-value < 0.001) while those aged 25–29 years (AAPC: 0.2, *p*-value = 0.9) had stable rates. From 2014 to 2018, White women aged 50–59 years and 65–69 years showed a statistically significant rise in cervical cancer mortality rates (APC: 7.7% to 27.0%, *p*-value < 0.001) (Supplementary Figure S1, Supplementary Table S2).

From 1999 to 2018, Coloureds aged 25–34 years had a significant rise in cervical cancer death rates (AAPC range: 3.6–5.2, *p*-value < 0.001), while Coloureds aged 50–54 years (AAPC: −1.7, *p*-value < 0.001) and 70–74 years (AAPC: −2.1, *p*-value <0.001) had significant declining rates. Women aged 35–39, 40–44 and 55–69 years had non-significant decline, while those aged 45–49 and 75 years and older had stable rates (Supplementary Figure S2, Supplementary Table S2).

All Indians/Asians aged 30 years and older had declining mortality rates, but only those aged 65–74 years showed a statistically significant decline. However, those aged 40–44 years had a non-significant rise in mortality rates (AAPC: 1.5%, *p*-value = 0.4) (Supplementary Figure S3, Supplementary Table S2). With the exception of a non-significant increase of 1.4% among young Black women aged 20–24 years (AAPC: 1.4%, *p*-value = 0.4), all other age groups had a statistically significant rise in mortality rates for cervical cancer (AAPC range: 1.6% to 5.0%, *p*-value < 0.001) from 1999 to 2018. However, young women aged 25–39 years (APC range: −7.2% to −3.6%, *p*-value > 0.05) and 35–39 years (APC: −0.1, *p*-value = 1.0), respectively, showed non-significant decline and stable rates from 2014 to 2018 (Supplementary Figure S4, Supplementary Table S2).

### 3.7. Age–Period–Cohort of Overall and Ethnic Trends in Cervical Cancer Mortality

#### 3.7.1. Local and Net Drift

**Figure 4:**
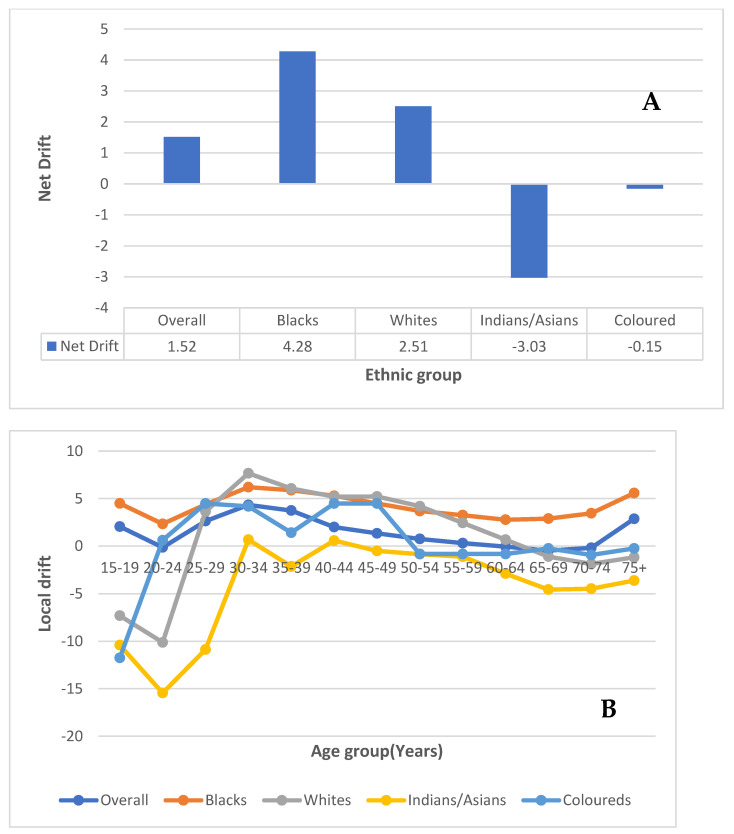
Net (**A**) and local drift (**B**) of cervical cancer mortality in South Africa (1999–2018). (National and ethnic trends).

After correcting for cohort and period effect, the overall drift of cervical cancer mortality from 1999 to 2018 was about 1.5% per annum (95% CI: 1.2–1.9%) (Supplementary Table S4, Supplementary Figure S5A, Figure 4A). Blacks (4.3%, 95% CI: 3.8% to 4.7%) and Whites (2.5%, 95% CI: −0.1% to 5.2%) had a positive net drift while Indians/Asians (−3.0%, 95% CI: −6.5% to 0.6%) and Coloureds (−0.2%, 95% CI: −1.6% to 1.3%) had a non-significant negative drift (Supplementary Table S5, Supplementary Figure S5B–E, Figure 4A).

The local drift of cervical cancer mortality showed values <0 (though largely not statistically significant) for women aged 20–24 years and those aged 60–74 years. The local drift increased with age from 25 years to reach a peak of 4.3% (95% CI:3.6–5.1%) per annum at 30–34 years and subsequently declined with increasing age to −0.5%, (95% CI: −0.95% to −0.01%) per annum at 64–69 years (Supplementary Table S4, Supplementary Figure S5A, Figure 4B). All the local drifts for Blacks were above 0 and the pattern depicted an inverted U-shaped curve from 20 years to a peak drift at 30–34 years and a subsequent decline to 64 years. Young Coloureds (<20 years) and Whites (<25 years) had local drifts < 0 and the ethnic trends generally depicted an inverted U-curve with varying peaks occurring at different ages (Whites: peak drift of 6.1% at 35–39 years; Coloureds: peak drift of 4.5% at 25–29 years). Whites subsequently had a decline in drift to nearly 0 at 65–69 years with a further negative dip from 70 years onwards. For Coloureds, there was a steep decline to −0.7% at 40–44 years and all Coloureds older than 44 years had a drift of <0. All the local drift for Indians/Asians was less than 0 and were not statistically significant (except for a slight positive drift at 30–34 years (0.7%) and 40–44 years (0.6%)) and depicted an inverted U-shaped trend with a peak at 30–34 years (Supplementary Table S5, Supplementary Figure S5B–E, Figure 4B).

#### 3.7.2. Age Effect

**Figure 5:**
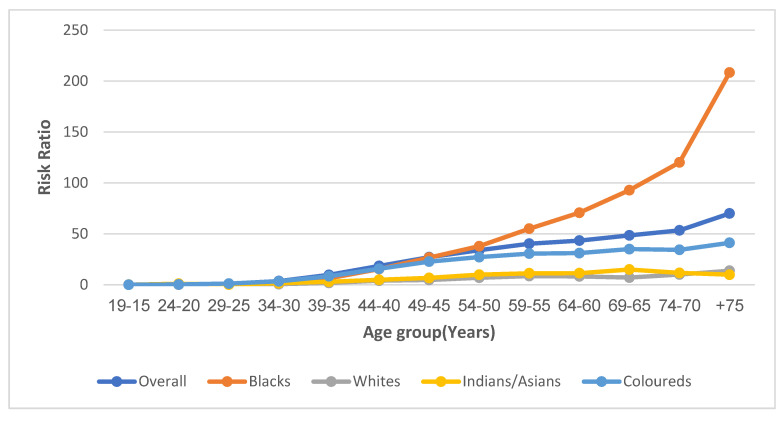
Age effect (Risk ratio) of the National and ethnic cervical cancer mortality in South Africa (1999–2018).

Based on the longitudinal age curve, the RR of the overall and ethnic trends in cervical cancer mortality increased with age (Supplementary Table S4, Figure 5). The lowest rates were among Indians/Asians (RR at 30 years: 1.5; RR at 75 years: 9.9) followed by Whites (RR at 30 years: 0.6; RR at 75 years: 13.8), Coloureds (RR at 30 years: 3.4; RR at 75 years: 41.2) and Blacks (RR at 30 years: 2.3; RR at 75 years: 208.4) (Supplementary Table S5, Supplementary Figure S5B–E, Figure 5).

#### 3.7.3. Period Effect

**Figure 6:**
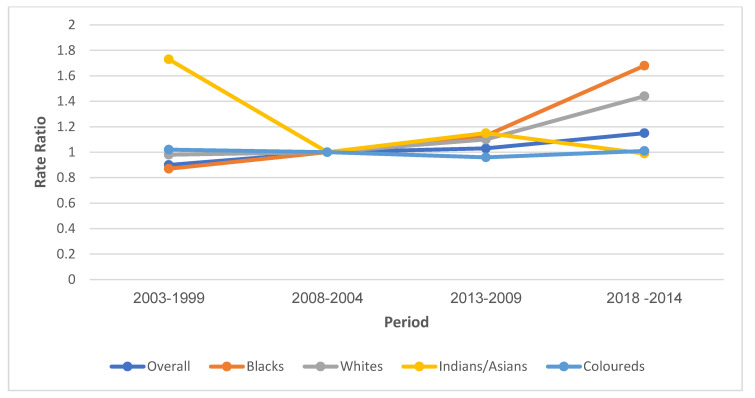
Period Risk ratio of the National and ethnic cervical cancer mortality trends in South Africa (1999–2018).

The overall period RR for cervical cancer mortality increased from 1999 to 2018. The period RR increased by 10% from 1999–2003 to 2004–2008 (RR: 0.9, 95% CI: 0.86–0.94), 3% from 2004–2008 to 2009–2013 (RR: 1.03, 95% CI: 0.99–1.07) and 12% (RR: 1.15, 95% CI: 1.10–1.20) from 2009–2013 to 2014–2018 (Supplementary Table S4, Supplementary Figure S5A, Figure 6). The Blacks (RR from 0.87 to 1.68) and Whites (RR from 0.98 to 1.44) generally experienced an increased period RR with an increase in year from 1999 to 2018. Coloureds had a decline in period RRs from 1.02 between 1999 and 2003 to 0.96 between 2009 and 2013 and subsequently had a slight increase to 1.01 between 2014 and 2018. Indians/Asians had a period RR decline of about 74% from 1999–2003 to 2004–2008 and a subsequent increase (RR: 1.15) from 2009 to 2013, followed by a decline (RR: 0.99) during the 2014–2018 period. (Supplementary Table S5, Supplementary Figure S5B–E, Figure 6). The Wald’s test showed that the period effects on cervical cancer mortality trends were statistically significant for the overall and all ethnic groups except Coloureds (Supplementary Table S6).

#### 3.7.4. Cohort Effect

**Figure 7:**
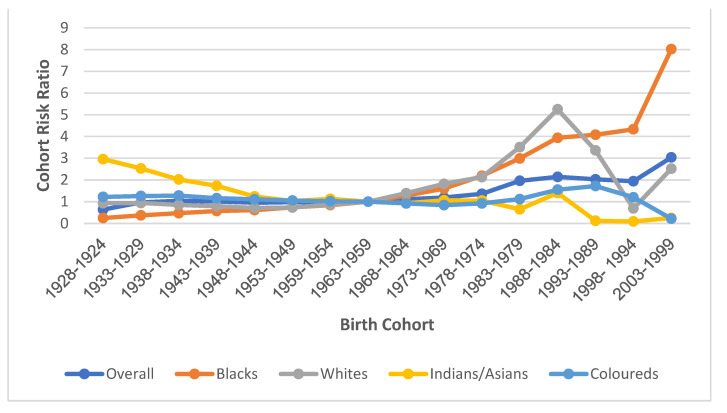
Cohort Risk ratio of the National and ethnic cervical cancer mortality trends in South Africa (1999–2018).

The cohort mortality risk for cervical cancer among those born between roughly 1924 and 1928 (RR: 0.64, 95% CI: 0.57–0.72) was the lowest. This risk increased among successive cohorts, with cohorts born between 1969 and 1973 (RR: 1.19, 95% CI: 1.12–1.26) having about twice the initial risk. The risk then dramatically increased to more than thrice the initial risk among the most recent birth cohort, those born between 1999 and 2003 (RR: 3.04, 95% CI: 1.00–9.27) (Supplementary Table S4, Supplementary Table S5A, Figure 7). With respect to ethnic cohort variations, the Black and White birth cohorts of 1924–1928 had the least (RR: 0.25, 95% CI: 0.22–0.29) and second lowest (RR: 0.98 (95% CI: 0.81–1.20) RR, respectively, with their risks increasing among successive birth cohorts to gradually become the highest, with peak RRs of 8.02 and 5.25 among birth cohorts between 1999–2003 and 1984–1988, respectively (Supplementary Table S5, Supplementary Figure S5B–E, Figure 7).

The cohort RR of cervical cancer mortality among Coloureds slightly declined among successive cohorts from 1924 to 1973 (RR: 1.22 to 0.82), after which the RR increased to a peak RR (1.72) among the 1989–1993 cohort. RRs then declined among successive cohorts of Coloureds to 0.22 for the 1999–2003 cohort. Indians/Asians had the highest RR (2.96, 95% CI: 1.31–6.72) among women born between 1924 and 1928. The RR for this group decreased among successive cohorts to become the least ethnic RR among the birth cohorts of 1979–2003 (Supplementary Table S5, Supplementary Figure S5B–E, Figure 7). The Wald’s test showed that cohort effect was statistically significant for overall and among all the ethnic groups except for Indians/Asians (Supplementary Table S6).

## 4. Discussion

For the first time, our group conducted a national and ethnic trends analysis of cervical cancer mortality in South Africa from 1999 to 2018 using both joinpoint and A–P–C regression modelling to guide future public health interventions.

### 4.1. Cervical Cancer Mortality Trends

Our study showed that South Africa had a huge burden of cervical cancer mortality with a 7-fold higher mortality rate when compared to North America (14.1 vs. 2.1 per 100,000 women) [21]. Furthermore, the mortality-to-incidence ratio (MIR) of cervical cancer in South Africa (14.1 vs. 20.3 per 100,000 women, MIR: 0.7) [7,22] was higher than the average of other Southern African countries (20.6 vs. 36.4 per 100,000 women, MIR: 0.6) and the North American region (2.1 vs. 6.2 per 100,000 women, MIR: 0.3), but lower than the average MIR among Western African countries (16.6 vs. 23.0 per 100,000 women, MIR: 0.7) [21]. This pattern showed that the cervical cancer survival rate in South Africa was lower than the average survival rate in Southern African and North American regions. However, cervical cancer survival rates in South Africa appeared to be higher than in the Western African region. There was strong period effect of the cervical cancer mortality trend in South Africa, with the net drift indicating a rise of about 1.5% from 1999 to 2018. However, only about 30 countries largely located in Southern Africa, East Asia, and Eastern Europe reported increased cervical cancer rates out of 203 global countries/territories [7,21,23,24,25,26,27,28]. The regional and national differences in cervical cancer mortality trends were mainly related to the commencement of organized Pap smear screenings, increased drivers of persistent high-risk HPV infection (such as sexual and reproductive behaviors and the prevalence of sexually-transmitted infection and HIV infection) and the availability of prompt oncological care [1,7,21,24].

We further reported a 10% increased period RR of cervical cancer mortality from 1999 to 2003, a reduced risk of about 3% from 2004 to 2013, and a final dramatic rise of 12% from 2014 to 2018. An earlier-reported cervical cancer incidence trend in South Africa from 1994 to 2009 tended to mirror the mortality pattern from 1999 to 2018 [7]. As was previously described, the initial rise in cervical cancer mortality from 1999 to 2005 may be related to the increased prevalence and death from HIV infection in the country, as cervical cancer is an AIDS-defining illness [7,12,29,30]. The decline from 2005 to 2008 may be partly attributed to the roll-out of free ART in the country beginning in 2004 and the initiation of population-based Pap smear in the country beginning in 2000, both of which can lead to down-staging [7,29,31]. South Africa has one of the highest prevalence of HIV globally. During the pre-ART era (before 2005) in South Africa, HIV was a major competing cause of death [18]. However, there was generally reduced HIV-related mortality, improved quality of life, and increased longevity among HIV-positive women after the introduction of ART in South Africa in 2005 [18,29,32,33]. Furthermore, the decline in cervical cancer mortality might be due to an improvement in the healthcare system of the country coupled with increased access to healthcare following the commencement of a multi-racial democracy about ten years earlier in 1994 [9,29,34]. Reduction in risk factors can also lead to a reduction in mortality. The subsequent increase in mortality rate from 2008 to 2018 may be partly due to increased cervical cancer incidence and mortality among older HIV-positive and negative women in older age groups, as well as poor screening coverage [7,14]. Efforts aimed at increasing access to cervical cancer screening, sexual and reproductive health, and oncological services coupled with free ART in the country is very necessary [7]. The impact of the national HPV vaccination program among young schoolgirls which began in 2014 should be evaluated in future studies.

### 4.2. Age Effect of Cervical Cancer Mortality Trends in South Africa

Our group found that from 1999 to 2018 women aged 25–60 years had an increased rate of cervical cancer mortality, with a rapid rise of about 4.3% per annum occurring among women aged 30–34 years, as was previously reported [7,26,35]. Increased sexual permissiveness and early sexual debut, multiple sexual partners, teenage pregnancy, increased HIV prevalence, sexually-transmitted infection, increased prevalence of adenocarcinoma, and poor access to healthcare and screening facilities may be partly responsible for the initial rise in mortality among young women [7,14,26,27,35,36,37]. However, the roll-out of ART in 2004 and other global HIV-control initiatives may reduce mortality among young HIV-positive patients [7,29].

### 4.3. Cohort Effect of Cervical Cancer Mortality in South Africa

We reported that the cohort RR of cervical cancer mortality gradually increased among early birth cohorts and doubled from 1924 to 1973 (RR: 0.64–1.19). It then rapidly increased to more than thrice the original RR among recent cohorts from between 1974 and 2003 (RR: 1.36–3.04). In contrast, the RR of cervical cancer mortality decreased among successive cohorts of many countries from the 1920s onward [26,28,38,39,40]. However, Korean birth cohorts experienced an increased risk from 1974 to 1988 after preceding cohorts from 1928 to 1973 experienced a decline in risk [26]. In South Africa, successive cohorts may tend to experience an increasing prevalence of: persistent high-risk HPV infection, HIV infection, ART, early sexual debut, hormonal contraceptives, cardiovascular co-morbidity, sexual permissiveness, multiple sexual partnerships, unprotected sexual intercourse, and tobacco smoking [7,12,26,28,38,39,40,41,42,43]. All the above-mentioned factors are possible due to the improved socio-economic status of the majority of women following the dismantling of the apartheid government and the commencement of a multi-racial democracy in 1994 in South Africa. It appears that the cohort effect on cervical cancer mortality trends is stronger than the period effect in South Africa as the various interventions by successive governments, especially beginning in 1994, have yet to lead to a dramatic reduction in mortality. However, the majority of countries with a decline in cervical cancer mortality appear to have strong screening and treatment programs that lead to a reduced cervical cancer mortality rate despite evidence of an increase in risky sexual behaviors among recent cohorts [1,44]. The decline in fertility rate coupled with continued exposure to Pap smear screenings and HPV vaccination of successive cohorts in South Africa can reverse the cervical cancer mortality trends in future [45].

### 4.4. Ethnic Disparity of Cervical Cancer Trends

As was previously reported, our group found that South African Blacks had a huge burden of cervical cancer with a mortality rate that was twice the rate among Coloureds and about four times the rate among Indians/Asians and Whites [7]. Furthermore, Blacks (16.7 vs. 23.9 per 100,000 MIR: 0.7), Coloureds (8.5 vs. 13.9 per 100,000 women, MIR: 0.6), and Indians/Asians (7.1 vs. 9.8 per 100,000 women MIR: 0.7) had higher cervical cancer MIRs when compared to Whites (3.9 vs. 14.7 per 100,000 women, MIR: 0.3). The high MIR among the Indian/Asian groups contrasts with the previous report [7]. The favourable survival rate among Whites may be related to early diagnosis and access to optimum oncological care.

The rapid net drift (4.3% per annum) and increased period RR of cervical cancer mortality among the Black population may be due to poor cervical cancer screening practices, poor awareness, late presentation, low socio-economic status, inadequate access to healthcare, and strong cohort risk factors [7,34,46,47,48,49] However, the observed increased mortality rate of 3.3% and increased period RR among Whites is surprising as, after the introduction of Pap smear screenings, Whites had previously exhibited reduced trends since the year 1960 [46]. Furthermore, Whites generally have a high Pap smear-uptake, high socio-economic status, and can afford optimum oncological care in private hospitals that should improve their outcome [47,48,49]. Indeed, the cervical cancer screening rate among Blacks, Indians/Asians, Coloureds, and Whites was reported as 40.1%, 77.8%, 82.9%, and 90.8%, respectively, among women older than 30 years during a South African national survey in 2012 [49].

The negative net drift and reduced period RR among Indians/Asians and Coloreds may possibly be due to a considerable uptake in Pap smear screenings. The trends in theIndian/Asian group have been decreasing since 1974 because of earlier exposure to Pap smears and an increased awareness [46]. Moreover, national free distribution of ART in 2004 may be responsible for a drop in the cervical cancer mortality rate among Blacks and Coloureds between 2005 and 2008 [7] (Table 2).

Successive cohorts of both Black and White South Africans experienced increased RRs of cervical cancer mortality, possibly because of increasing sexual permissiveness, multiple sexual partnerships, early age at sexual debut, and use of oral contraceptive pills [28,46,50]. Furthermore, increased prevalence of HIV infection, a young age at first pregnancy, and teenage pregnancy are other possible reasons for an increased cohort RR among Blacks [12,51]. On the other hand, successive White cohorts may experience increased tobacco-smoking rates [52,53]. Nonetheless, the increasing screening prevalence among successive cohorts should reduce the cohort RR. Successive birth cohorts of Indians/Asians exhibited a declining RR of cervical cancer mortality, possibly because of cultural restrictions towards risky sexual behavior, low fertility rate, low smoking rate, high socio-economic status, and good health-seeking behaviours among them [48,50,52,53].

The cervical cancer mortality RR among Coloureds declined for those born between 1924 and 1978, possibly because of low fertility rates and less-risky sexual behaviours [50]. However, the cohort RR increased among the recent cohort from 1979 to 1999. This may be partly due to changes in their social structure, with an associated increased prevalence of tobacco smoking, risky sexual behavior, teenage pregnancy, and increased HIV status [7,42,50,51,53].

### 4.5. Strength and Limitation

A strength of this study is that we have utilized high-quality, nationally representative and comprehensive mortality data. Furthermore, the triangulation of the results of both joinpoint and A–P–C modelling have made the conclusion of this study robust and useful in guiding the control of cervical cancer in South Africa and other African countries [17,19].

A limitation of this study was the missing information on the stage and histological types of the cervical cancers [7,54]. Although it is mandatory to report all deaths in South Africa to the department of home affairs before burial, a few deaths may not have been reported and this may suggest that the actual cervical cancer mortality rates may be higher than what our group reported [55]. There may be differential reporting of cervical cancer mortality based on ethnicity. However, a law proscribing burial without reporting to the authorities may reduce such differences.

## 5. Conclusions

In conclusion, we found that a significant age–period–cohort effect was observed for cervical cancer mortality trends. There was a net drift in cervical cancer mortality rates of about 1.5% per annum from 1999 to 2018, largely driven by a rapid rise in deaths among young women (in the last 10 years of the study) and worsening risks of cervical cancer mortality among recent cohorts. The period effect from screening and an expansion of healthcare services after the establishment of a multi-racial democracy in 1994 led to minimal reduction in cervical cancer deaths, especially among women aged 50–59 years. The cervical cancer mortality rate among Blacks was about twice the rate among Coloureds and about four times the rate among Whites and Indians/Asians. Each of the four ethnic groups had differential trends and burdens due to peculiar socio-economic, cultural, and screening behaviours; access to optimum care; awareness; and sexual and reproductive behaviours. The identified disparities and trends are very useful for designing targeted intervention.

### Brief Policy Implications

In order to reduce the burden of cervical cancer, the South African government initiated the national cervical cancer control programme in 2002 and 2017 [10]. Based on the results of our study, we recommend that screening for cervical cancer should begin early at 20–25 years among sexually active women, as our results suggests strong/highest risks among young cohorts [10]. Additionally, the nation-wide HPV vaccination of 9-year-old schoolgirls that began in 2014 should be continued to reduce the risk of cervical cancer among young women [56].

Efforts geared towards primary prevention that targets all the known risk factors of cervical cancer such as encouraging safe sexual practice (especially the use of condoms) and reproductive behaviours should be intensified as cohort effect impacts cervical cancer mortality trends. Ethnicity-specific interventions should be encouraged after further research as there was disparity observed in ethnic cervical cancer deaths. Furthermore, oncological facilities for the prompt treatment of all stages of cervical cancer should be provided in all the provinces of South Africa to reduce the burden of death from the disease. This will entail investment in training of all cadres of oncological staff and provision of surgical, radiation, and palliative services.

## Figures and Tables

**Table 1:** Trends in the mortality rates and mean age at death of cervical cancer in South Africa (1999–2018).

Year	*N* = 59,190			
	Cervix Mortality (% of Gynae & Breast) ≥15 Years	Age Mean ± SD	CMR	ASMR
1999	2134 (46.22)	56.09 ± 14.25	13.88	11.7
2000	2270 (47.73)	55.54 ± 14.29	14.38	12.02
2001	2303 (44.95)	55.94 ± 13.85	14.23	12.05
2002	2378 (45.70)	55.94 ± 14.08	14.49	12.14
2003	2553 (47.62)	56.26 ± 14.40	15.02	12.49
2004	2569 (43.71)	56.34 ± 14.21	15.48	13.89
2005	2700 (44.73)	56.49 ± 14.27	16.62	13.7
2006	2725 (44.81)	55.60 ± 14.40	16.54	13.51
2007	2603 (41.99)	55.87 ± 14.56	15.58	12.6
2008	2651 (42.40)	56.64 ± 14.75	15.15	12.48
2009	2910 (43.45)	56.16 ± 14.88	16.39	13.38
2010	2877 (42.46)	56.53 ± 14.92	16.01	13.21
2011	3031 (42.71)	56.12 ± 14.88	16.63	13.22
2012	3150 (43.84)	55.55 ± 14.87	17.07	13.51
2013	3239 (42.89)	55.76 ± 14.89	16.64	12.74
2014	3604 (44.00)	55.45 ± 14.94	18.37	13.72
2015	3708 (44.00)	55.44 ± 14.91	18.70	14.35
2016	3852 (43.28)	55.13 ± 14.72	19.06	14.6
2017	3940 (43.36)	55.38 ± 14.66	19.16	14.49
2018	3993 (42.65)	55.17 ± 14.78	18.98	14.08

CMR: Crude mortality rate per 100,000 women; ASMR: age-standardised mortality rate per 100,000 women.

**Table 2:** Joinpoint regression estimates of the trends in age-standardised mortality rates for cervical cancer in South Africa (1999–2018).

Cancer Type	Trends	Year Period	APC	95% CI		*p*-Value	Comment
Overall ASMR	1	1999–2005	2.9 *	1.1	4.7	<0.001	Significant increase
	2	2005–2008	−2.4	−11.9	8.0	0.6	Non-significant decrease
	3	2008–2018	1.4 *	0.6	2.1	<0.001	Significant increase
	Full Range	1999–2018	0.9 *	0.6	1.2	<0.001	Significant increase
Black	1	1999–2002	−1.8	−8.9	5.8	0.6	Non-significant decrease
	2	2002–2005	6.6	−7.4	22.9	0.3	Non-significant increase
	3	2005–2008	−2.1	−15.0	12.7	0.7	Non-significant decrease
	4	2008–2018	3.5 *	2.5	4.6	<0.001	Significant increase
	Full Range	1999–2018	2.4 *	1.9	2.9	<0.001	Significant increase
Indian/Asian	1	1999–2004	−8.8 *	−14.7	−2.4	<0.001	Significant decrease
	2	2004–2018	0.6	−1.0	2.3	0.4	Non-significant increase
	Full Range	1999–2018	−1.4 *	−2.6	−0.2	<0.001	Significant decrease
Coloured							
	1	1999–2005	1.1	−3.0	5.4	0.6	Non-significant increase
	2	2005–2008	−8.1	−29.7	20.1	0.5	Non-significant decrease
	3	2008–2016	2.8	−0.7	6.5	0.1	Non-significant increase
	4	2016–2018	−13.5	−34.8	14.8	0.3	Non-significant decrease
	Full Range	1999–2018	−0.7	−1.5	0.1	0.1	Non-significant decrease
White							
	1	1999–2010	0.1	−1.2	1.4	0.8	Stable
	2	2010–2018	7.8 *	5.9	9.7	<0.001	Significant increase
	Full Range	1999–2018	3.3 *	2.3	4.4	<0.001	Significant increase

* Statistically significant at *p*-value < 0.05.

## Data Availability

Data may be obtained from Statistics South Africa.

## Supplementary Materials

The following supporting information can be downloaded at: https://www.mdpi.com/article/10.3390/cancers14246256/s1, Figure S1: Joinpoint regression of the age-specific death rates of cervical cancer among Whites in South Africa (1999–2018); Figure S2: Joinpoint regression of the age-specific death rates of cervical cancer among Coloureds in South Africa (1999–2018); Figure S3: Joinpoint regression of the age-specific death rates of cervical cancer among Indians/Asians in South Africa (1999–2018); Figure S4: Joinpoint regression of the age-specific death rates of cervical cancer among Blacks in South Africa (1999–2018); Figure S5: Age, period, and cohort effects of national (A); and ethnic trends of cervical cancer mortality in South Africa (1999–2018); Blacks (B), Whites (C), Coloureds (D), and Indians/Asians (E) (Local drift, fitted temporal trends, longitudinal age curve, cross sectional age curve, period effect, and cohort effect were depicted for each ethnic groups. Table S1: Trends in the mortality rate and mean age at death from cervical cancer among ethnic groups in South Africa (1999–2018); Table S2: Joinpoint estimates of the national and ethnic trends in age-specific mortality rates for cervical cancers in South Africa (1999–2018); Table S3: The overall and ethnic age-specific mortality rates for cervical cancers in South Africa (2018); Table S4: The age–period–cohort effect estimates of trends in cervical cancer mortality in South Africa (1999–2018); Table S5: Age–period–cohort effect estimates of ethnic trends in cervical cancer mortality by ethnic group in South Africa (1999–2018); Table S6: Wald Chi-square test for estimable functions of the age–period–cohort model in the overall and ethnic trends of mortality for cervical cancers in South Africa (1999–2018).

## References

[1] Sung H., Ferlay J., Siegel R.L., Laversanne M., Soerjomataram I., Jemal A., Bray F. (2021). Global Cancer Statistics 2020: GLOBOCAN Estimates of Incidence and Mortality Worldwide for 36 Cancers in 185 Countries. CA Cancer J. Clin..

[2] Hull R., Mbele M., Makhafola T., Hicks C., Wang S.M., Reis R.U.I.M., Mehrotra R., Kwitshana Z.M., Kibiki G., Bates D.O. (2020). Cervical cancer in low and middle—Income countries. Review.

[3] Torre L.A., Islami F., Siegel R.L., Ward E.M., Jemal A. (2017). Global cancer in women: Burden and trends. Cancer Epidemiol. Biomarkers Prev..

[4] International Collaboration of Epidemiological Studies of Cervical Cancer (2009). Cervical Carcinoma and Sexual Behavior: Collaborative Reanalysis of Individual Data on 15,461 Women with Cervical Carcinoma and 29,164 Women without Cervical Carcinoma from 21 Epidemiological Studies. Cancer Epidemiol. Biomarkers Prev..

[5] Beral V., Colin D., Franceschi S., Green J., La Vecchia C., Peto J., Randi G., Herrero R., Hildesheim A., Vecchia C.L. (2006). Cervical carcinoma and reproductive factors: Collaborative reanalysis of individual data on 16,563 women with cervical carcinoma and 33,542 women without cervical carcinoma from 25 epidemiological studies. Int. J. Cancer.

[6] Urban M., Banks E., Egger S., Canfell K., O’Connell D., Beral V., Sitas F. (2012). Injectable and oral contraceptive use and cancers of the breast, cervix, ovary, and endometrium in black south african women: Case-control study. PLoS Med..

[7] Olorunfemi G., Ndlovu N., Masukume G., Chikandiwa A., Pisa P.T., Singh E. (2018). Temporal trends in the epidemiology of cervical cancer in South Africa (1994–2012). Int. J. Cancer.

[8] Cooper D., Morroni C., Orner P., Moodley J., Harries J., Cullingworth L., Hoffman M. (2004). Ten years of democracy in South Africa: Documenting transformation in reproductive health policy and status. Reprod. Health Matters.

[9] Cooper D., Harries J., Moodley J., Constant D., Hodes R., Mathews C., Morroni C., Hoffman M. (2016). Coming of age? Women’s sexual and reproductive health after twenty-one years of democracy in South Africa. Reprod. Health Matters.

[10] South African National Department of Health (2017). Cervical Cancer Prevention and Control Policy.

[11] Mabaso M., Makola L., Naidoo I., Mlangeni L.L., Jooste S., Simbayi L. (2019). HIV prevalence in South Africa through gender and racial lenses: Results from the 2012 population-based national household survey. Int. J. Equity Health.

[12] Shisana O., Rehle T., Simbayi L.C., Zuma K., Jooste S., Zungu N., Labadarios D., Onoya D. (2012). South African National HIV Prevalence, Incidence and Behaviour Survey, 2012. Hum. Sci. Res. Counc..

[13] Mbulawa Z.Z.A., Van Schalkwyk C., Hu N.C., Meiring T.L., Barnabas S., Dabee S., Jaspan H., Kriek J.M., Jaumdally S.Z., Muller E. (2018). High human papillomavirus (HPV) prevalence in South African adolescents and young women encourages expanded HPV vaccination campaigns. PLoS ONE.

[14] Olorunfemi G. (2017). Trends and Determinants of the Incidence and Mortality of Cervical Cancer in South Africa (1994–2012). Master’s Thesis.

[15] Bhatla N., Aoki D., Nand D., Rengaswamy S. (2021). Cancer of the cervix uteri: 2021 update. Int. J. Gynecol. Obstet..

[16] Bell A., Bell A. (2020). Annals of Human Biology Age period cohort analysis: A review of what we should and shouldn’t do Age period cohort analysis: A review of what we should and shouldn’t do. Ann. Hum. Biol..

[17] Nkengasong J., Gudo E., Macicame I., Maunze X., Amouzou A., Banke K., Dowell S., Jani I. (2020). Improving birth and death data for African decision making. Lancet Glob. Health.

[18] Statistics South Africa (2015). Mid-Year Population Estimates 2015.

[19] Joubert J., Rao C., Bradshaw D., Vos T., Lopez A.D. (2013). Evaluating the Quality of National Mortality Statistics from Civil Registration in South Africa, 1997–2007. PLoS ONE.

[20] Rosenberg P.S., Check D.P., Anderson W.F. (2014). A Web Tool for Age—Period—Cohort Analysis of Cancer Incidence and Mortality Rates. Cancer Epidemiol. Biomarkers Prev..

[21] Bray F., Ferlay J., Soerjomataram I., Siegel R.L., Torre L.A., Jemal A. (2018). Global cancer statistics 2018: GLOBOCAN estimates of incidence and mortality worldwide for 36 cancers in 185 countries. CA Cancer J. Clin..

[22] National Cancer Registry (2021). Cancer in South Africa 2018. https://www.nicd.ac.za/centres/national-cancer-registry/.

[23] Zhang X., Zeng Q., Cai W., Ruan W. (2021). Trends of cervical cancer at global, regional, and national level: Data from the Global Burden of Disease study 2019. BMC Public Health.

[24] Akinyemiju T.F., McDonald J.A., Lantz P.M. (2015). Health care access dimensions and cervical cancer screening in South Africa: Analysis of the world health survey. BMC Public Health.

[25] Guo M., Xu J., Du J. (2021). Trends in cervical cancer mortality in China from 1989 to 2018: An age-period-cohort study and Joinpoint analysis. BMC Public Health.

[26] Moon E.K., Oh C.M., Won Y.J., Lee J.K., Jung K.W., Cho H., Jun J.K., Lim M.C., Ki M. (2017). Trends and age-period-cohort effects on the incidence and mortality rate of cervical cancer in Korea. Cancer Res. Treat..

[27] Piechocki M., Koziołek W., Sroka D., Matrejek A., Miziołek P., Saiuk N., Sledzik M., Jaworska A., Bereza K., Pluta E. (2022). Trends in Incidence and Mortality of Gynecological and Breast Cancers in Poland (1980–2018). Clin. Epidemiol..

[28] Wang J., Bai Z., Gao X., Zhang N., Wang Z. (2021). The Effects of Age, Period, and Cohort on the Mortality of Cervical Cancer in Three High-Income Countries: Canada, Korea, and Italy. Biomed Res. Int..

[29] Pillay-van Wyk V., Msemburi W., Laubscher R., Dorrington R.E., Groenewald P., Glass T., Nojilana B., Joubert J.D., Matzopoulos R., Prinsloo M. (2016). Mortality trends and differentials in South Africa from 1997 to 2012: Second National Burden of Disease Study. Lancet Glob. Health.

[30] Mbulaiteye S.M., Parkin D.M., Rabkin C.S. (2003). Epidemiology of AIDS-related malignancies. Hematol. Oncol. Clin. N. Am..

[31] Simelela N.P., Venter W.D.F. (2014). A brief history of South Africa’s response to AIDS. S. Afr. Med. J..

[32] Reniers G., Blom S., Calvert C., Martin-Onraet A., Herbst A.J., Eaton J.W., Bor J., Slaymaker E., Li Z.R., Clark S.J. (2017). Trends in the burden of HIV mortality after roll-out of antiretroviral therapy in KwaZulu-Natal, South Africa: An observational community cohort study. Lancet HIV.

[33] Sartorius B., Kahn K., Collinson M.A., Sartorius K., Tollman S.M. (2013). Dying in their prime: Determinants and space-time risk of adult mortality in rural South Africa. Geospat. Health.

[34] Mayosi B.M., Lawn J.E., van Niekerk A., Bradshaw D., Abdool Karim S.S., Coovadia H.M. (2012). Health in South Africa: Changes and challenges since 2009. Lancet.

[35] Tanaka S., Palmer M., Katanoda K. (2022). Trends in cervical cancer incidence and mortality of young and middle adults in Japan. Cancer Sci..

[36] Teixeira J.C., Santos D.Z., Campos C.S., Vale D.B., Bragança J.F., Zeferino L.C. (2021). Cervical cancer in women under 25 years of age and outside the screening age: Diagnosis profile and long-term outcomes. Int. J. Gynecol. Obstet..

[37] Gravdal B.H., Lönnberg S., Skare G.B., Sulo G., Bjørge T. (2021). Cervical cancer in women under 30 years of age in Norway: A population-based cohort study. BMC Womens. Health.

[38] Huang Z., Zheng Y., Wen W., Wu C., Bao P., Wang C., Zhong W., Gao Y.T., Jin F., Xiang Y.B. (2016). Incidence and mortality of gynaecological cancers: Secular trends in urban Shanghai, China over 40 years. Eur. J. Cancer.

[39] Sathishkumar K., Vinodh N., Badwe R.A., Deo S.V.S., Manoharan N., Malik R., Panse N.S., Ramesh C., Shrivastava A., Swaminathan R. (2021). Trends in breast and cervical cancer in India under National Cancer Registry Programme: An Age-Period-Cohort analysis. Cancer Epidemiol..

[40] Wang Z., Guo E., Yang B., Xiao R., Lu F., You L., Chen G. (2021). Trends and age-period-cohort effects on mortality of the three major gynecologic cancers in China from 1990 to 2019: Cervical, ovarian and uterine cancer. Gynecol. Oncol..

[41] Bakilana A. (2005). Age at sexual debut in South Africa. Afr. J. AIDS Res..

[42] Reddy P., Zuma K., Shisana O., Kim J., Sewpaul R. (2015). Prevalence of tobacco use among adults in South Africa: Results from the first South African National Health and Nutrition Examination Survey. S. Afr. Med. J..

[43] Bruni L., Albero G., Serrano B., Mena M., Collado J., Gómez D., Muñoz J., Bosch F., de Sanjosé S. (2016). Human Papillomavirus and Related Diseases Report.

[44] Patel A., Galaal K., Burnley C., Faulkner K., Martin-Hirsch P., Bland M.J., Leeson S., Beer H., Paranjothy S., Sasieni P. (2012). Cervical cancer incidence in young women: A historical and geographic controlled UK regional population study. Br. J. Cancer.

[45] Tathiah N., Naidoo M., Moodley I. (2015). Human papillomavirus (HPV) vaccination of adolescents in the South African private health sector: Lessons from the HPV demonstration project in KwaZulu-Natal. S. Afr. Med. J..

[46] Bailie R., Selvey C.E., Bourne D., Bradshaw D. (1996). Trends in cervical cancer mortality in South Africa. Int. J. Epidemiol..

[47] Akokuwebe M.E., Idemudia E.S., Lekulo A.M., Motlogeloa O.W. (2021). Determinants and levels of cervical Cancer screening uptake among women of reproductive age in South Africa: Evidence from South Africa Demographic and health survey data, 2016. BMC Public Health.

[48] Coovadia H., Jewkes R., Barron P., Sanders D., McIntyre D. (2009). The health and health system of South Africa: Historical roots of current public health challenges. Lancet.

[49] Phaswana-mafuya N., Peltzer K. (2018). Breast and Cervical Cancer Screening Prevalence and Associated Factors among Women in the South African General Population. Asian Pac. J. Cancer Prev..

[50] Caldwell J.C., Caldwell P. (1993). The South African fertility decline. Popul. Dev. Rev..

[51] Mkhwanazi N. (2010). Understanding teenage pregnancy in a post-apartheid South African township. Cult. Health Sex..

[52] Sitas F., Egger S., Bradshaw D., Groenewald P., Laubscher R., Kielkowski D., Peto R. (2013). Differences among the coloured, white, black, and other South African populations in smoking-attributed mortality at ages 35-74 years: A case-control study of 481,640 deaths. Lancet.

[53] Reddy P., James S., Sewpaul R., Yach D., Resnicow K., Sifunda S., Mthembu Z., Mbewu A. (2013). A decade of tobacco control: The South African case of politics, health policy, health promotion and behaviour change. S. Afr. Med. J..

[54] Statistics South Africa (2020). StatsSA Statistical Release P0302: Mid-Year Population Estimates 2020.

[55] Loney T., Nagelkerke N.J. (2014). The individualistic fallacy, ecological studies and instrumental variables: A causal interpretation. Emerg. Themes Epidemiol..

[56] Amponsah-Dacosta E., Blose N., Nkwinika V.V., Chepkurui V. (2022). Human Papillomavirus Vaccination in South Africa: Programmatic Challenges and Opportunities for Integration with Other Adolescent Health Services?. Front. Public Health.

