# Supplementary material for: Population-Based Temporal Trends and Ethnic Disparity in Cervical Cancer Mortality in South Africa (1999–2018): A Join Point and Age–Period–Cohort Regression Analyses
Source: Cancers (Basel). 2022 Dec 19;14(24):6256. doi: 10.3390/cancers14246256 (PMC9816936; doi:10.3390/cancers14246256)
Supplement: Supplementary file 1 [file cancers-14-06256-s001.zip › cancers-2011464-Supplementary Materials.pdf]

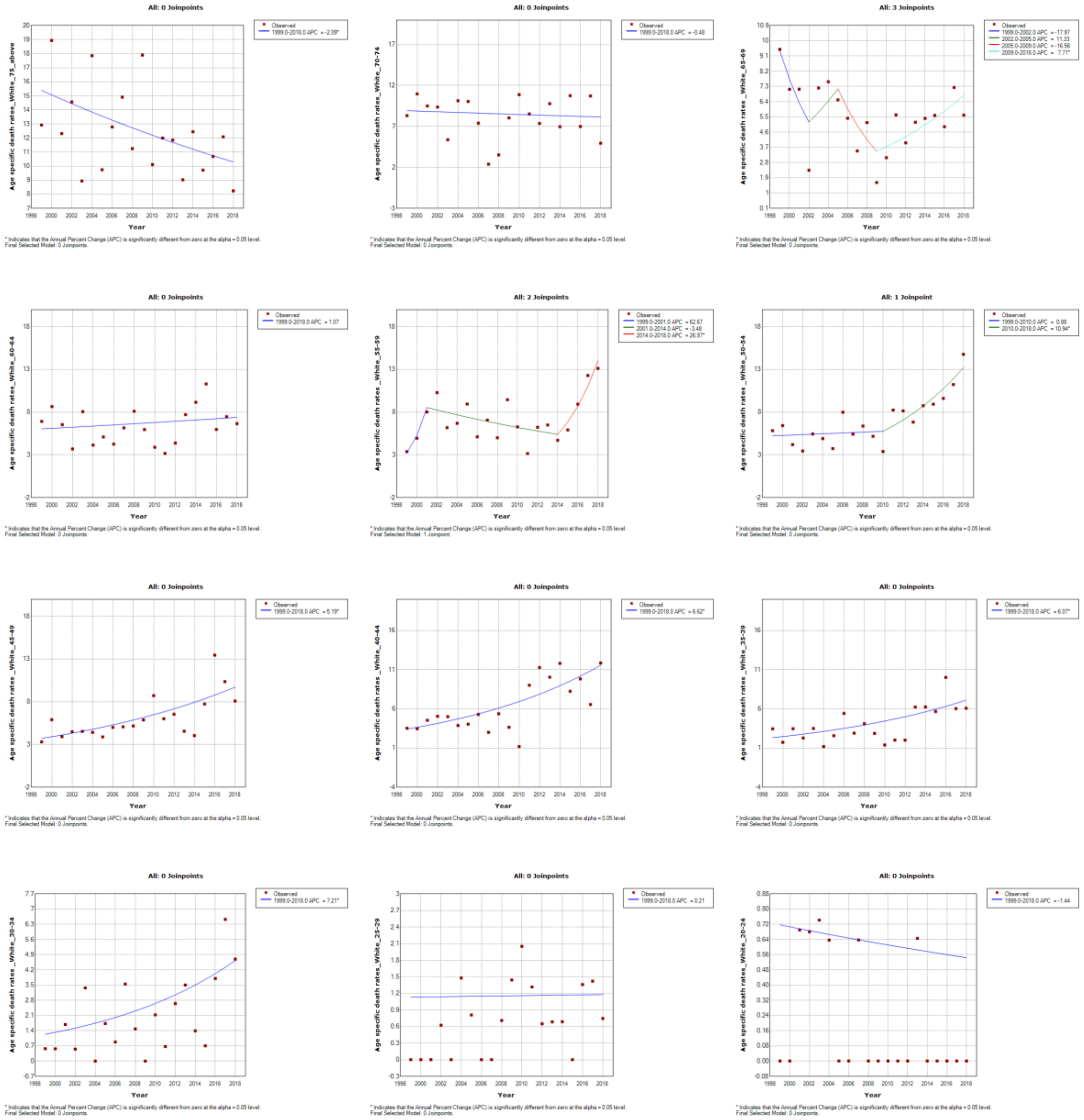

Figure S1. Join point regression of the age specific death rates of cervical cancer among Whites in South Africa (1999 – 2018)

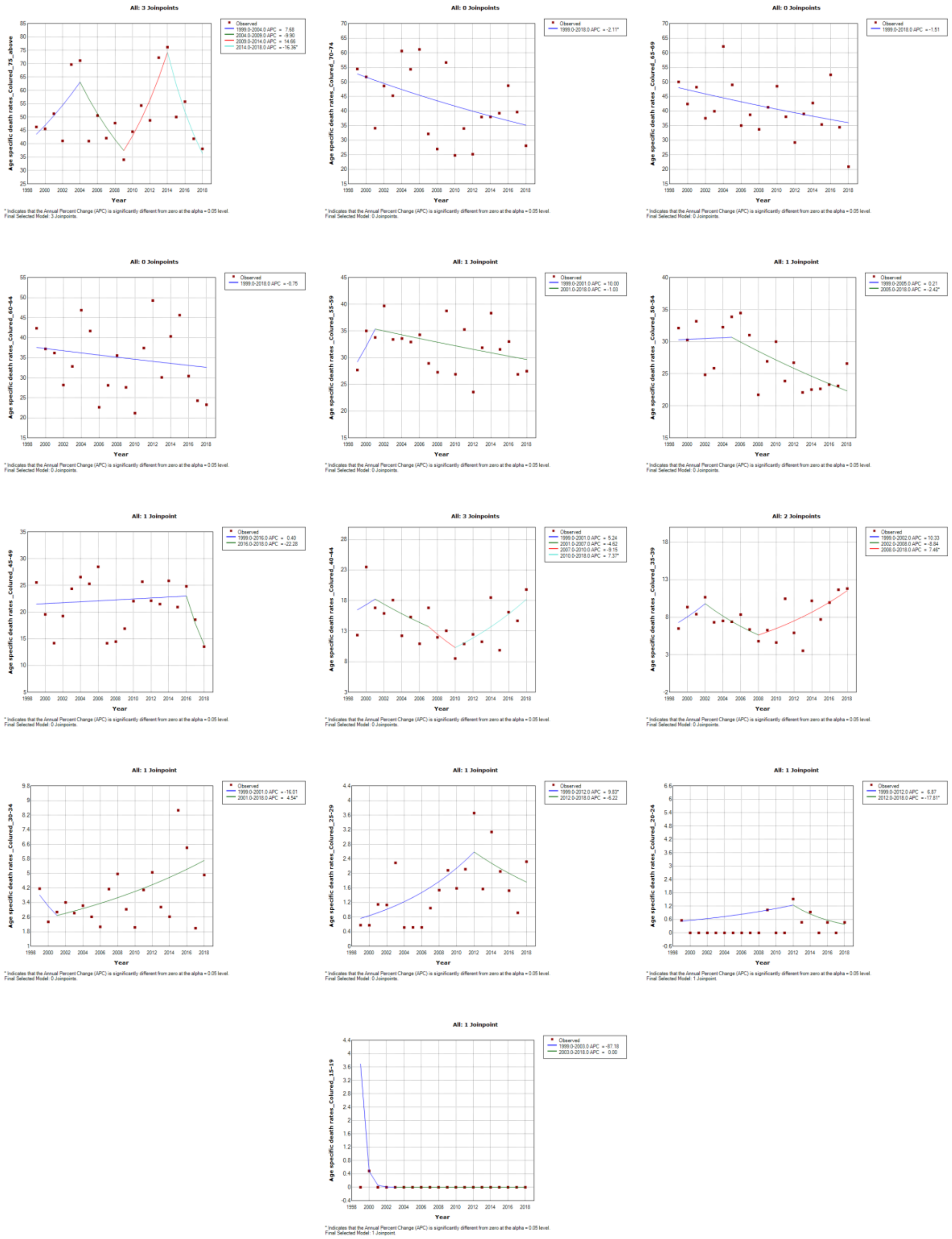

Figure S2. Join point regression of the age specific death rates of cervical cancer among Coloured in South Africa (1999 – 2018)

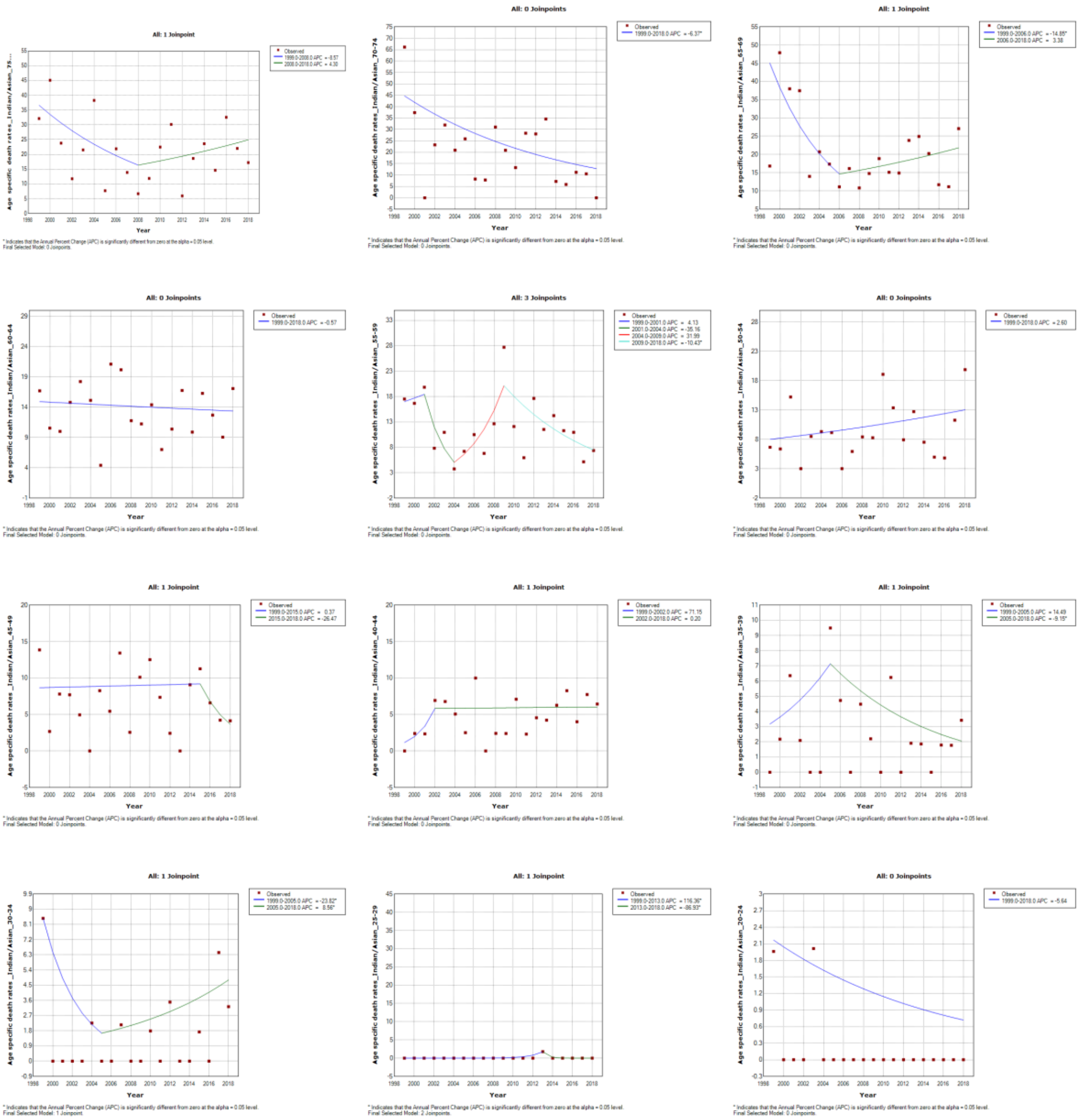

Figure S3. Join point regression of the age specific death rates of cervical cancer among Indian/Asian in South Africa (1999 – 2018)

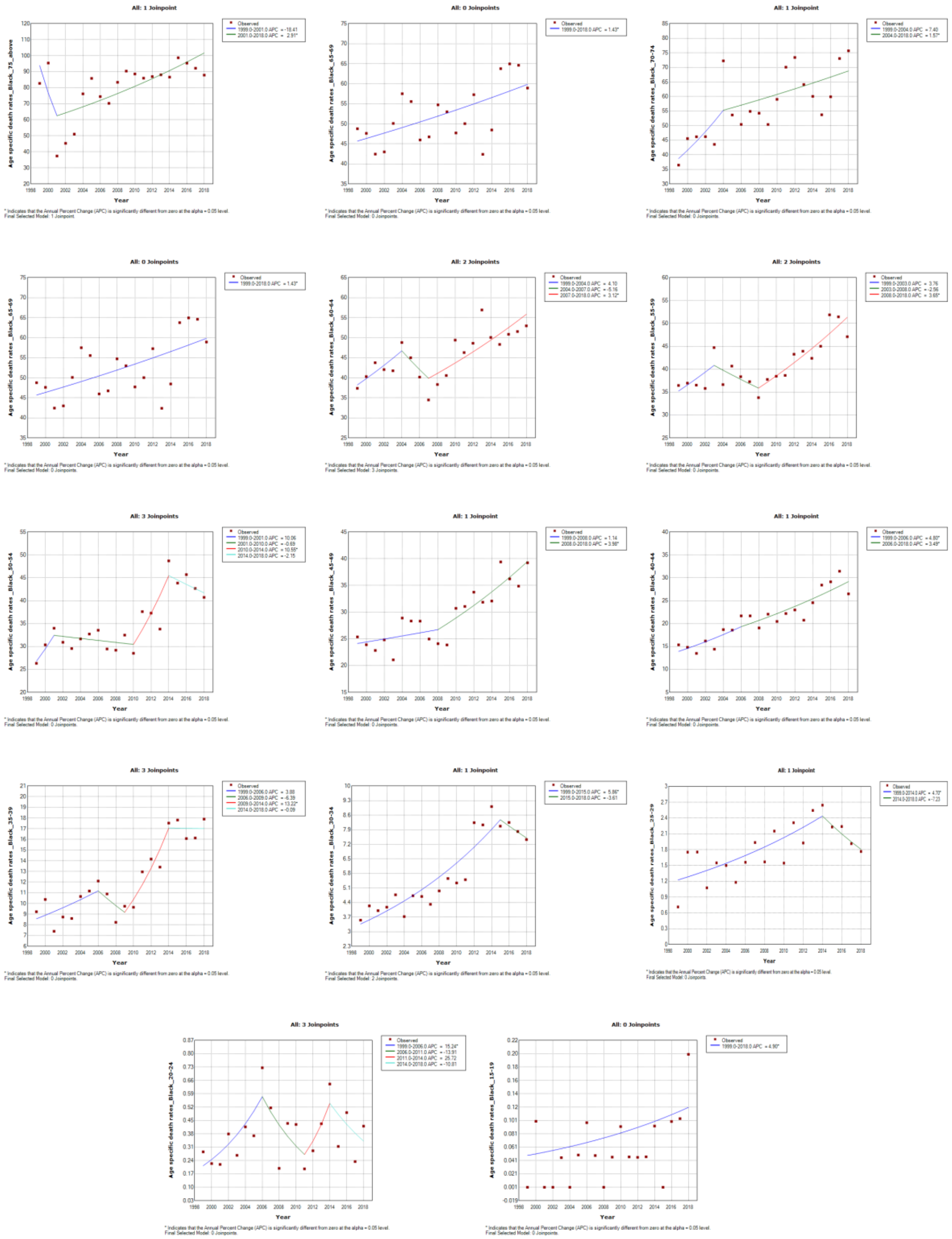

Figure S4. Join point regression of the age specific death rates of cervical cancer among Blacks in South Africa (1999 – 2018)

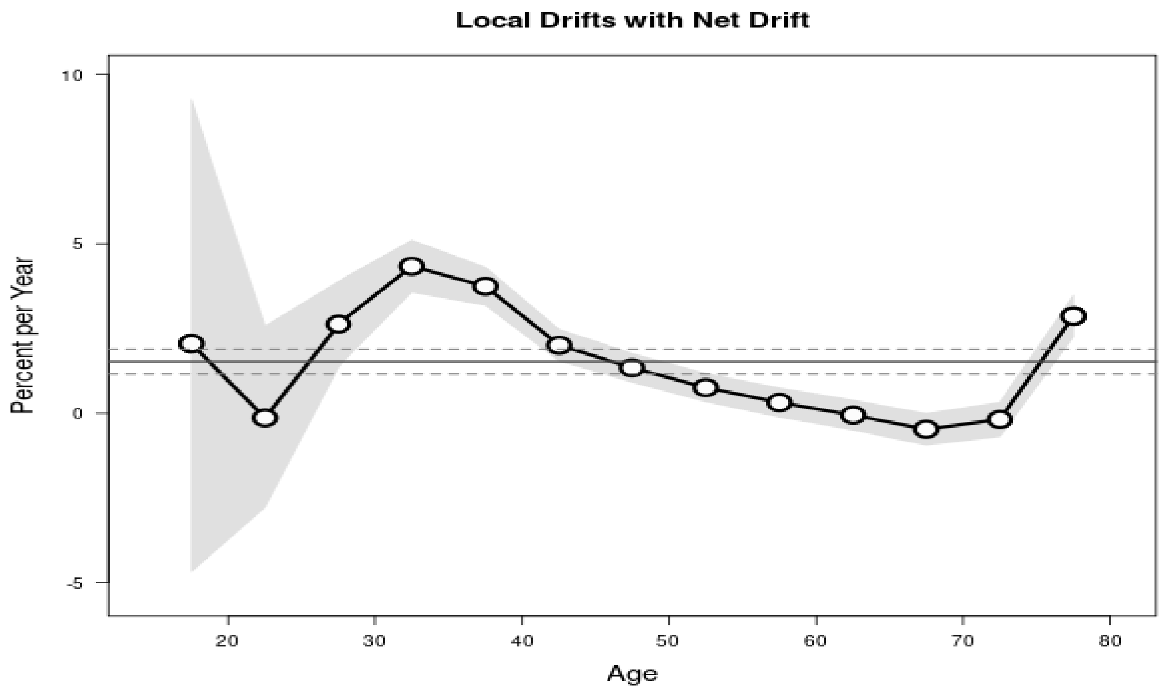
Local Drifts with Net Drift
Percent per Year
10
5
0
-5
20
30
40
50
60
70
80
Age

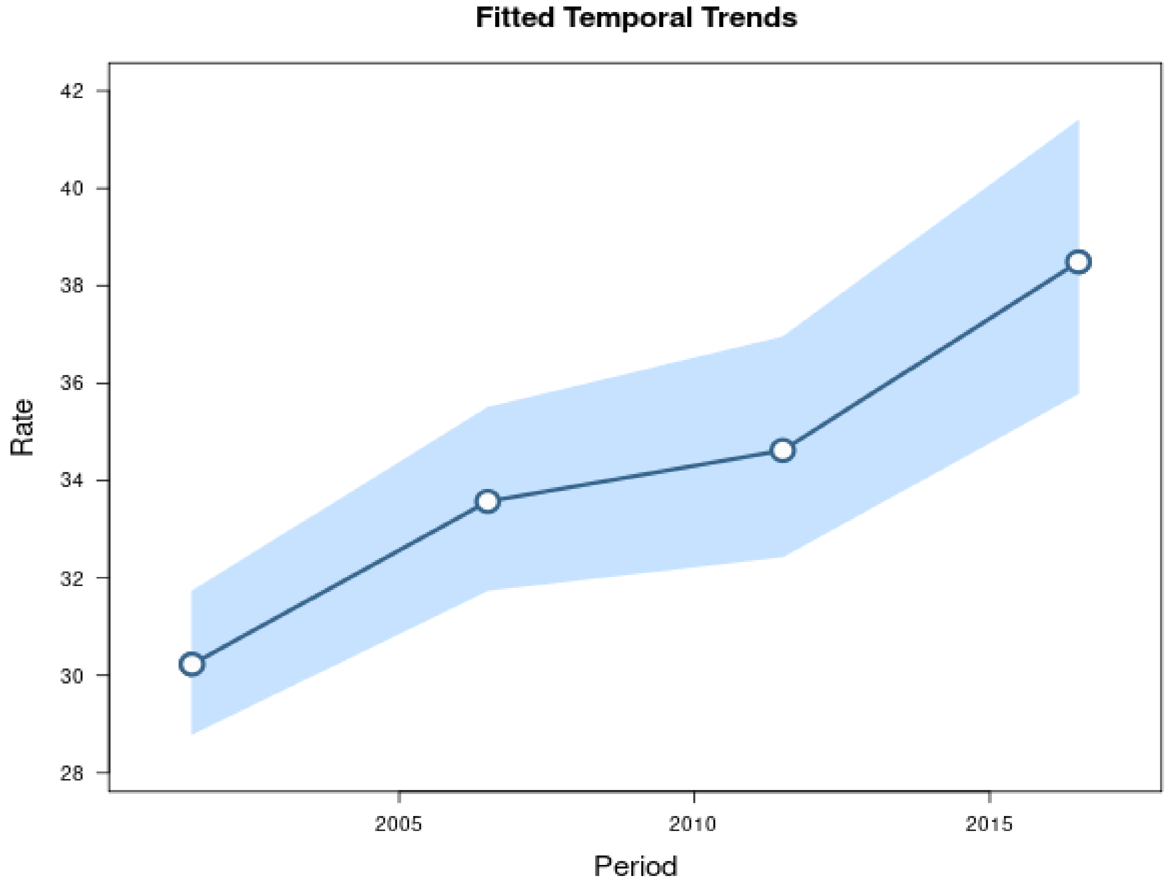
Fitted Temporal Trends
Rate
42
40
38
36
34
32
30
28
2005
2010
2015
Period

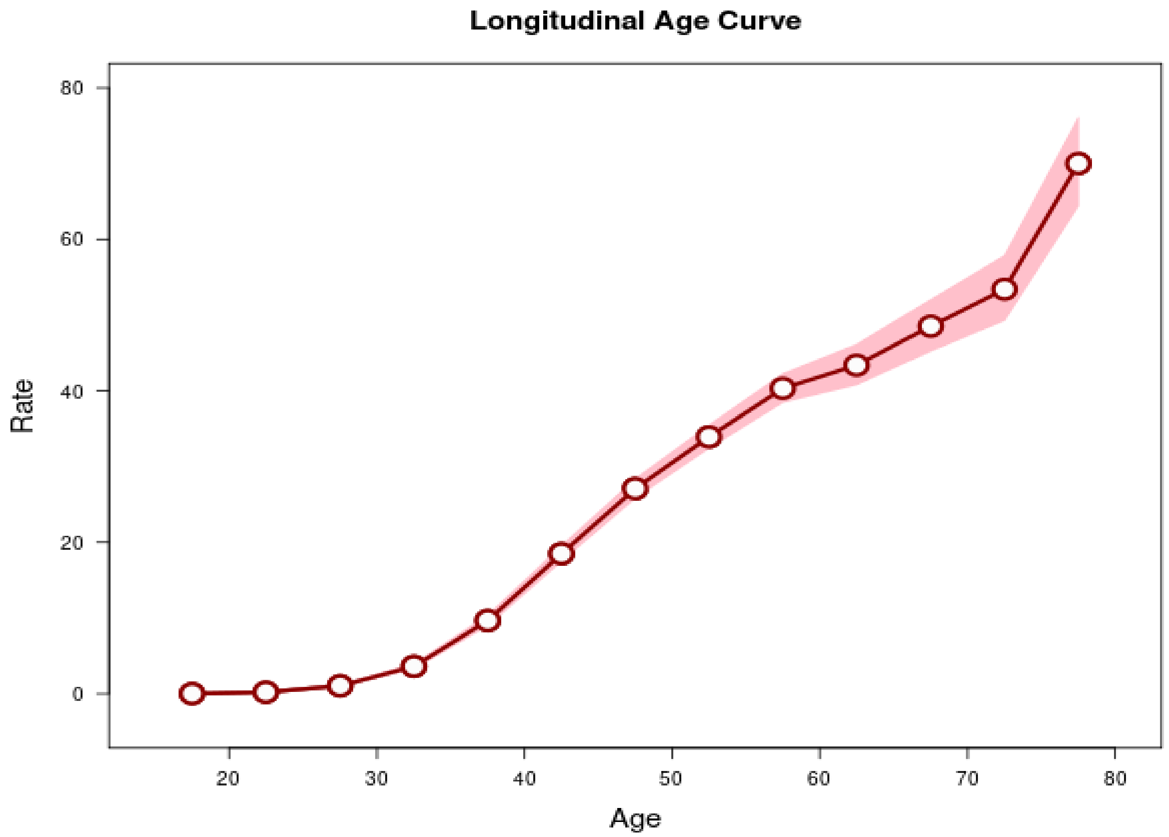
Longitudinal Age Curve
Rate
Age
0
20
40
60
80
20
30
40
50
60
70
80

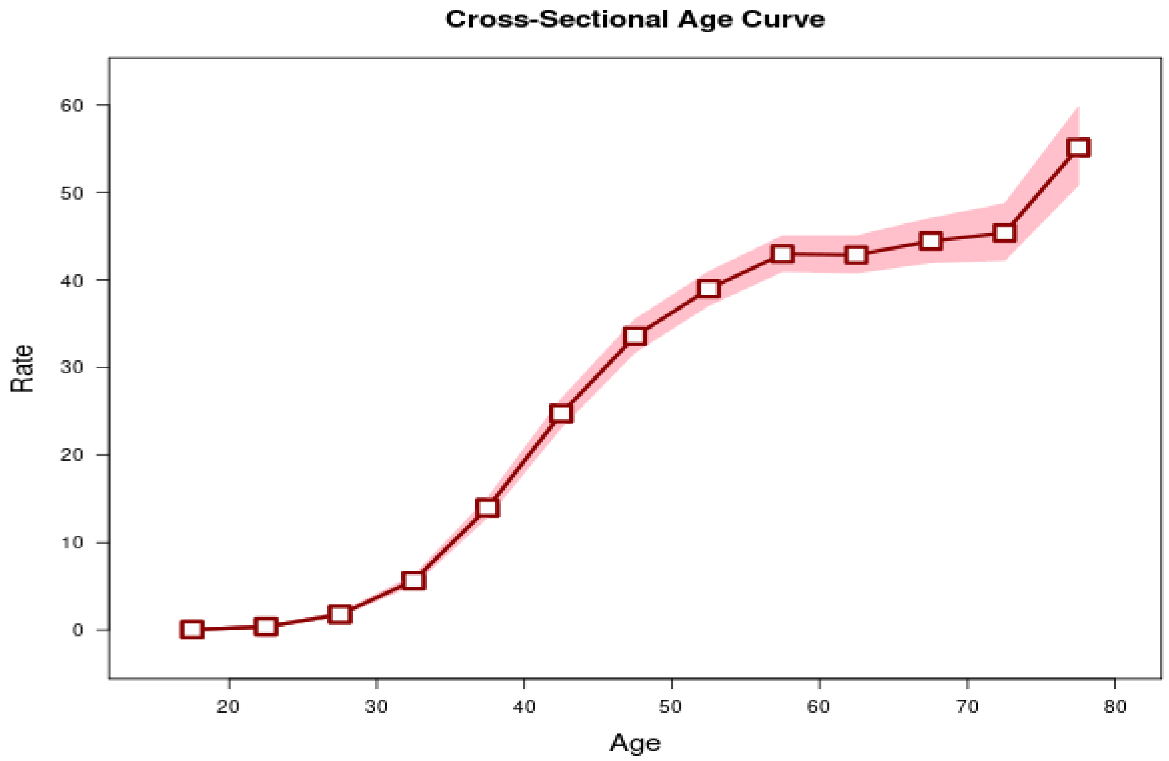
Cross-Sectional Age Curve
Rate
Age
0
10
20
30
40
50
60
20
30
40
50
60
70
80

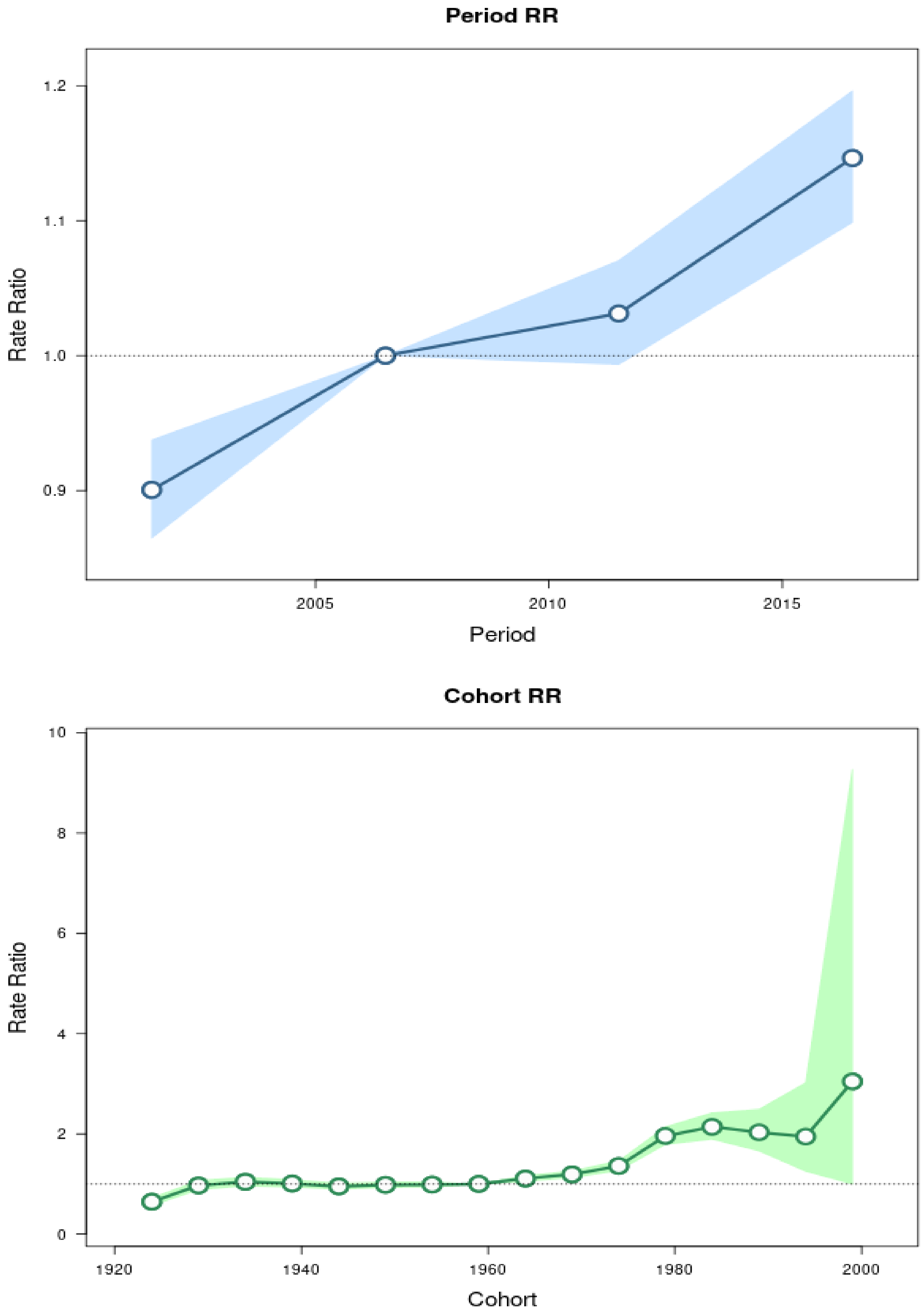
Period RR
Rate Ratio
1.2
1.1
1.0
0.9
2005
2010
2015
Period
Cohort RR
Rate Ratio
10
8
6
4
2
0
1920
1940
1960
1980
2000
Cohort

(**A**)

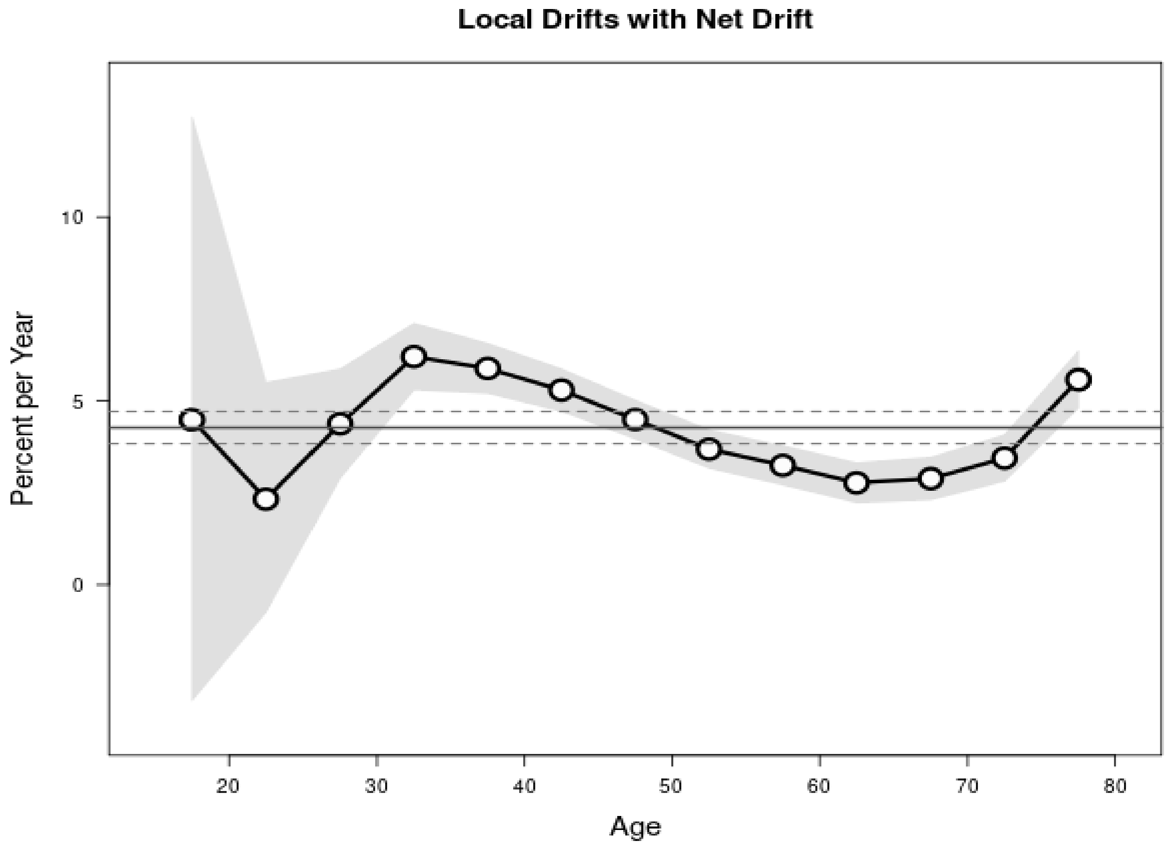
Local Drifts with Net Drift
Percent per Year
10
5
0
20
30
40
50
60
70
80
Age

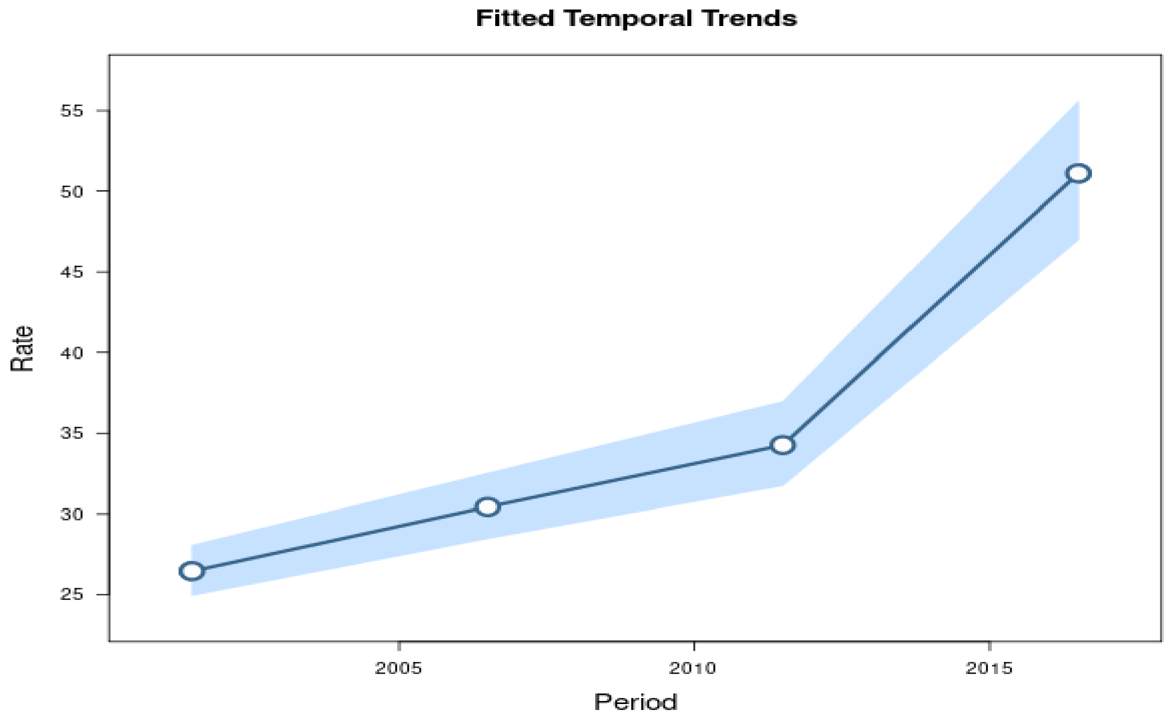
Fitted Temporal Trends
Rate
55
50
45
40
35
30
25
2005
2010
2015
Period

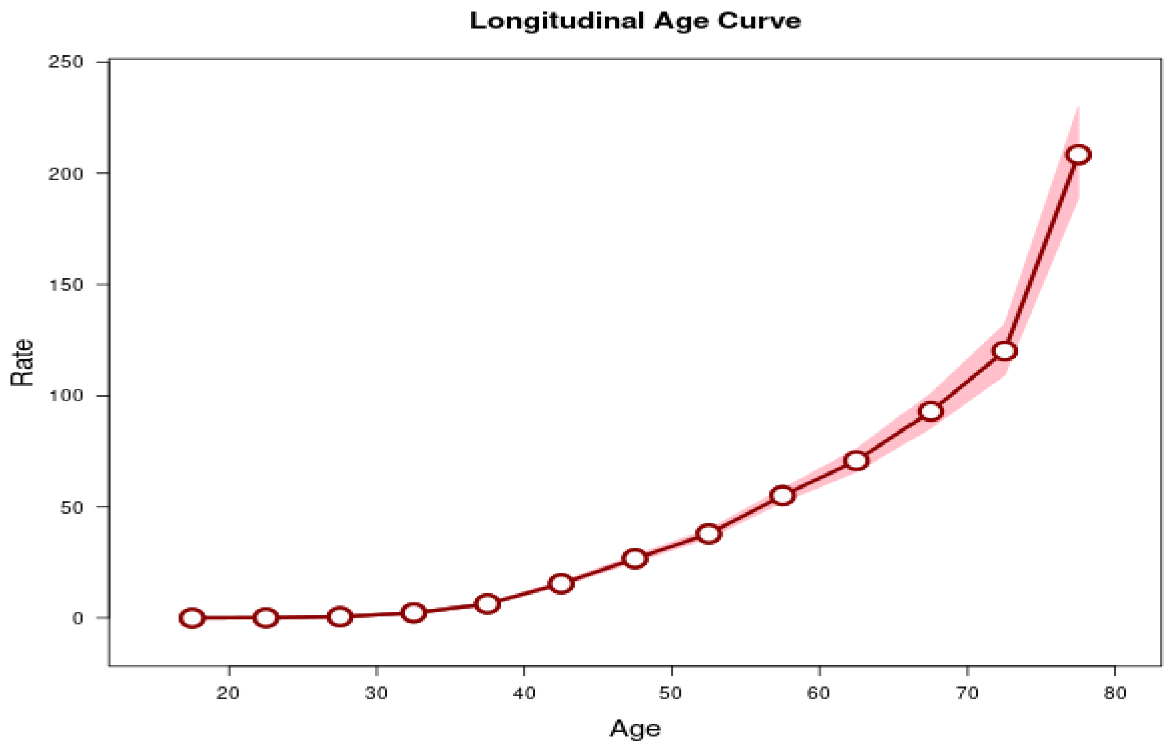
Longitudinal Age Curve
Rate
Age
0
50
100
150
200
250
20
30
40
50
60
70
80

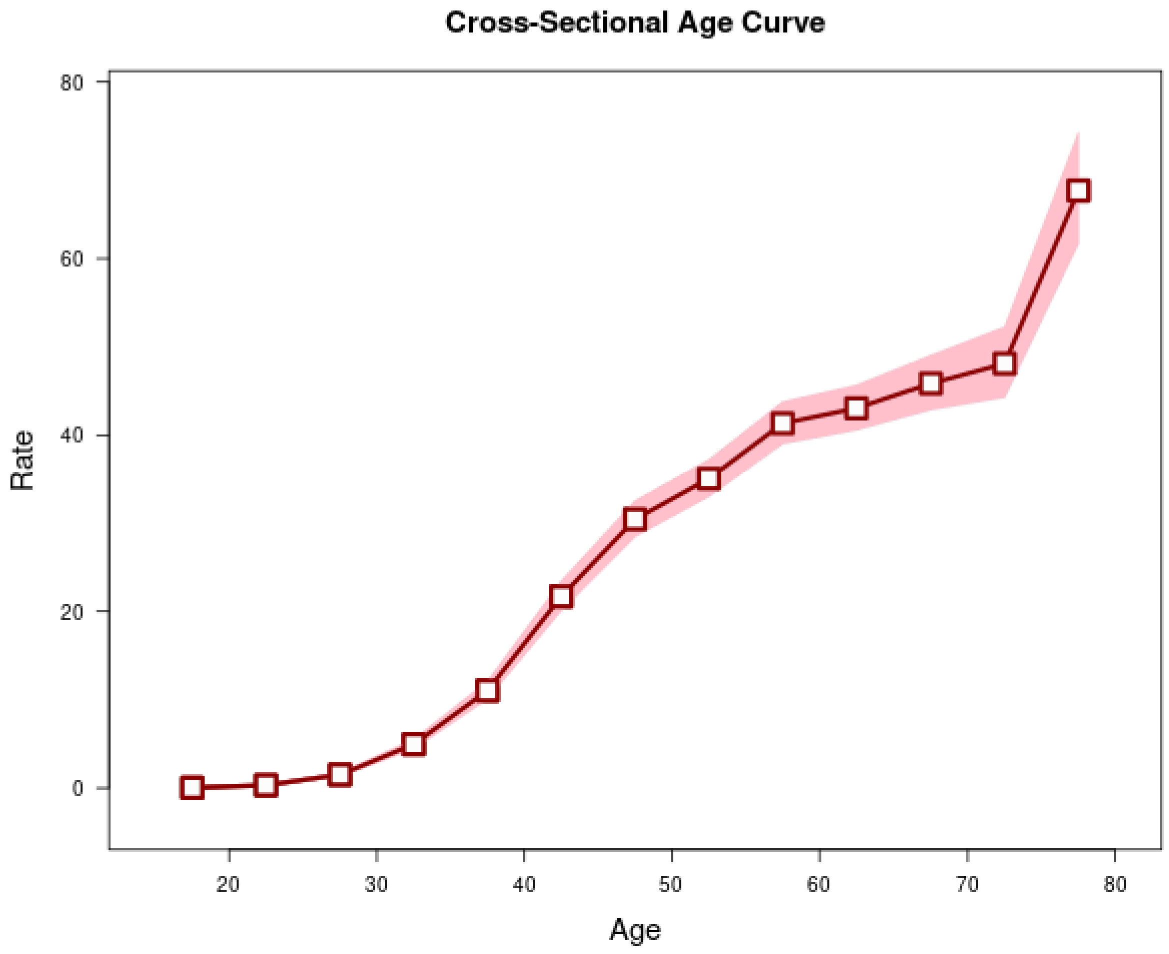
Cross-Sectional Age Curve
Rate
Age
0
20
40
60
80
20
30
40
50
60
70
80

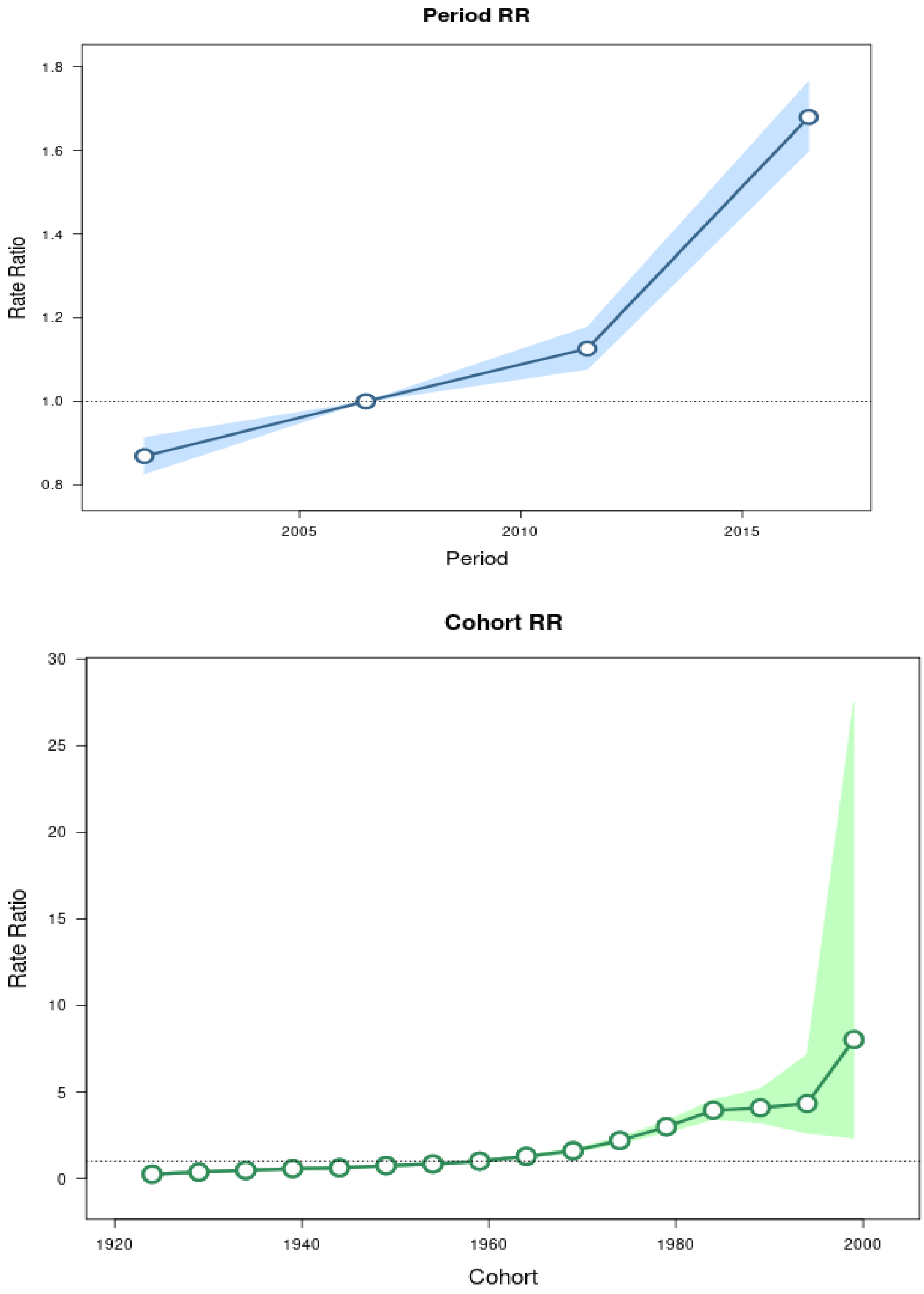
Period RR
Rate Ratio
1.8
1.6
1.4
1.2
1.0
0.8
2005
2010
2015
Period
Cohort RR
Rate Ratio
30
25
20
15
10
5
0
1920
1940
1960
1980
2000
Cohort

(**B**)

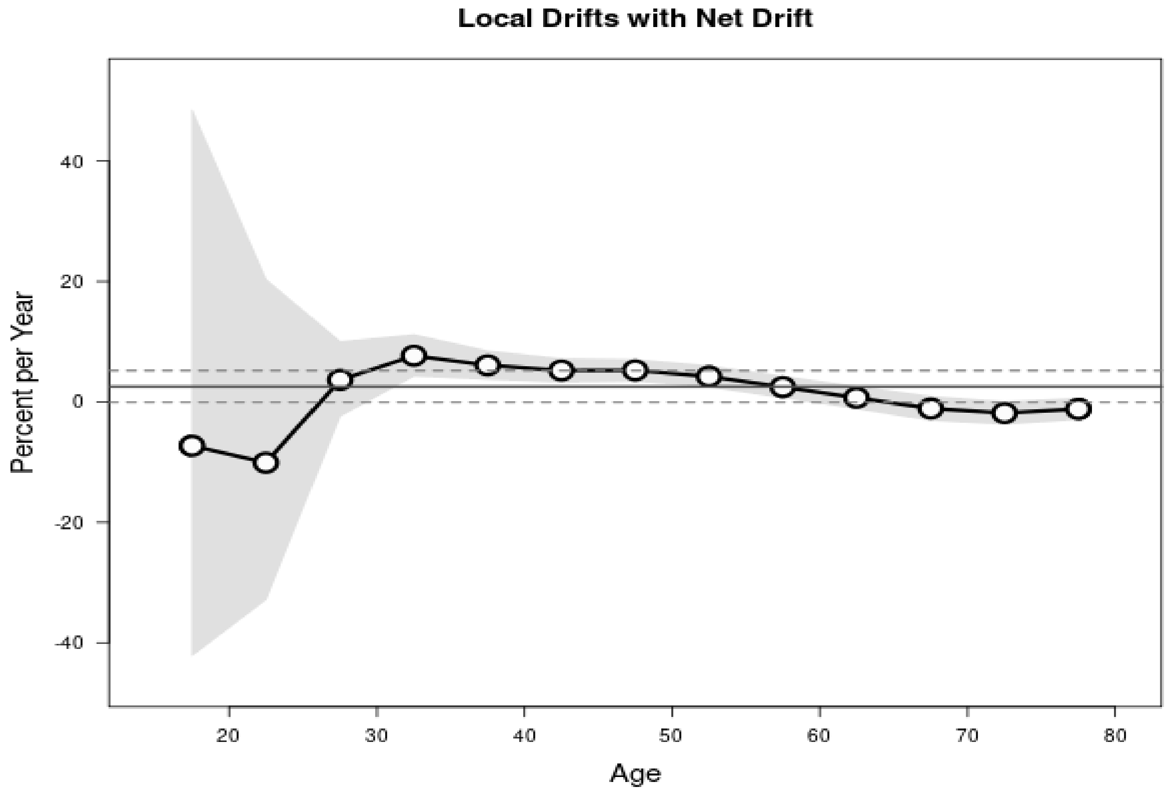
Local Drifts with Net Drift
Percent per Year
40
20
0
-20
-40
20
30
40
50
60
70
80
Age

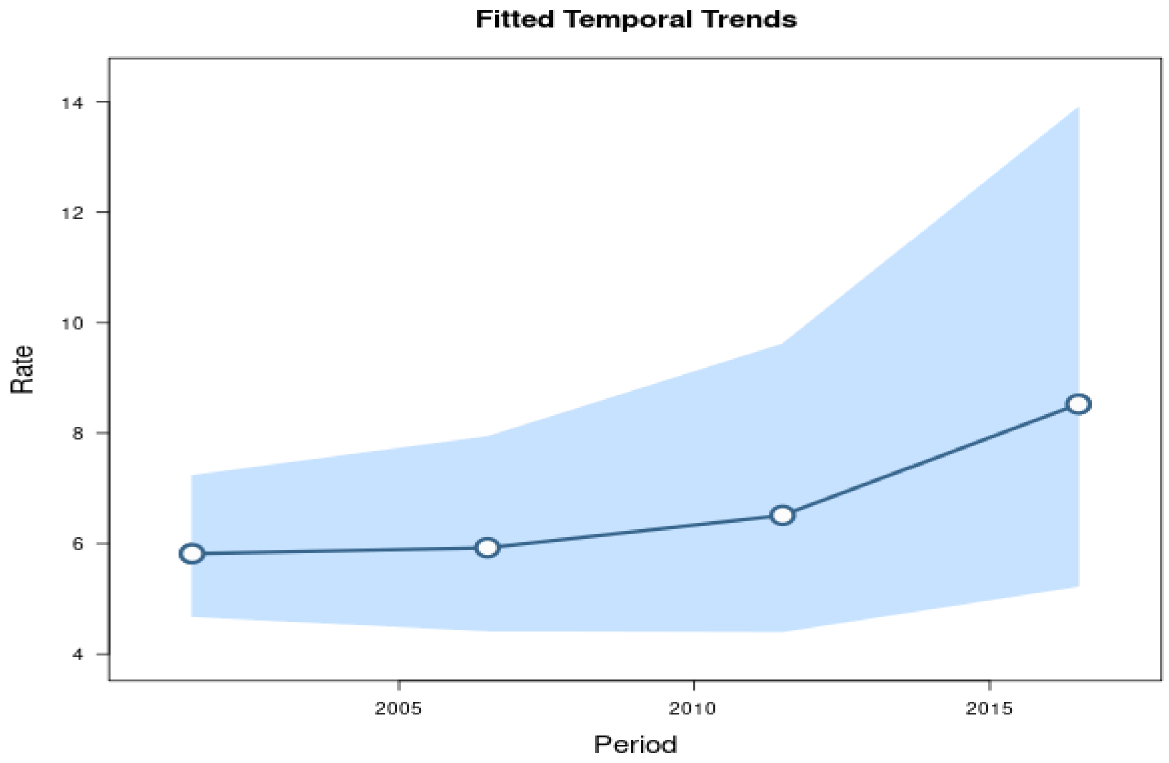
Fitted Temporal Trends
Rate
14
12
10
8
6
4
2005
2010
2015
Period

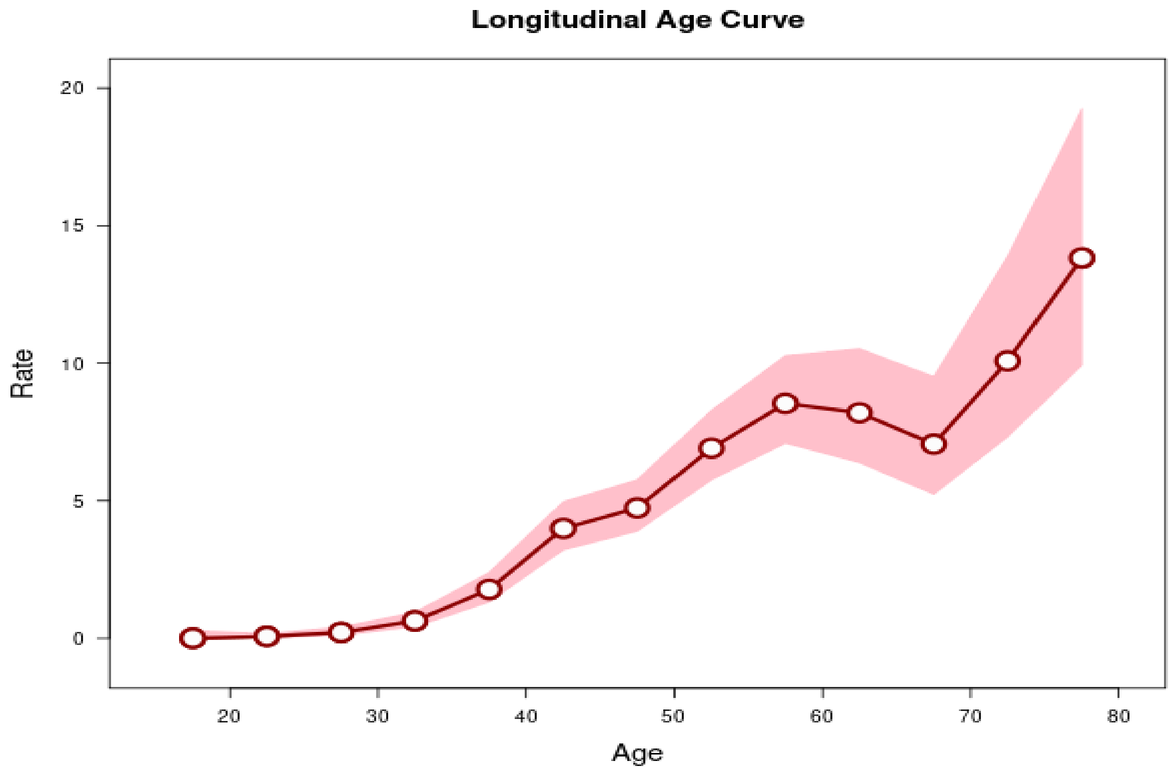
Longitudinal Age Curve
Rate
Age
0
5
10
15
20
20
30
40
50
60
70
80

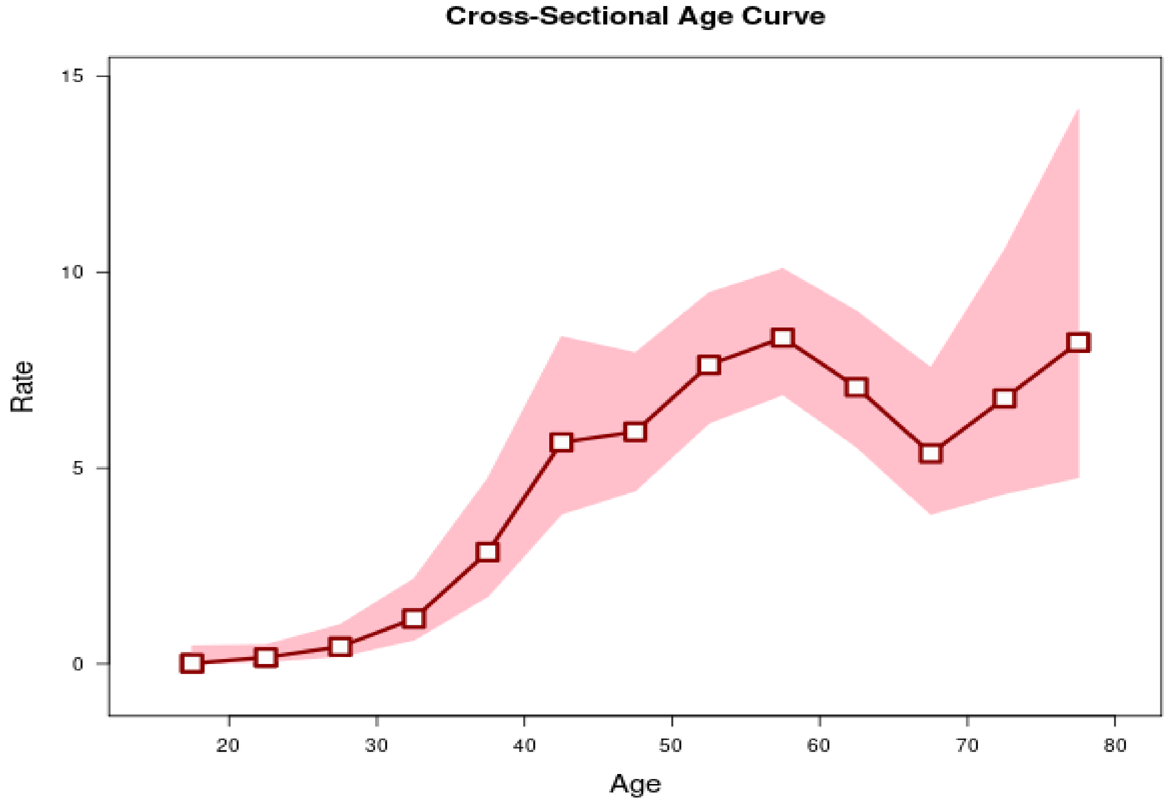
Cross-Sectional Age Curve
Rate
Age
0
5
10
15
20
30
40
50
60
70
80

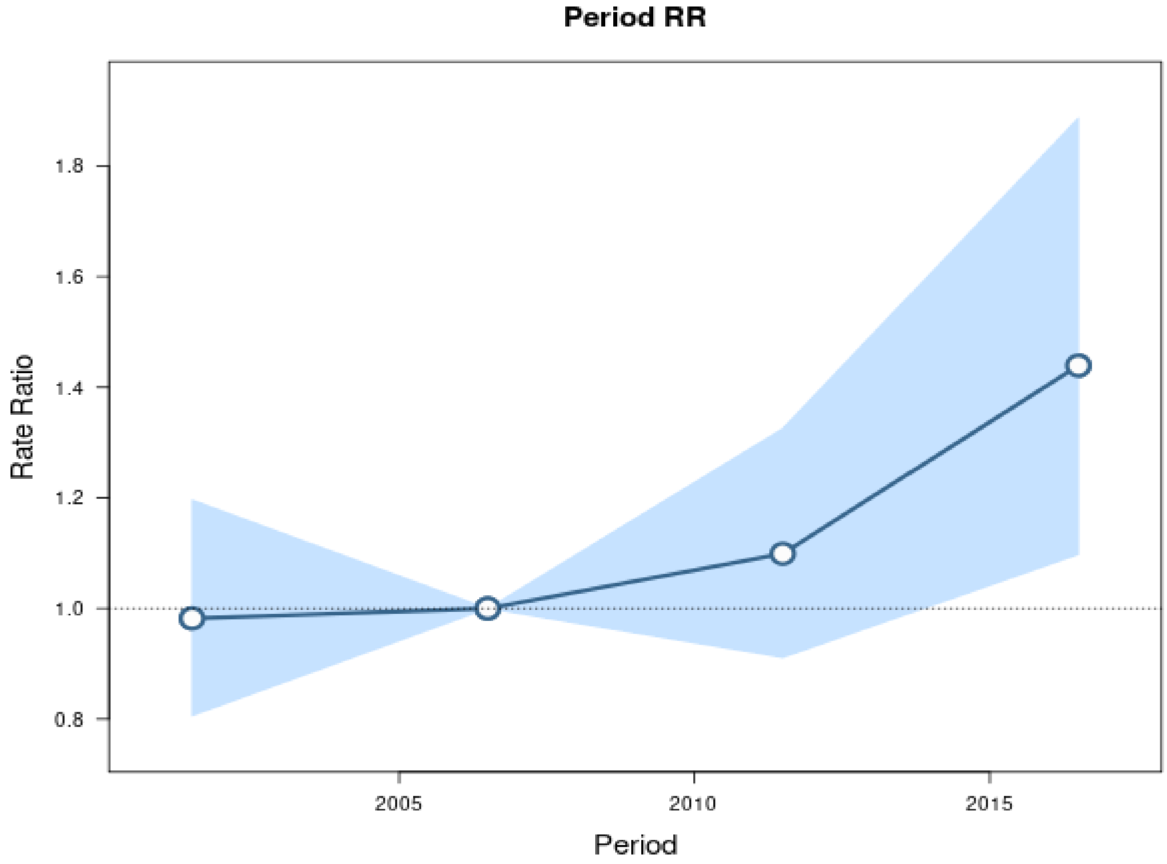
Period RR
Rate Ratio
1.8
1.6
1.4
1.2
1.0
0.8
2005
2010
2015
Period

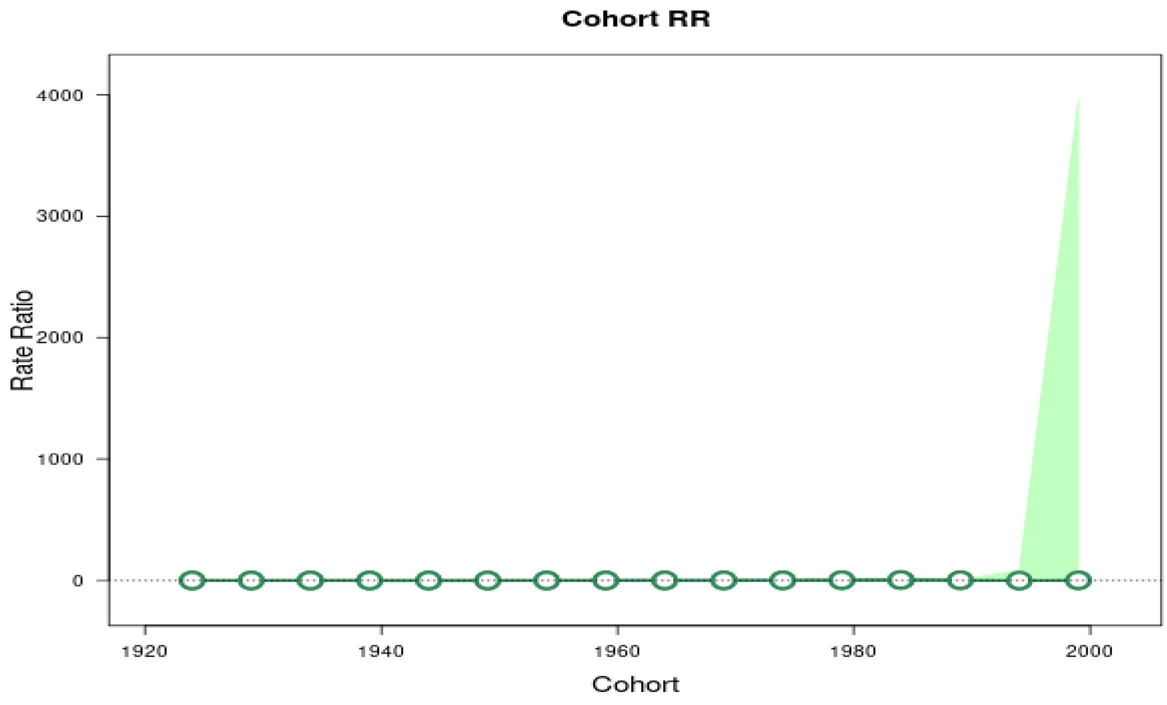
Cohort RR
Rate Ratio
4000
3000
2000
1000
0
1920
1940
1960
1980
2000
Cohort

(C)

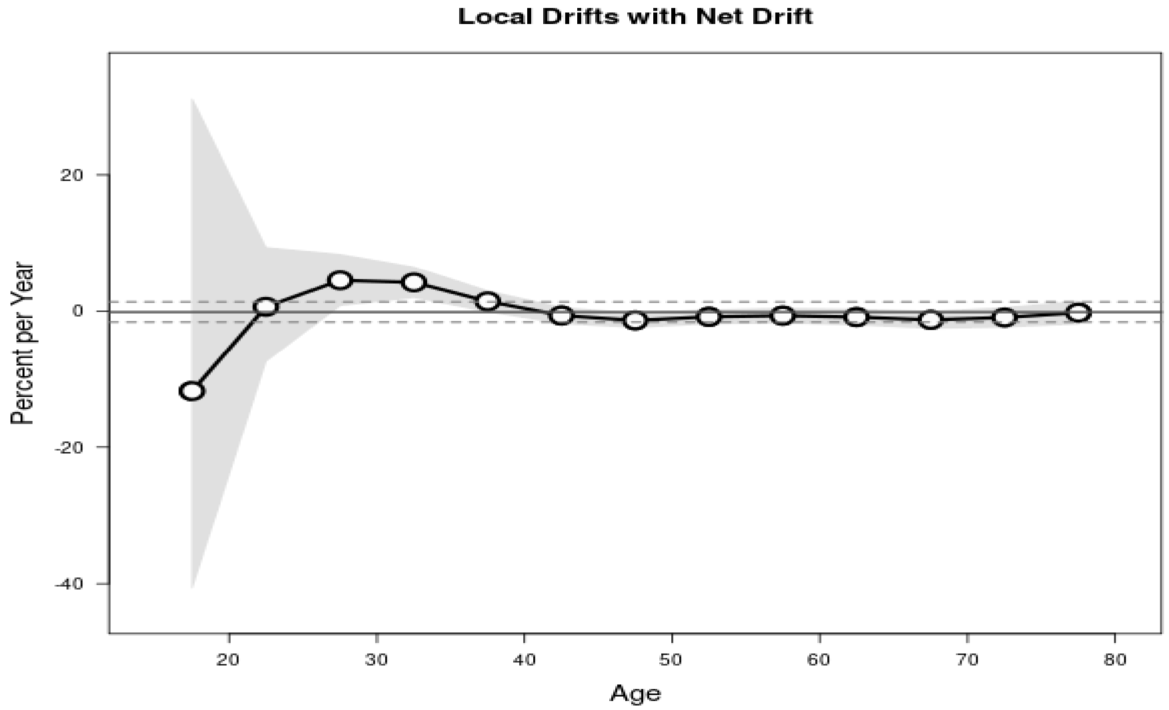
Local Drifts with Net Drift
Percent per Year
20
0
-20
-40
20
30
40
50
60
70
80
Age

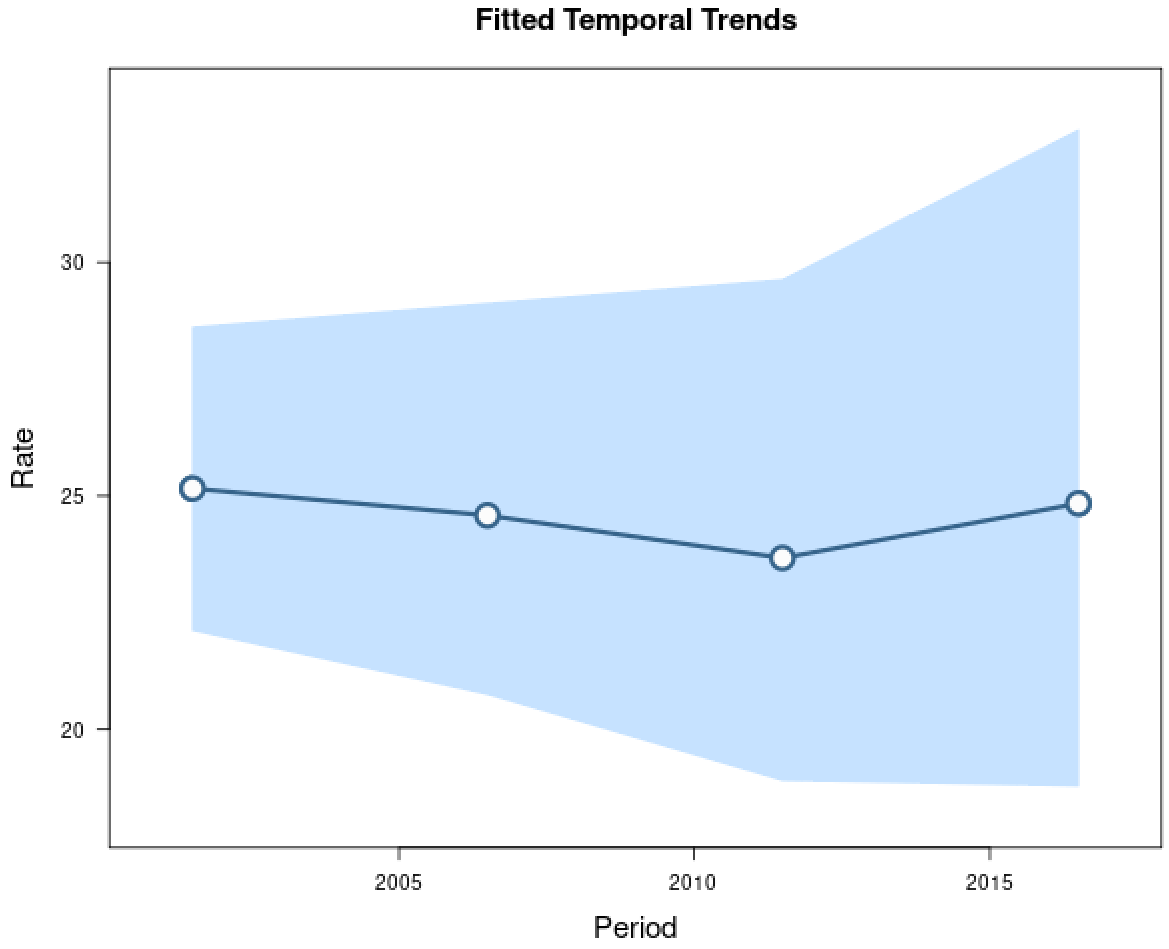
Fitted Temporal Trends
Rate
30
25
20
2005
2010
2015
Period

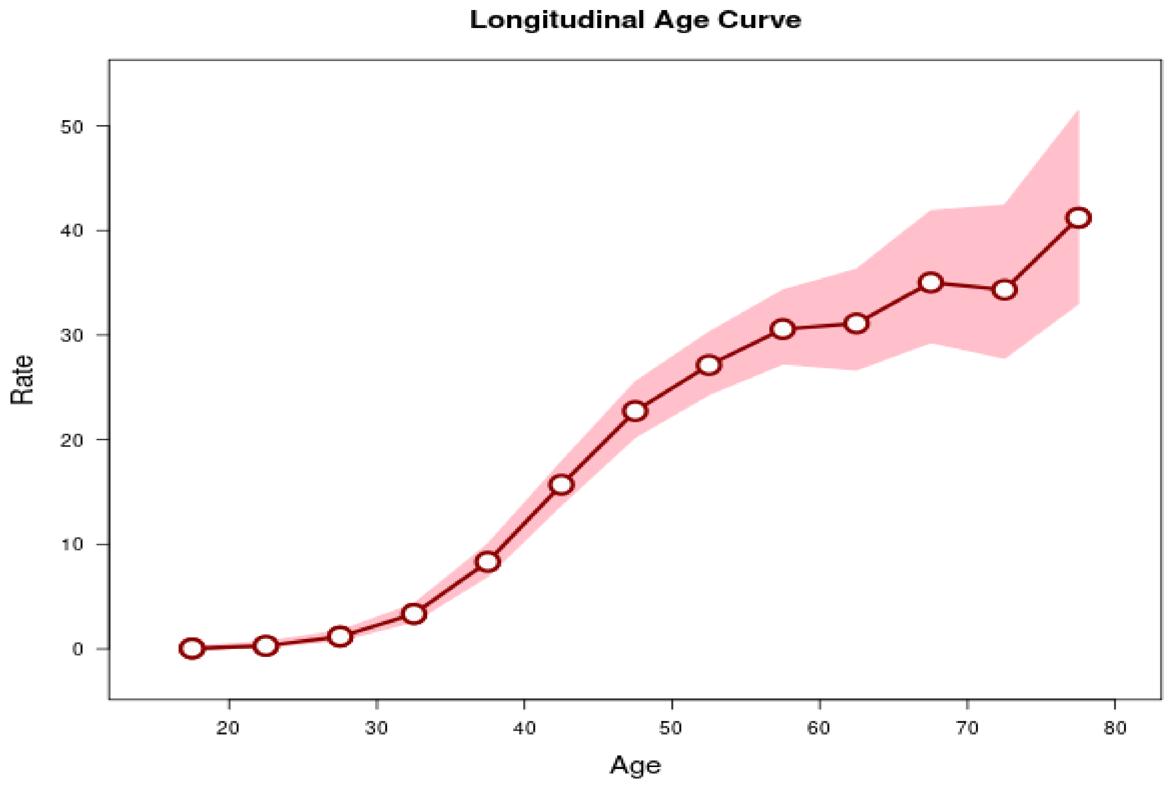
Longitudinal Age Curve
Rate
Age
0
10
20
30
40
50
20
30
40
50
60
70
80

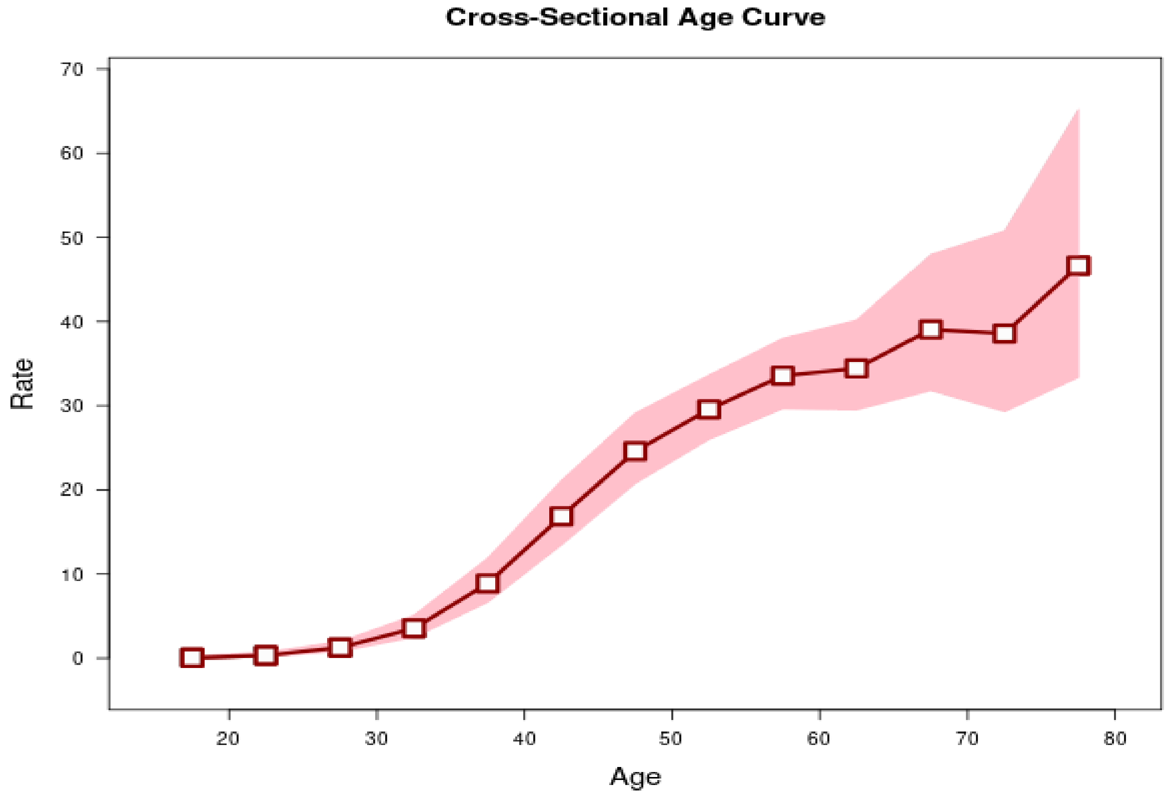
Cross-Sectional Age Curve
Rate
Age
0
10
20
30
40
50
60
70
20
30
40
50
60
70
80

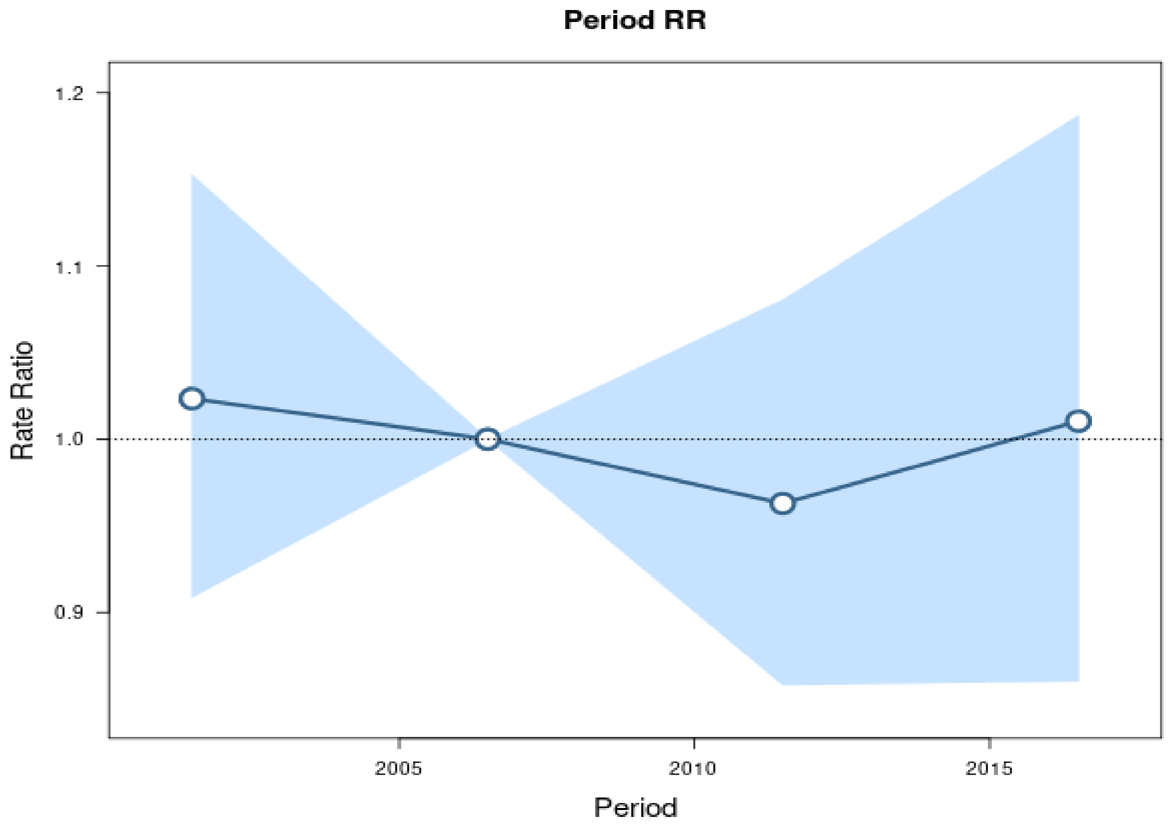

Period RR
1.2
1.1
1.0
0.9
Rate Ratio
2005
2010
2015
Period

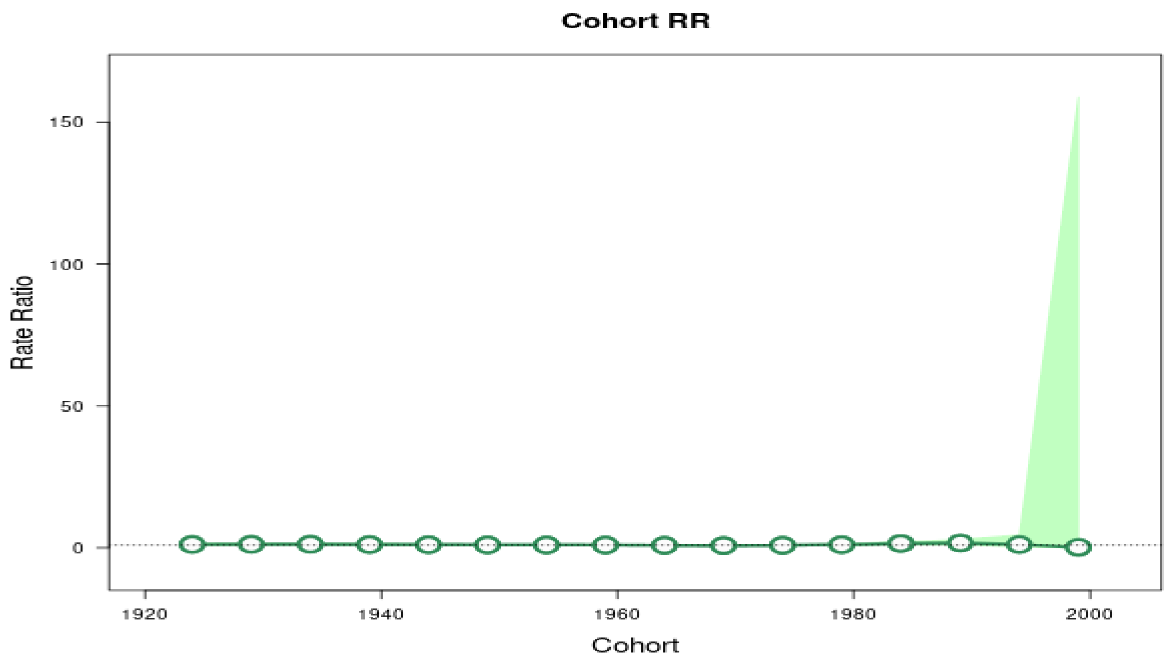

Cohort RR
150
100
50
0
Rate Ratio
1920
1940
1960
1980
2000
Cohort

(D)

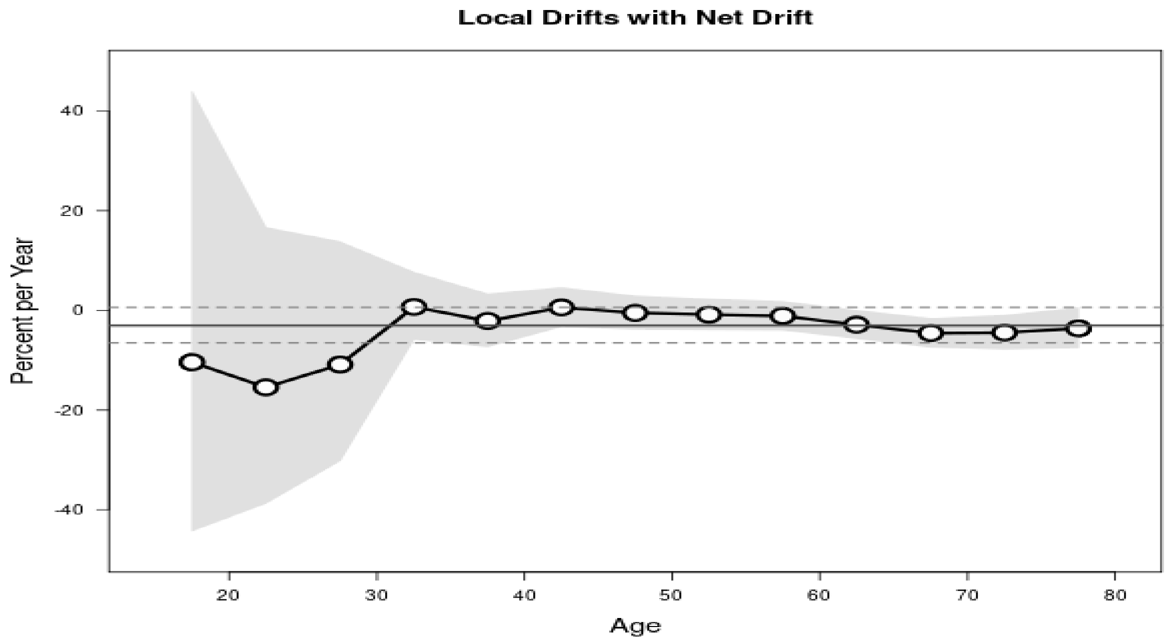
Local Drifts with Net Drift
Percent per Year
40
20
0
-20
-40
20
30
40
50
60
70
80
Age

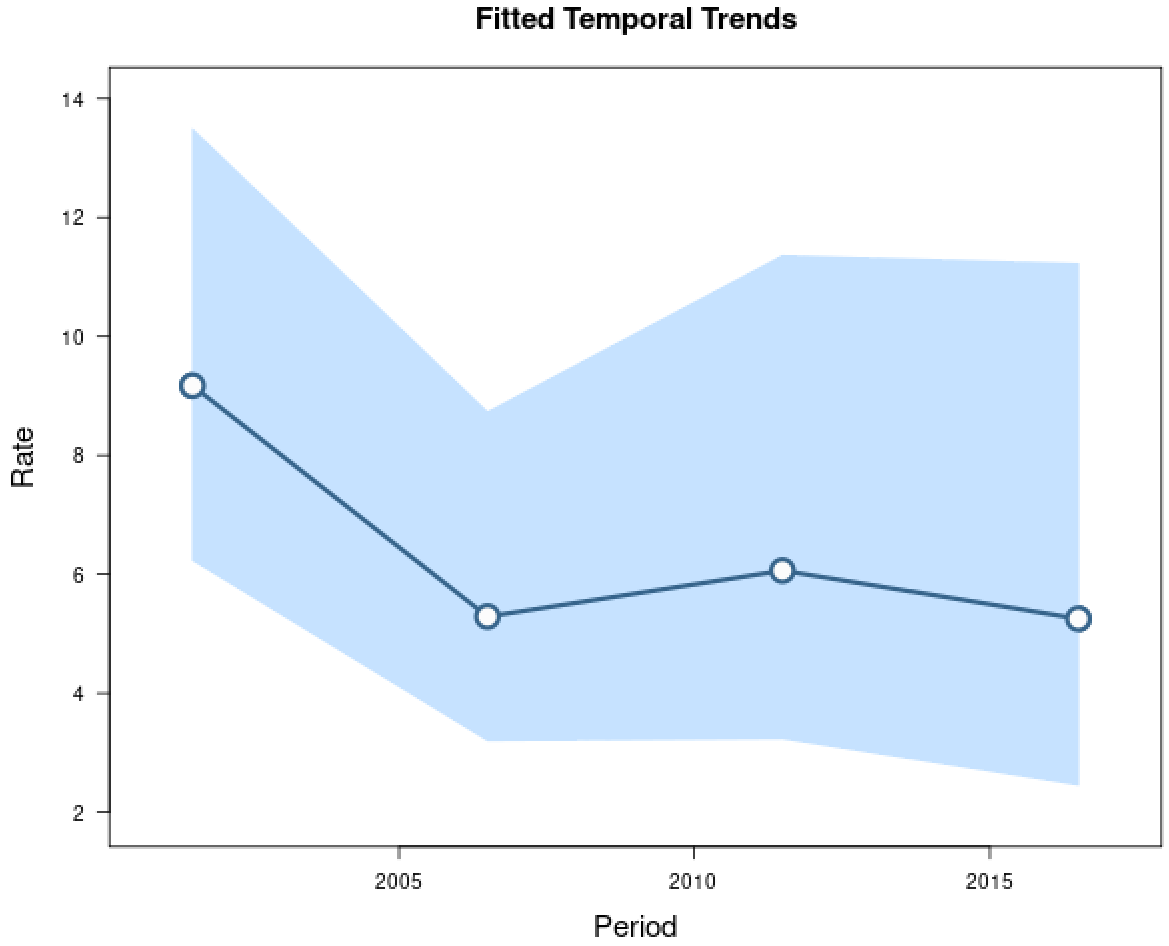
Fitted Temporal Trends
Rate
14
12
10
8
6
4
2
2005
2010
2015
Period

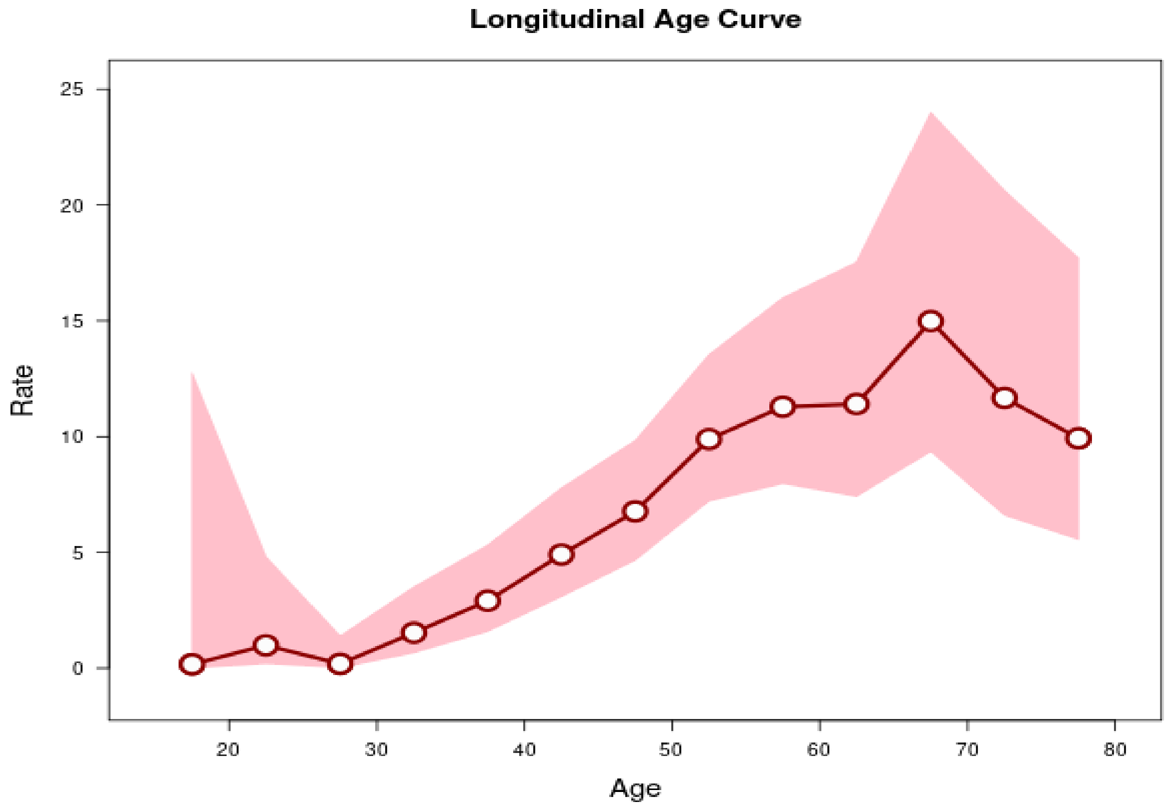
Longitudinal Age Curve
Rate
Age
25
20
15
10
5
0
20
30
40
50
60
70
80

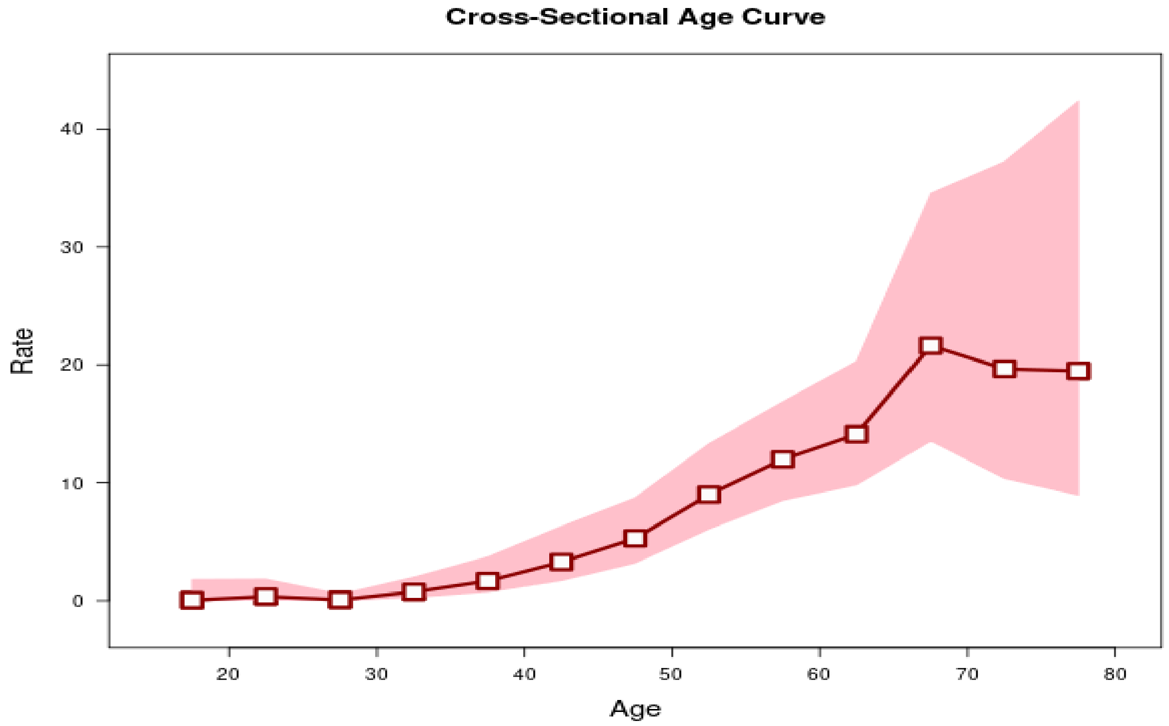
Cross-Sectional Age Curve
Rate
Age
40
30
20
10
0
20
30
40
50
60
70
80

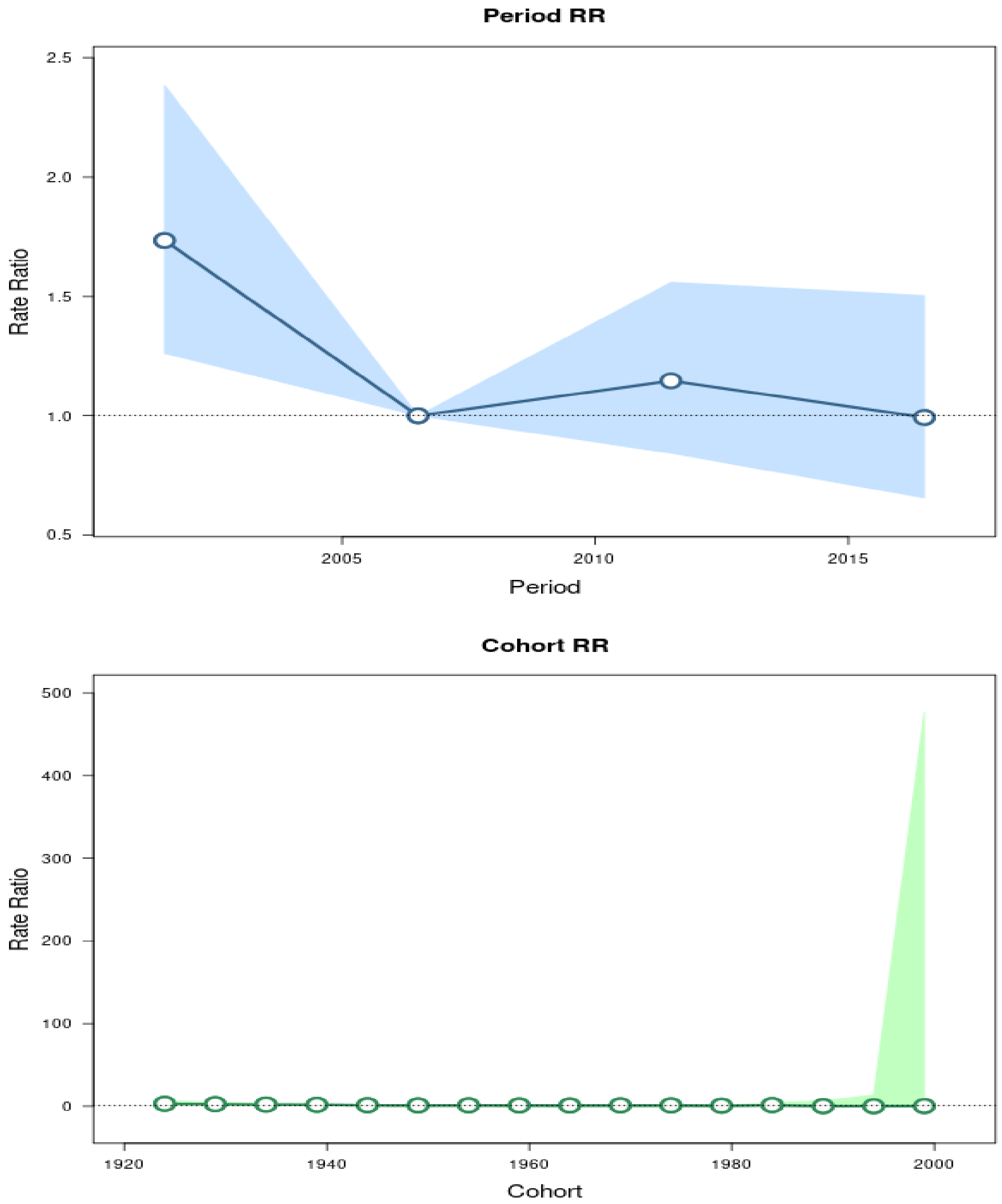

(**E**)

Figure S5. Age, period, and cohort effects of national (**A**); and ethnic trends of cervical cancer mortality in South Africa (1999–2018); Blacks (**B**), Whites (**C**), Coloureds (**D**), and Indians/Asians (**E**) (Local drift, fitted temporal trends, longitudinal age curve, cross sectional age curve, period effect, and cohort effect were depicted for each ethnic groups

Table S1. Trends in the mortality rate and mean age at death of Cervical cancer among ethnic groups in South Africa, 1999 - 2018.

Year	Black (n= 43,501)				White (n=1,836)				Coloured (n= 4,222)				Indian/Asian (n= 534)			
	Mortality (% of gynae & breast) ≥ 15 years	Age mean ± SD	CMR	ASMR	Mortality (% of gynae & breast) ≥ 15 years	Age mean ± SD	CMR	ASMR	Mortality (% of gynae & breast) ≥ 15 years	Age mean ± SD	CMR	ASMR	Mortality (% of gynae & breast) ≥ 15 years	Age mean ± SD	CMR	ASMR
1999	1,336	55.86±13.92	11.65	11.41	68	62.06±16.46	3.75	2.06	170	55.96±14.21	12.30	10.93	29	56.59±16.30	6.88	5.67
2000	1,486	55.55±14.13	12.52	12.22	81	63.43±15.59	4.47	2.37	183	54.39±13.76	12.88	11.00	24	62.75±11.09	5.57	4.93
2001	1,480	55.99±13.79	11.86	10.87	75	58.96±16.35	4.14	2.31	174	55.18±13.25	11.91	10.24	26	56.96±13.25	5.90	4.56
2002	1,557	56.13±14.10	12.31	11.13	72	59.72±17.24	3.92	2.13	174	53.92±13.23	11.75	9.92	21	57.57±11.40	4.70	3.88
2003	1,671	56.67±14.50	12.66	11.41	75	55.71±16.41	4.14	2.39	198	55.20±14.19	12.90	10.78	23	57.57±13.45	5.02	3.94
2004	1,686	55.97±14.31	13.73	13.56	76	60.68±15.58	4.16	2.20	194	56.38±13.08	13.30	12.35	19	62.32±14.15	4.39	3.46
2005	1,814	56.58±14.34	14.55	13.41	75	58.75±14.32	4.10	2.14	199	55.41±12.58	13.17	11.13	21	55.43±13.03	4.71	3.33
2006	1,793	55.38±14.50	14.17	12.89	82	57.94±15.04	4.47	2.33	194	55.73±13.18	12.62	10.32	23	57.43±13.80	5.08	3.56
2007	1,733	55.79±14.65	13.50	12.16	77	59.21±16.65	4.18	2.17	178	54.53±13.06	11.39	9.05	21	57.95±11.43	4.57	3.23
2008	1,781	56.83±14.87	13.23	12.26	82	58.09±15.82	4.32	2.32	171	55.04±14.12	10.38	8.40	21	58.76±11.65	4.32	3.04
2009	1,943	56.19±15.03	14.18	13.05	78	59.35±15.34	4.27	2.30	204	55.03±13.11	12.21	9.66	29	58.31±10.36	5.80	3.95
2010	1,982	56.47±15.01	14.33	13.46	75	59.55±16.81	3.87	2.06	188	55.97±12.87	11.13	8.63	34	57.76±11.70	6.67	4.58
2011	2,287	56.13±14.92	16.26	14.41	87	58.75±17.12	4.50	2.41	234	54.79±13.62	13.54	10.25	28	59.46±13.95	5.53	3.76
2012	2,484	55.80±14.91	17.45	15.39	98	56.87±15.56	5.01	2.73	224	53.77±13.86	12.80	9.75	25	58.32±12.21	4.87	3.27
2013	2,656	55.46±14.93	17.50	14.73	105	55.51±15.85	5.36	3.01	226	57.60±14.43	12.46	9.49	31	60.97±12.70	5.99	4.06
2014	2,918	55.25±15.05	19.25	15.78	107	57.17±15.28	5.51	3.00	281	55.04±13.79	15.44	11.60	29	59.72±13.14	5.56	3.74
2015	3,059	55.24±15.08	19.74	16.71	112	58.37±14.08	5.76	3.05	259	55.75±13.96	13.94	10.05	30	57.67±12.25	5.65	3.65
2016	3,197	55.10±14.90	20.12	17.06	131	54.51±15.03	6.76	3.91	281	55.30±13.75	15.21	10.96	28	62.25±13.23	5.18	3.16
2017	3,291	55.21±14.76	20.33	17.09	143	56.70±15.24	7.36	3.90	238	56.38±13.61	12.69	8.75	31	55.58±15.68	5.59	3.33
2018	3,347	55.19±14.91	20.20	16.74	137	55.02±14.41	6.96	3.86	252	54.79±14.48	13.04	8.54	41	56.90±13.23	7.05	4.16

Table S2. Join point estimates of the national and ethnic trends in age specific mortality rates for cervical cancers South Africa, 1999 – 2018

Cervix	Trend segment	Lower Endpoint	Upper Endpoint	AAPC	Lower CI	Upper CI	Test Statistic~	P-Value~	Comment
**Overall**									
15-19	Full Range	1999	2018	-1.1	-5.1	3.2	-0.5	0.6	Non-Statistically significant decrease
20-24	1	1999	2006	8.6	-5.0	24.2	1.3	0.2	Non-Statistically significant increase
	2	2006	2018	-3.1	-8.3	2.4	-1.2	0.2	Non-Statistically significant decrease
	Full Range	1999	2018	0.2	-2.6	3.1	0.2	0.9	Stable
25-29	1	1999	2014	3.9*	2.3	5.7	5.0	0.0	Statistically significant increase
	2	2014	2018	-9.3	-19.4	2.0	-1.8	0.1	Non-Statistically significant decrease
	Full Range	1999	2018	2.2*	0.7	3.6	3.2	< 0.001	Statistically significant increase
30-34	1	1999	2015	4.1*	2.7	5.6	6.4	< 0.001	Statistically significant increase
	2	2015	2018	-2.5	-16.6	13.9	-0.3	0.7	Non-Statistically significant decrease
	Full Range	1999	2018	3.5*	2.6	4.5	7.7	< 0.001	Statistically significant increase
35-39	1	1999	2006	2.6	-1.5	7.0	1.4	0.2	Non-Statistically significant increase
	2	2006	2009	-3.3	-29.8	33.2	-0.2	0.8	Non-Statistically significant decrease
	3	2009	2018	5.2*	2.5	7.9	4.3	< 0.001	Statistically significant increase
	Full Range	1999	2018	2.6*	1.6	3.5	5.8	<0.001	Non-Statistically significant increase
40-44	1	1999	2007	3.5*	1.4	5.7	3.6	< 0.001	Statistically significant increase
	2	2007	2013	-0.3	-4.3	3.8	-0.2	0.9	Stable

	3	2013	2018	5.0*	1.1	9.1	2.8	< 0.001	Statistically significant increase
	Full Range	1999	2018	2.1*	1.5	2.8	7.0	< 0.001	Statistically significant increase
45-49	1	1999	2001	-4.6	-24.7	20.8	-0.4	0.7	Non-Statistically significant decrease
	2	2001	2018	1.4*	0.6	2.2	3.8	< 0.001	Statistically significant increase
	Full Range	1999	2018	1.2*	0.5	1.8	4.0	< 0.001	Statistically significant increase
50-54	1	1999	2013	-0.1	-1.0	0.9	-0.2	0.9	Stable
	2	2013	2018	3.2	-1.2	7.7	1.5	0.1	Non-Statistically significant increase
	Full Range	1999	2018	0.5	-0.1	1.2	1.9	0.1	Stable
55-59	1	1999	2003	3.4	-1.8	8.9	1.4	0.2	Non-Statistically significant increase
	2	2003	2012	-1.6	-3.3	0.2	-1.9	0.1	Non-Statistically significant decrease
	3	2012	2018	3.1*	0.3	5.9	2.4	< 0.001	Statistically significant increase
	Full Range	1999	2018	0.2	-0.4	0.8	0.7	0.5	Stable
60-64	1	1999	2004	3.2	-0.0	6.5	2.2	0.1	Non-Statistically significant increase
	2	2004	2007	-6.6	-19.1	7.8	-1.0	0.3	Non-Statistically significant decrease
	3	2007	2018	1.4*	0.5	2.4	3.2	< 0.001	Statistically significant increase
	Full Range	1999	2018	0.0	-0.6	0.6	0.1	0.9	Stable
65-69	1	1999	2013	-0.9	-2.0	0.2	-1.8	0.1	Non-Statistically significant decrease
	2	2013	2018	2.4	-3.1	8.1	0.9	0.4	Non-Statistically significant decrease
	Full Range	1999	2018	-0.3	-1.0	0.4	-0.9	0.4	Stable
70-74	1	1999	2004	5.2*	0.3	10.4	2.2	< 0.001	Statistically significant increase
	2	2004	2018	-1.0*	-1.9	-0.0	-2.2	< 0.001	Statistically significant increase

	Full Range	1999	2018	0.1	-0.9	1.0	0.2	0.9	Stable
75+	1	1999	2005	13.0*	7.9	18.3	5.6	< 0.001	Statistically significant increase
	2	2005	2018	-0.9	-2.1	0.4	-1.5	0.2	Non-Statistically significant decrease
	Full Range	1999	2018	1.9*	0.4	3.4	2.6	< 0.001	Statistically significant increase
**Blacks**									
15-19	Full Range	1999	2018	4.9*	0.3	9.7	2.2	< 0.001	Statistically significant increase
20-24	1	1999	2006	15.2*	0.2	32.5	2.3	< 0.001	Statistically significant increase
	2	2006	2011	-13.9	-34.5	13.2	-1.2	0.2	Non-Statistically significant decrease
	3	2011	2014	25.7	-53.3	238.7	0.5	0.6	Non-Statistically significant increase
	4	2014	2018	-10.8	-34.3	21.0	-0.8	0.4	Non-Statistically significant decrease
	Full Range	1999	2018	1.4	-1.8	4.7	0.9	0.4	Non-Statistically significant increase
25-29	1	1999	2014	4.7*	2.1	7.4	3.9	<0.001	Statistically significant increase
	2	2014	2018	-7.2	-22.0	10.4	-0.9	0.4	Non-Statistically significant decrease
	Full Range	1999	2018	3.0*	1.2	4.9	3.5	<0.001	Statistically significant increase
30-34	1	1999	2015	5.9*	4.3	7.5	8.0	<0.001	Statistically significant increase
	2	2015	2018	-3.6	-18.4	13.8	-0.5	0.6	Non-Statistically significant decrease
	Full Range	1999	2018	5.0*	3.8	6.2	8.9	<0.001	Statistically significant increase
35-39	1	1999	2006	3.9	-1.6	9.7	1.6	0.1	Non-Statistically significant increase
	2	2006	2009	-6.4	-38.6	42.7	-0.4	0.7	Non-Statistically significant decrease

	3	2009	2014	13.2*	0.4	27.7	2.3	<0.001	Statistically significant increase
	4	2014	2018	-0.1	-9.3	10.1	-0.0	1.0	Non-Statistically significant decrease
	Full Range	1999	2018	4.1*	3.0	5.3	7.5	<0.001	Statistically significant increase
40-44	1	1999	2006	4.8*	0.5	9.3	2.4	<0.001	Statistically significant increase
	2	2006	2018	3.5*	1.9	5.1	4.7	<0.001	Statistically significant increase
	Full Range	1999	2018	3.8*	3.1	4.6	10.5	<0.001	Statistically significant increase
45-49	1	1999	2008	1.1	-1.5	3.9	0.9	0.4	Non-Statistically significant increase
	2	2008	2018	4.0*	1.9	6.1	4.1	<0.001	Statistically significant increase
	Full Range	1999	2018	2.7*	2.0	3.5	7.4	<0.001	Statistically significant increase
50-54	1	1999	2008	0.5	-2.6	3.7	0.3	0.7	Stable
	2	2008	2018	4.0*	1.5	6.5	3.4	<0.001	Statistically significant increase
	Full Range	1999	2018	2.4*	1.5	3.4	5.7	<0.001	Statistically significant increase
55-59	1	1999	2003	3.8	-1.6	9.5	1.5	0.2	Non-Statistically significant increase
	2	2003	2008	-2.6	-7.6	2.7	-1.1	0.3	Non-Statistically significant decrease
	3	2008	2018	3.7*	2.4	4.9	6.5	<0.001	Statistically significant increase
	Full Range	1999	2018	1.6*	0.9	2.3	4.9	<0.001	Statistically significant increase
60-64	1	1999	2004	4.1	-1.7	10.2	1.5	0.2	Non-Statistically significant increase
	2	2004	2007	-5.2	-25.9	21.4	-0.5	0.6	Non-Statistically significant decrease
	3	2007	2018	3.1*	1.5	4.8	4.2	<0.001	Statistically significant increase

	Full Range	1999	2018	1.6*	0.8	2.3	4.3	<0.001	Statistically significant increase
65-69	Full Range	1999	2018	1.4*	0.5	2.3	3.3	<0.001	Statistically significant increase
70-74	1	1999	2004	7.4	-1.5	17.1	1.8	0.1	Non-Statistically significant increase
	2	2004	2018	1.6*	0.1	3.1	2.2	< 0.001	Statistically significant increase
	Full Range	1999	2018	2.5*	1.3	3.7	4.4	<0.001	Statistically significant increase
75+	1	1999	2001	-18.4	-43.4	17.6	-1.2	0.3	Non-Statistically significant decrease
	2	2001	2018	2.9*	1.5	4.3	4.4	<0.001	Statistically significant increase
	Full Range	1999	2018	1.8*	0.2	3.3	2.4	<0.001	Statistically significant increase
**Indian/Asian**									
15-19	0								
20-24	Full Range	1999	2018	-5.6	-16.0	6.0	-1.0	0.3	Non-Statistically significant decrease
25-29	1	1999	2013	116.4*	116.4	116.4	8.3×10^{14}	<0.001	Statistically significant increase
	2	2013	2018	-86.9*	-86.9	-86.9	-4.6×10^{14}	<0.001	Statistically significant decrease
	Full Range	1999	2018	86.8*	27.5	173.8	3.4	<0.001	Statistically significant increase
30-34	1	1999	2005	-23.8*	-34.7	-11.1	-3.8	<0.001	Statistically significant decrease
	2	2005	2018	8.6*	1.8	15.7	2.7	<0.001	Statistically significant increase
	Full Range	1999	2018	-2.8	-6.4	0.9	-1.6	0.1	Non-Statistically significant decrease
35-39	1	1999	2005	14.5	-46.4	144.4	0.4	0.7	Non-Statistically significant increase
	2	2005	2018	-9.1*	-15.5	-2.3	-2.8	<0.001	Statistically significant decrease

	Full Range	1999	2018	-4.6	-9.2	0.2	-2.0	0.1	Non-Statistically significant decrease
40-44	1	1999	2002	71.2	-74.1	1033.1	0.6	0.6	Non-Statistically significant increase
	2	2002	2018	0.2	-4.3	5.0	0.1	0.9	Stable
	Full Range	1999	2018	1.5	-2.4	5.4	0.8	0.4	Non-Statistically significant increase
45-49	1	1999	2015	0.4	-4.9	6.0	0.1	0.9	Stable
	2	2015	2018	-26.5	-67.3	65.3	-0.8	0.4	Non-Statistically significant decrease
	Full Range	1999	2018	-1.4	-5.1	2.5	-0.8	0.5	Non-Statistically significant decrease
50-54	Full Range	1999	2018	2.6	-1.2	6.6	1.4	0.2	Non-Statistically significant increase
55-59	1	1999	2001	4.1	-55.4	143.0	0.1	0.9	Non-Statistically significant increase
	2	2001	2004	-35.2	-79.6	106.5	-0.8	0.4	Non-Statistically significant decrease
	3	2004	2009	32.0	-7.7	88.8	1.8	0.1	Non-Statistically significant increase
	4	2009	2018	-10.4*	-19.5	-0.3	-2.3	<0.001	Statistically significant decrease
	Full Range	1999	2018	-1.9	-5.5	1.9	-1.0	0.3	Non-Statistically significant decrease
	Full Range	1999	2018	-0.6	-3.2	2.1	-0.5	0.7	Non-Statistically significant decrease
65-69	1	1999	2006	-14.8*	-26.3	-1.6	-2.4	<0.001	Statistically significant decrease
	2	2006	2018	3.4	-3.8	11.2	1.0	0.3	Non-Statistically significant increase
	Full Range	1999	2018	-3.4*	-6.5	-0.3	-2.3	<0.001	Statistically significant decrease
70-74	Full Range	1999	2018	-6.4*	-10.1	-2.5	-3.4	<0.001	Statistically significant decrease
75+	1	1999	2008	-8.6	-18.4	2.4	-1.7	0.1	Non-Statistically significant decrease

	2	2008	2018	4.3	-6.4	16.2	0.8	0.4	Non-Statistically significant increase
	Full Range	1999	2018	-2.3	-5.6	1.2	-1.4	0.2	Non-Statistically significant decrease
**Coloured**									
15-19	1	1999	2003	-87.2	-98.5	7.0	-2.1	0.1	Non-Statistically significant decrease
	2	2003	2018	0.0	-26.7	36.4	0.0	2.0×10^{30}	Stable
	Full Range	1999	2018	-38.9*	-47.7	-28.7	-6.7	< 0.001	Statistically significant decrease
20-24	1	1999	2012	6.9	-1.4	15.8	1.8	0.1	Non-Statistically significant increase
	2	2012	2018	-17.8*	-31.1	-1.9	-2.4	<0.001	Statistically significant decrease
	Full Range	1999	2018	0.0	-5.5	5.9	0.0	1.0	Stable
25-29	1	1999	2012	9.8*	1.7	18.6	2.6	<0.001	Statistically significant increase
	2	2012	2018	-6.2	-22.9	14.1	-0.7	0.5	Non-Statistically significant decrease
	Full Range	1999	2018	5.2*	0.8	9.8	2.5	<0.001	Statistically significant increase
30-34	1	1999	2001	-16.0	-77.6	215.4	-0.3	0.8	Non-Statistically significant decrease
	2	2001	2018	4.5*	0.3	9.0	2.3	<0.001	Statistically significant increase
	Full Range	1999	2018	3.6*	0.5	6.8	2.4	<0.001	Statistically significant increase
35-39	1	1999	2002	10.3	-26.6	65.9	0.5	0.6	Non-Statistically significant increase
	2	2002	2008	-8.8	-24.4	9.9	-1.1	0.3	Non-Statistically significant decrease
	3	2008	2018	7.5*	1.2	14.1	2.6	<0.001	Statistically significant increase
	Full Range	1999	2018	1.3	-0.9	3.6	1.2	0.2	Non-Statistically significant increase
40-44	1	1999	2001	5.2	-42.1	91.4	0.2	0.9	Non-Statistically significant increase

	2	2001	2007	-4.6	-17.5	10.2	-0.7	0.5	Non-Statistically significant decrease
	3	2007	2010	-9.1	-54.0	79.5	-0.3	0.8	Non-Statistically significant decrease
	4	2010	2018	7.4*	0.2	15.1	2.3	<0.001	Statistically significant increase
	Full Range	1999	2018	-0.7	-2.7	1.3	-0.7	0.5	Non-Statistically significant decrease
45-49	1	1999	2016	0.4	-1.9	2.7	0.4	0.7	Non-Statistically significant increase
	2	2016	2018	-22.3	-64.0	67.6	-0.7	0.5	Non-Statistically significant decrease
	Full Range	1999	2018	-0.4	-2.2	1.5	-0.4	0.7	Stable
50-54	1	1999	2005	0.2	-5.6	6.4	0.1	0.9	Stable
	2	2005	2018	-2.4*	-4.3	-0.5	-2.6	<0.001	Statistically significant increase
	Full Range	1999	2018	-1.7*	-2.7	-0.7	-3.6	<0.001	Statistically significant increase
55-59	1	1999	2001	10.0	-28.1	68.2	0.5	0.6	Non-Statistically significant increase
	2	2001	2018	-1.0	-2.5	0.4	-1.5	0.1	Non-Statistically significant decrease
	Full Range	1999	2018	-0.6	-1.7	0.5	-1.2	0.3	Non-Statistically significant decrease
60-64	Full Range	1999	2018	-0.7	-2.7	1.3	-0.8	0.4	Non-Statistically significant decrease
65-69	Full Range	1999	2018	-1.5	-3.2	0.2	-1.9	0.1	Non-Statistically significant decrease
70-74	Full Range	1999	2018	-2.1*	-4.1	-0.1	-2.2	<0.001	Statistically significant decrease
75+	1	1999	2004	7.7	-2.9	19.5	1.6	0.1	Non-Statistically significant increase
	2	2004	2009	-9.9	-23.4	6.0	-1.5	0.2	Non-Statistically significant decrease
	3	2009	2014	14.7	-0.8	32.5	2.1	0.1	Non-Statistically significant increase

	4	2014	2018	-16.4*	-29.0	-1.5	-2.5	<0.001	Statistically significant decrease
	Full Range	1999	2018	0.2	-1.7	2.3	0.3	0.8	Stable
**White**									
15-19	-	-	-						
20-24	Full Range	1999	2018	-1.4	-6.0	3.3	-0.6	0.5	Non-Statistically significant decrease
25-29	Full Range	1999	2018	0.2	-4.3	4.9	0.1	0.9	Stable
30-34	Full Range	1999	2018	7.2*	2.5	12.1	3.3	<0.001	Statistically significant increase
35-39	Full Range	1999	2018	6.1*	2.7	9.5	3.8	<0.001	Statistically significant increase
40-44	Full Range	1999	2018	6.6*	3.8	9.5	5.0	<0.001	Statistically significant increase
45-49	Full Range	1999	2018	5.2*	2.9	7.6	4.7	<0.001	Statistically significant increase
50-54	1	1999	2010	0.9	-3.5	5.5	0.4	0.7	Non-Statistically significant decrease
	2	2010	2018	10.9*	5.1	17.1	4.1	<0.001	Statistically significant increase
	Full Range	1999	2018	5.3*	3.2	7.3	5.5	<0.001	Statistically significant increase
55-59	1	1999	2001	62.7	-42.7	361.5	1.0	0.3	Non-Statistically significant increase
	2	2001	2014	-3.5	-7.9	1.1	-1.6	0.1	Non-Statistically significant decrease
	3	2014	2018	27.0*	1.7	58.5	2.3	<0.001	Statistically significant increase
	Full Range	1999	2018	2.4	-0.3	5.2	1.9	0.1	Non-Statistically significant increase
60-64	Full Range	1999	2018	1.1	-1.5	3.7	0.9	0.4	Non-Statistically significant increase
65-69	1	1999	2002	-18.0	-35.8	4.9	-1.8	0.1	Non-Statistically significant decrease
	2	2002	2005	11.3	-33.7	86.8	0.5	0.7	Non-Statistically significant increase

	3	2005	2009	-16.6	-38.6	13.3	-1.3	0.2	Non-Statistically significant decrease
	4	2009	2018	7.7*	1.6	14.1	2.9	<0.001	Statistically significant increase
	Full Range	1999	2018	-1.9	-4.2	0.6	-1.6	0.1	Non-Statistically significant decrease
70-74	Full Range	1999	2018	-0.5	-2.9	2.0	-0.4	0.7	Stable
75+	Full Range	1999	2018	-2.1*	-3.8	-0.3	-2.5	<0.001	Non-Statistically significant decrease

Table S3 The overall and ethnic age specific mortality rates of cervical cancers and in South Africa, 2018

**Age (Year)**	**15-19**	**20-24**	**25-29**	**30-34**	**35-39**	**40-44**	**45-49**	**50-54**	**55-59**	**60-64**	**65-69**	**70-74**	**≥75**
**Cervical cancer**													
Overall	0.17	0.44	1.76	7.36	17.08	25.27	33.08	37.45	40.96	44.22	45.53	53.94	59.76
Black	0.20	0.42	1.76	7.43	17.91	26.48	39.24	40.74	47.09	52.98	58.94	75.70	87.90
White	0.00	0.00	0.75	4.70	6.07	11.86	8.08	14.76	13.10	6.64	5.61	4.94	8.24
Indians/Asians	0.00	0.00	0.00	3.23	3.42	6.44	4.14	19.87	7.37	17.07	27.10	0.00	17.22
Coloured	0.00	0.47	2.32	4.91	11.82	19.80	13.54	26.59	27.48	23.27	20.88	28.07	38.12

Table S4. Age Period Cohort effect estimates of trends in Cervical cancer mortality in South Africa, 1999-2018.

**Factor**	**Overall**					
	**Longitudinal RR(adjusted for period effect)**	**95%CI**	**Cross sectional RR (Adjusted**	**95%CI**	**Local drift(%)**	**95%CI**

			for cohort effect)			
**Age**						
15-19	0.03	0.01-0.05	0.05	0.03-0.09	2.05	-4.68 to 9.26
20-24	0.22	0.17-0.28	0.39	0.31-0.48	-0.14	-2.78 to 2.57
25-29	1.07	0.93-1.22	1.79	1.58-2.03	2.63	1.37 - 3.89
30-34	3.64	3.31-4.00	5.65	5.15-6.21	4.33	3.57 - 5.10
35-39	9.67	9.01-10.39	13.94	12.90-15.07	3.74	3.18 - 4.31
40-44	18.50	17.52-19.54	24.72	23.16-26.39	2.00	1.54 - 2.47
45-49	27.09	25.85-28.39	33.57	31.75-35.49	1.34	0.91 - 1.77
50-54	33.92	32.44-35.48	38.98	37.11-40.94	0.75	0.34 - 1.17
55-59	40.32	38.48-42.26	42.96	40.99-45.03	0.31	-0.12 to 0.74
60-64	43.38	40.81-46.12	42.86	40.80-45.02	-0.06	-0.50 to 0.38
65-69	48.53	45.25-52.04	44.45	41.99-47.06	-0.48	-0.95 to -0.01
70-74	53.41	49.31-57.85	45.37	42.26-48.71	-0.19	-0.69 to 0.32
75+	70.00	64.42-76.06	55.13	50.87-59.76	2.87	2.26 - 3.48
**Net Drift (%)**					1.52	1.16 - 1.89
**Period**	Rate Ratio					
1999-2003	0.90	0.86-0.94				
2004-2008	1.00	1.00-1.00				
2009-2013	1.03	0.99-1.07				
2014- 2018	1.15	1.10-1.20				
**Cohort**	Rate Ratio					
1924-1928	0.64	0.57-0.72				
1929-1933	0.97	0.88-1.07				
1934-1938	1.04	0.96-1.13				
1939-1943	1.01	0.94-1.09				
1944-1948	0.95	0.89-1.02				
1949-1953	0.98	0.93-1.04				
1954-1959	0.99	0.94-1.04				
1959-1963	1.00	1.00-1.00				

1964-1968	1.11	1.05-1.17				
1969-1973	1.19	1.12-1.26				
1974-1978	1.36	1.27-1.46				
1979-1983	1.96	1.79-2.14				
1984-1988	2.14	1.89-2.42				
1989-1993	2.03	1.66-2.49				
1994 -1998	1.94	1.25-3.02				
1999-2003	3.04	1.00-9.27				

Table S5. Age Period Cohort effect estimates of ethnic trends in cervical cancer mortality by ethnic groups in South Africa, 1999-2018.

Factor	Black			White			Indian/Asian			Coloured		
	Longitudinal RR(adjusted for period effect) RR(95%CI)	Cross sectional RR (Adjusted for cohort effect) RR(95%CI)	Local drift(%) (95%C)	Longitudinal RR(adjusted for period effect) RR(95%CI)	Cross sectional (Adjusted for cohort effect) RR(95%CI)	Local drift(%) (95%CI)	Longitudinal RR(adjusted for period effect) RR(95%CI)	Cross sectional RR (Adjusted for cohort effect) RR(95%CI)	Local drift (%) (95% CI)	Longitudinal RR (adjusted for period effect) RR(95%CI)	Cross sectional RR (Adjusted for cohort effect) RR(95%CI)	Local drift (%) (95%CI)

Age												
15-19	0.011 (0.005-0.025)	0.04(0.02-0.09)	4.49 (-3.14to12.72)	0.0054 (0.0001-0.2872)	0.0143(0.0004-0.4612)	-7.31 (-42.13to48.45)	0.166(0.002to12.738	0.052 (0.002to1.797)	-10.41(-44.22to43.87)	0.042(0.007-0.271)	0.044 (0.008-0.255)	-11.75(-40.61to31.14)
20-24	0.100 (0.074-0.136)	0.33 (0.25-0.42)	2.33 (-0.74to5.50)	0.07 (0.03-0.17)	0.17 (0.06-0.49)	-10.12(-32.86to20.33)	0.99 (0.20to4.81)	0.36 (0.07to1.83)	-15.45(-38.70to16.60)	0.29(0.12-0.73)	0.31(0.13-0.72)	0.61(-7.40to9.32)
25-29	0.58 (0.49-0.68)	1.52(1.31-1.76)	4.38 (2.90to5.87)	0.21 (0.11-0.41)	0.44 (0.19-1.00)	3.63 (-2.37to9.99)	0.19 (0.03to1.38)	0.08 (0.01to0.56)	-10.87(-30.15to13.74)	1.18(0.80-1.77)	1.24(0.78-1.97)	4.48 (0.79to8.31)
30-34	2.32 (2.08-2.60)	4.97(4.46-5.54)	6.20 (5.30to7.11)	0.64 (0.42-0.96)	1.15 (0.61-2.17)	7.66 (4.27to11.15)	1.53(0.67to3.51)	0.75 (0.28to2.00)	0.67 (-5.83to7.62)	3.35(2.59-4.32)	3.54(2.46-5.09)	4.18(1.97to6.44)
35-39	6.37 (5.84-6.95)	11.04(10.09-12.09)	5.88 (5.21to6.56)	1.78 (1.32-2.40)	2.85 (1.72-4.73)	6.06 (3.71to8.46)	2.91 (1.59to5.31)	1.67 (0.75to3.72)	-2.14 (-7.29to3.29)	8.33(6.92-10.03)	8.87(6.62-11.88)	1.41(-0.11to2.95)
40-44	15.43 (14.42-16.50)	21.70 (20.09-23.43)	5.29 (4.72to5.87)	3.99 (3.21-4.97)	5.65 (3.83-8.35)	5.20 (3.26to7.16)	4.91 (3.09to7.78)	3.29 (1.71to6.30)	0.58 (-3.24to4.55)	15.70(13.77-17.92)	16.85 (13.44-21.11)	-0.66 (-1.83to0.52)
45-49	26.68 (25.20-28.24)	30.43(28.48-32.52)	4.49(3.97to5.02)	4.74 (3.90-5.76)	5.92 (4.42-7.94)	5.22 (3.43to7.03)	6.77 (4.67to9.83)	5.29 (3.20to8.73)	-0.51 (-3.78to2.87)	22.74(20.24-25.55)	24.58(20.75-29.12)	-1.37 (-2.42to-0.30)

50-54	37.88 (35.90-39.98)	35.05(33.02-37.21)	3.69 (3.17to 4.21)	6.91 (5.76-8.29)	7.63 (6.15-9.47)	4.19(2.45to5.97)	9.89 (7.22to13.54)	9.00 (6.09to 13.32)	-0.86 (-3.88to 2.26)	27.14(24.31-30.30)	29.56(25.98-33.64)	-0.84(-1.87to0.19)
55-59	55.08 (52.16-58.17)	41.34(39.03-43.78)	3.25 (2.72to 3.78)	8.54 (7.09-10.27)	8.33 (6.88-10.08)	2.44(0.69to4.22)	11.29 (7.97to15.99)	11.99 (8.52to 16.87)	-1.12 (-3.95to 1.78)	30.58(27.25-34.31)	33.56(29.64-38.00)	-0.68 (-1.74to0.40)
60-64	70.73 (65.76-76.07)	43.05(40.59-45.66)	2.77 (2.23to 3.32)	8.20 (6.38-10.53)	7.06 (5.54-9.00)	0.67 (-1.15to2.53)	11.40 (7.42to17.53)	14.12 (9.83to 20.29)	-2.88 (-5.65to-0.03)	31.12(26.67-36.31)	34.41(29.48-40.17)	-0.89 (-2.03to0.27)
65-69	92.88 (85.47-100.93)	45.86(42.89-49.04)	2.89 (2.31to 3.47)	7.06(5.24-9.51)	5.37 (3.82-7.56)	-1.11 (-3.07to0.90)	14.98 (9.36to23.99)	21.63 (13.55to34.54)	-4.56 (-7.39to-1.64)	35.03(29.30-41.90)	39.04(31.78-47.96)	-1.29 (-2.54to-0.03)
70-74	120.13 (109.27-132.07)	48.11(44.29-52.25)	3.45 (2.82to 4.08)	10.08 (7.32-13.88)	6.78 (4.35-10.56)	-1.87 (-3.72to0.02)	11.67 (6.60to20.62)	19.65 (10.37to37.23)	-4.47 (-7.79to-1.02)	34.34 (27.79-42.44)	38.56(29.28-50.78)	-0.94()-2.36to0.50
75+	208.39 (188.85-229.95)	67.69(61.70-74.26)	5.58 (4.81to 6.36)	13.82 (9.93-19.25)	8.21 (4.76-14.16)	-1.19(-2.91to0.58)	9.92 (5.56to17.71)	19.48 (8.95to 42.38)	-3.61 (-7.53to 0.48)	41.22(33.00-51.49)	46.64(33.36-65.21)	-0.25(-1.89to1.43)
**Net Drift (%)**			4.28 (3.84 - 4.71)			2.51 (-0.07 to 5.16)			-3.03(-6.53 to 0.61)			-0.15(-1.62 to 1.34)
**Period**	**Rate Ratio**	**95%CI**		**Rate Ratio**	**95%CI**		**Rate Ratio**	**95%CI**		**Rate Ratio**	**95%CI**	

1999-2003	0.87	0.83-0.91	0.98	0.81-1.20	1.73	1.26-2.38	1.02	0.91-1.15
2004-2008	1.00	1.00-1.00	1.00	1.00-1.00	1.00	1.00-1.00	1.00	1.00-1.00
2009-2013	1.13	1.08-1.18	1.10	0.91-1.33	1.15	0.84-1.56	0.96	0.86-1.08
2014-2018	1.68	1.60-1.76	1.44	1.10-1.89	0.99	0.66-1.50	1.01	0.86-1.19
**Cohort**	**Rate Ratio**	**95%CI**	**Rate Ratio**	**95%CI**	**Rate Ratio**	**95%CI**	**Rate Ratio**	**95%CI**
1924-1928	0.25	0.22-0.29	0.93	0.62-1.37	2.96	1.31-6.72	1.22	0.89-1.67
1929-1933	0.37	0.33-0.41	0.94	0.65-1.36	2.53	1.29-4.96	1.26	0.97-1.64
1934-1938	0.47	0.43-0.52	0.85	0.60-1.21	2.02	1.15-3.52	1.28	1.03-1.60
1939-1943	0.57	0.52-0.62	0.79	0.58-1.07	1.73	1.03-2.91	1.16	0.96-1.41
1944-1948	0.61	0.56-0.66	0.71	0.54-0.93	1.24	0.80-1.95	1.11	0.94-1.31
1949-1953	0.74	0.69-0.80	0.73	0.58-0.93	1.03	0.68-1.57	1.05	0.91-1.21
1954-1959	0.84	0.79-0.89	0.87	0.70-1.07	1.13	0.78-1.64	1.02	0.90-1.16

1959 - 1963	1.00	1.00-1.00	1.00	1.00-1.00	1.00	1.00-1.00	1.00	1.00-1.00
1964 - 1968	1.28	1.20-1.36	1.39	1.13-1.71	0.93	0.62-1.40	0.92	0.81-1.04
1969 - 1973	1.60	1.49-1.72	1.82	1.43-2.30	1.06	0.65-1.74	0.84	0.72-0.97
1974 - 1978	2.19	2.01-2.39	2.13	1.59-2.84	1.05	0.58-1.92	0.92	0.77-1.11
1979 - 1983	2.99	2.68-3.32	3.51	2.40-5.12	0.65	0.26-1.61	1.12	0.87-1.44
1984 - 1988	3.94	3.41-4.55	5.25	3.10-8.92	1.40	0.46-4.19	1.55	1.09-2.21
1989 - 1993	4.08	3.22-5.17	3.36	1.28-8.85	0.12	0.00-6.84	1.72	0.96-3.08
1994 - 1998	4.33	2.62-7.16	0.69	0.01-87.84	0.09	0.00-13.60	1.20	0.32-4.53
1999 - 2003	8.02	2.34-27.52	2.52	0.00-3957.53	0.25	0.00-476.79	0.22	0.00-158.88

Table S6. Wald Chi-square test for estimable functions of age period cohort model in the overall and ethnic trends of mortality of cervical cancers in South Africa (1999 – 2018).

Gynaecological cancer type	NetDrift = 0		All Period RR = 1		All Cohort RR = 1		All Local Drifts = Net Drift	
	Chi-square	P-value	Chi-square	P-value	Chi-square	P-value	Chi-square	P-value
**Cervix**								
Overall	66.99	2.72E-16*	77.83	8.97E-17*	374.62	1.47E-70*	238.55	1.52E-43*
Black	382.77	3.11E-85*	596.65	5.36E-129*	1100.05	4.72E-225*	91.34	7.73E-14*
White	3.65	0.056	11.26	0.01*	82.81	2.13E-11*	55.39	3.45E-07*
Indian/Asian	2.68	0.10	11.80	0.0081*	17.92	0.27	9.40	0.74
Coloured	0.040	0.84	1.81	0.61	27.58	0.02*	24.39	0.03*

*Statistically significant at P-value< 0.05
